# Thyroid MALT lymphoma: self-harm to gain potential T-cell help

**DOI:** 10.1038/s41375-021-01289-z

**Published:** 2021-05-21

**Authors:** Fangtian Wu, Natsuko Watanabe, Maria-Myrsini Tzioni, Ayse Akarca, Chunye Zhang, Yan Li, Zi Chen, Francesco Cucco, Natasha Carmell, Jaeduk Yoshimura Noh, Koichi Ito, Rachel Dobson, Sarah Moody, Wenqing Yao, Wenyan Zhang, Weiping Liu, Hongxiang Liu, Jessica Okosun, Andreas Chott, Yingwen Bi, Shih-Sung Chuang, Markus Raderer, Jian-Yong Li, Teresa Marafioti, Ming-Qing Du

**Affiliations:** 1 Division of Cellular and Molecular Pathology, Department of Pathology, University of Cambridge, Cambridge, UK; 2 Department of Hematology, Pukou CLL Center, The First Affiliated Hospital of Nanjing Medical University, Jiangsu Province Hospital, Collaborative Innovation Center for Cancer Personalized Medicine, Nanjing, PR China; 3 Department of Internal Medicine, Ito Hospital, Tokyo, Japan; 4 Department of Pathology, University College London, London, UK; 5 Department of Haematology, Hebei General Hospital, Shijiazhuang, Hebei PR China; 6 Indica Labs, Albuquerque, NM USA; 7 Department of Surgery, Ito Hospital, Tokyo, Japan; 8 Department of Pathology, West China Hospital, Sichuan University, Chengdu, PR China; 9 Molecular Malignancy Laboratory, Addenbrooke’s Hospital, Cambridge University Hospitals NHS Foundation Trust, Cambridge, UK; 10 Centre for Haemato-Oncology, Barts Cancer Institute, Queen Mary University of London, London, UK; 11 Institute of Pathology and Microbiology, Wilhelminenspital, Vienna, Austria; 12 Department of Pathology, Eye and ENT Hospital, Fudan University, Shanghai, PR China; 13 Department of Pathology, Chi-Mei Medical Centre, Tainan, Taiwan; 14 Department of Medicine I, Clinical Division of Oncology, Medical University of Vienna, Vienna, Austria; 15 Department of Histopathology, Addenbrooke’s Hospital, Cambridge University Hospitals NHS Foundation Trust, Cambridge, UK

**Keywords:** B-cell lymphoma, Cancer genetics

## Abstract

The development of extranodal marginal zone lymphoma of mucosa-associated lymphoid tissue (MALT) is driven by chronic inflammatory responses and acquired genetic changes. To investigate its genetic bases, we performed targeted sequencing of 93 genes in 131 MALT lymphomas including 76 from the thyroid. We found frequent deleterious mutations of *TET2* (86%), *CD274* (53%), *TNFRSF14* (53%), and *TNFAIP3* (30%) in thyroid MALT lymphoma. *CD274* was also frequently deleted, together with mutation seen in 68% of cases. There was a significant association between *CD274* mutation/deletion and *TNFRSF14* mutation (*p* = 0.001). CD274 (PD-L1) and TNFRSF14 are ligands for the co-inhibitory receptor PD1 and BTLA on T-helper cells, respectively, their inactivation may free T-cell activities, promoting their help to malignant B-cells. In support of this, both the proportion of activated T-cells (CD4+CD69+/CD4+) within the proximity of malignant B-cells, and the level of transformed blasts were significantly higher in cases with *CD274*/*TNFRSF14* genetic abnormalities than those without these changes. Both *CD274* and *TNFRSF14* genetic changes were significantly associated with Hashimoto’s thyroiditis (*p* = 0.01, *p* = 0.04, respectively), and *CD274* mutation/deletion additionally associated with increased erythrocyte sedimentation rate (*p* = 0.0001). In conclusion, *CD274*/*TNFRSF14* inactivation in thyroid MALT lymphoma B-cells may deregulate their interaction with T-cells, promoting co-stimulations and impairing peripheral tolerance.

## Introduction

Extranodal marginal zone lymphoma of mucosa-associated lymphoid tissue (MALT) commonly arises in a background of a chronic inflammatory disorder at diverse sites. The chronic inflammation may be caused by infection such as *Helicobacter pylori* (*H. pylori*) or autoimmunity, for example, Sjögren’s syndrome and Hashimoto’s thyroiditis. The chronic inflammatory process triggers the development of acquired MALT, which generates the “local” adaptive immune response i.e. T-cell dependent B-cell maturation [1]. These adaptive immune responses are critical for the clonal selection of marginal zone B-cells and their malignant transformation. Both B-cell receptor (BCR) signalling and T-cell help play an important role in the evolution of MALT lymphoma cells.

There are several strands of evidence indicating that BCR signalling is operational in MALT lymphoma. Histologically, the lymphoma cells always express surface immunoglobulin M (IgM), frequently show blast transformation, plasma cell differentiation, and follicular colonisation [2]. Their proliferation can be stimulated by crosslinking their surface IgM [3]. Moreover, inhibiting BCR signalling with a BTK inhibitor induces durable responses in patients with MALT lymphoma [4]. There is mounting evidence to suggest that MALT lymphoma-associated BCRs are autoreactive, albeit largely based on findings from those of the salivary gland, ocular adnexa, and stomach [1]. In ocular adnexal MALT lymphoma, there is a significant association between the biased usage of autoreactive IGHV4-34 and inactivation of *TNFAIP3* (A20) [5], which encodes a global negative regulator of the canonical NF-κB pathway. The findings suggest oncogenic cooperation between chronic BCR signalling and its downstream genetic change, thus advocating their cooperative role in clonal selection and malignant transformation.

The understanding of the role of T-cell help in MALT lymphoma pathogenesis is largely based on observations from the gastric form. Early studies show that gastric MALT lymphoma B-cells respond to *H. pylori* stimulation in vitro, but this critically depends on tumour infiltrating T-cells involving CD40/CD40L co-stimulating molecules [6–8]. Subsequent animal model studies confirm the above observations, and also demonstrate that T-helper cells are indispensable for tumour growth in vivo [9, 10]. Since T-cell dependent B-cell maturation is the cardinal feature of the adaptive immune responses, T-cell help may represent a common mechanism in the pathogenesis of MALT lymphomas regardless of their sites. T-cell help may also cooperate with BCR signalling and somatic genetic changes in the clonal evolution of lymphoma cells.

To unravel the genetic basis and improve our understanding on the oncogenic cooperation between genetic changes and tumour environment, we recently performed whole exome sequencing (WES) analyses of 21 MALT lymphomas of the salivary glands and thyroid [11]. This identified recurrent novel mutations in several genes encoding G-protein coupled receptor (GPCR), including *GPR34* and *CCR6*, and lead to the discovery of a significant association between *GPR34* and *TBL1XR1* mutations in salivary gland MALT lymphoma [11]. *GPR34* mutations are activating changes, promoting the receptor signalling, while the *TBL1XR1* mutations appear to enhance the transcriptional activities of NF-κB and AP1 by mediating nuclear receptor corepressor degradation [12]. Again, *GPR34* and *TBL1XR1* mutations are potentially cooperative events, providing another example linking surface receptor signalling to downstream genetic changes. To extend this discovery, we have designed a panel of 93 genes including GPCR genes implicated in lymphocyte biology, the genes mutated in marginal zone lymphoma and also those showing isolated but potentially pathogenic mutation from our previous WES study, such as *CD274* [11]. By targeted sequencing of this gene panel in MALT lymphoma of various sites, we have identified highly frequent inactivating mutations of both *CD274* (PD-L1) and *TNFRSF14* in thyroid MALT lymphoma. PD-L1 and TNFRSF14 inactivation in malignant B-cells may eliminate their inhibitory regulation to T-helper cells, indirectly enhancing their activities, and hence exaggerating their help to tumour B-cells.

## Materials and methods

### Case selection and materials

Local ethical guidelines were followed for the use of archival tissues for research with ethical approval (05-Q1604-10). A total of 194 cases of lymphoma were successfully investigated, including 131 MALT lymphomas (thyroid *n* = 76, ocular adnexa *n* = 30, salivary gland (mainly *GPR34* mutation negative cases) *n* = 17, others *n* = 8), splenic marginal zone lymphoma (SMZL *n* = 18), follicular lymphoma (*n* = 20), angioimmunoblastic T-cell lymphoma (AITL, *n* = 19) and monomorphic epitheliotropic intestinal T-cell lymphoma (*n* = 6) (Table S1). Formalin-fixed paraffin-embedded (FFPE) diagnostic tissue biopsies or DNA samples were available in each case. Most of the thyroid MALT lymphomas were from Ito Hospital, Tokyo, with detailed clinical and laboratory data [13], and their diagnosis was ascertained by appropriate immunohistochemical and genetic analyses, particularly to exclude follicular lymphoma.

### DNA extraction and quality assessment

For each specimen, tumour rich areas (>30%) were microdissected on FFPE slides. DNA was extracted using the QIAamp DNA Micro Kit (QIAGEN, UK) and quantified using a Qubit^®^ Fluorometer (Life Technologies, UK). The quality of DNA samples was assessed by PCR of variably sized genomic fragments [14].

### Gene panel for targeted sequencing

We comprehensively reviewed WES data on marginal zone lymphoma together with those from our previous study [11, 15]. We also reviewed the GPCR literature and identified those involved in lymphocyte biology as we recently found recurrent GPCR mutations in MALT lymphoma [11]. Based on these reviews, we established a panel of 93 genes for MALT lymphoma, comprising those mutated in marginal zone lymphoma, the GPCR genes implicated in lymphocyte biology and those showing isolated but potentially pathogenic changes from our previous WES study, such as *CD274* (Table S2) [11].

### HaloPlexHS enrichment and Illumina HiSeq sequencing

This was essentially performed as described previously using HaloPlexHS system and Illumina HiSeq4000 sequencing [14]. Experimental methods, variant calling and annotation and validation of the newly designed gene panel are detailed in the Supplementary materials (Figs. S1 and S2).

Where indicated, the variants identified by targeted sequencing were confirmed by PCR and Sanger sequencing, and their somatic origin ascertained by analysis of DNA samples from microdissected non-neoplastic cells (Table S3 and Fig. S3).

### Multiplex ligation-dependent probe amplification (MLPA)

*CD274* deletion was investigated using the MLPA assay (MRC-Holland). The MLPA assay includes multiple probes for the *CD274* (7 probes), *PDCD1LG2* (10 probes) and *JAK2* (12 probes) locus together with controls, which are located within a 0.59 Mb region at 9p24.1. DNA from FFPE tonsil tissues were utilised as a normal diploid control for normalisation. Prior to data collection, various qualities and quantities of DNA samples were tested to establish the minimal sample requirements (>30% tumour cell content, 100 ng input, strong amplification of ≥300 bp genomic fragment) for the assay. Data normalisation and analysis were performed using the Coffalyser.NET analysis software (MRC-Holland). The target/reference probe ratios were visualised using R-V3.6.1 and R studio.

### Interphase fluorescence in situ hybridisation (FISH)

In thyroid MALT lymphoma, interphase FISH was performed to investigate *MALT1*, *FOXP1* and *IGH* translocations using break-apart probes [16].

### Multiplex immunofluorescent staining (mIF)

FFPE tissue sections of thyroid MALT lymphomas and reactive tonsils were subjected to mIF staining using antibodies against Ki67/CD8, PD1, CD4, PD-L1, CD69 and CD20 sequentially (Table S4).

The antigen retrieval and mIF were performed on a Leica BOND RX automated immunostainer (Leica Microsystems, UK) using Opal 7-Colour Automation IHC Kit (Akoya Biosciences, USA). Experimental conditions, validation, imaging acquisition and data analysis optimisation are detailed in the Supplementary methods).

For data collection, tumour areas with adequate staining were identified, and representative diffuse tumour areas excluding colonised follicles were marked based on haematoxylin and eosin slide and mIF staining pattern. The expression of CD20, CD4, PD1 and CD69 and their co-expression in the selected diffuse tumour areas were quantified, and their spatial relationship was further analysed using the Halo-V3.1 Proximity Module accordingly.

### PD-L1 immunohistochemistry

This was performed on FFPE tissue sections using an automated immunostainer (Bond-III system, Leica Biosystems). Following antigen retrieval by combination of heat and Bond Epitope Retrieval 2 solution for 20 min, PD-L1 was stained with the monoclonal antibody clone E1L3N (Cell Signalling) and visualised using Bond Polymer Kit.

### Semi-quantification of histopathological features

The extent of transformed blasts, plasmacytic differentiation and follicular colonisation were semi-quantified using a three tiers system by two pathologists blindly. Any discrepancies were reviewed and rescored. The score criteria were as follows: transformed blasts: score 1 = 0–5 large cells per high power field (HPF), score 2 = 6–15 large cells per HPF, score 3 =>15 large cells per HPF; plasmacytic differentiation: score 0 = no plasmacytic differentiation, score 1 = focal or scattered plasmacytic differentiation, score 3 = diffuse areas of plasmacytic differentiation; and follicular colonisation: score 0 = no apparent follicular colonisation, score 1 = follicular colonisation in ≤50% follicles, score 2 = follicular colonisation in >50% follicles.

### Statistical analysis

Comparison of *TET2*, *CD274*, *TNFRSF14* and *TNFAIP3* mutation AAF (alternative allele frequency) values were analysed using a paired *t*-test. Association among categorical variables were analysed using Chi-square test. Comparison of the duration of Hashimoto’s thyroiditis between different mutation groups were performed using *t*-test. Comparison of semi-quantitative or quantitative phenotypical data were analysed using Wilcoxon rank sum test.

## Results

### Frequent CD274 and TNFRSF14 mutations in thyroid MALT lymphoma

**Fig. 1:**
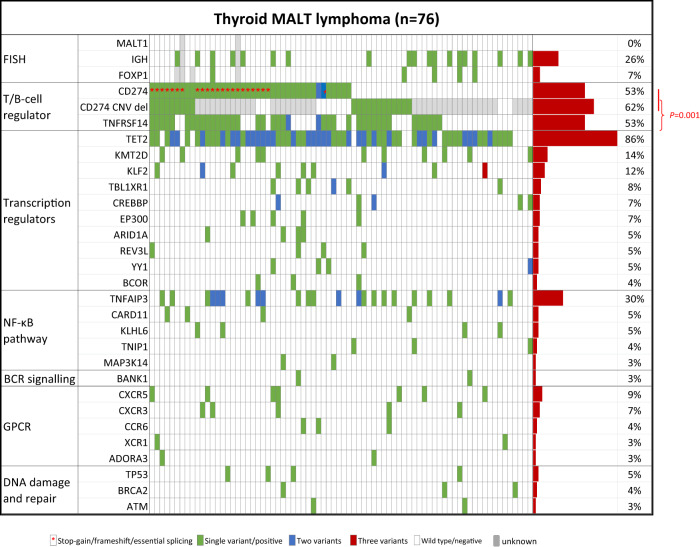
Genetic profile of thyroid MALT lymphoma. Data shown here include chromosome translocations associated with MALT lymphoma, and genes mutated at a frequency of ≥3%. *CD274* mutation/deletion, *TNFRSF14* and *TET2* mutation are most frequent, often occurring together. For complete genetic data from this study, please see Supplementary Fig. S4. CNV copy number variation.

**Fig. 2:**
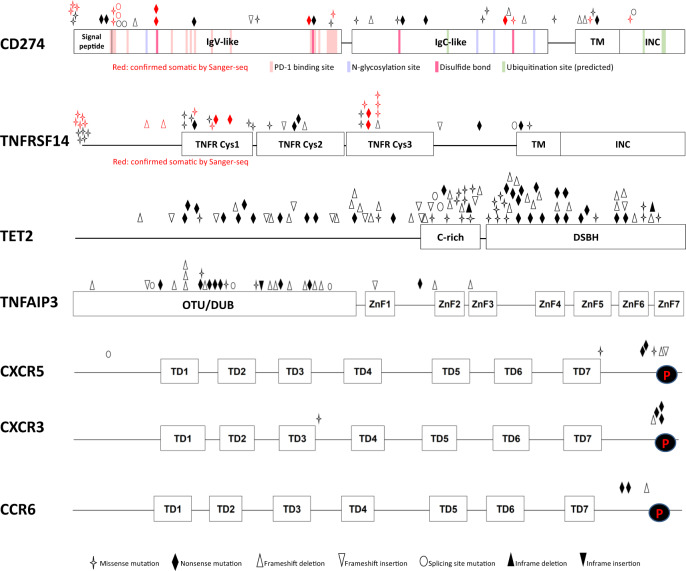
Distribution and characteristics of *CD274*, *TNFRSF14*, *TET2*, *TNFAIP3*, *CXCR5*, *CXCR3* and *CCR6* mutations in thyroid MALT lymphoma. Where possible, DNA from microdissected non-neoplastic cells was used for PCR and Sanger sequencing to exclude potential germline variants, and mutations confirmed to be somatic are indicated by symbols in red colour (Color figure online).

Among MALT lymphoma of various sites, the most prominent findings were frequent *CD274* (52.6%), *TNFRSF14* (52.6%), *TET2* (85.5%) and *TNFAIP3* (30%) mutations in the thyroid cases (Figs. 1 and S4). The former three gene mutations were not or rarely seen in MALT lymphoma of other sites. The somatic nature of *CD274* and *TNFRSF14* mutations were confirmed in 15 and 17 cases respectively, for which sufficient non-neoplastic cells could be microdissected for sequencing analysis (Figs. 2 and S3).

Most *CD274* mutations in thyroid MALT lymphoma were likely deleterious (Fig. 2 and Table S5). Of the 42 mutations detected in 40 cases, 23 were nonsense (11), frameshift indels (7) or changes involving the essential splicing site (5). These mutations were widely distributed, but always occurred upstream of the C-terminal transmembrane domain, predicting truncated products that are unlikely to be expressed on cell surface. The remaining 19 mutations were missense changes affecting the translation start site (5), PD1 binding sites (4), the transmembrane domain (2), the IgV-like or IgC-like domain (6) and regions with unknown functional domain (2).

Similarly, most *TNFRSF14* mutations in thyroid MALT lymphoma were also likely deleterious (Fig. 2 and Table S5). Of the 42 mutations identified, 16 were nonsense (9), frameshift indels (6) or changes involved the essential splicing site (1). These mutations were dispersed, but always upstream of the C-terminal transmembrane domain, predicting truncated products that are unable to be expressed on cell surface. The remaining 26 mutations were missense changes, which primarily affected the translation start site (10), or clustered at the TNFR Cys1 (8) or Cys 3 domain (6).

The *TET2* mutations seen in thyroid MALT lymphoma were very similar to those found in AITL (Fig. 2 and Table S5) [17]. Of the 65 cases with *TET2* mutations, 30 had two mutations. Of the 95 mutations detected, 71 were nonsenses (29), frameshift indels (40) or changes affecting the essential splicing site (2). These mutations were widely distributed, predicting for variably truncated protein products. The remaining 24 mutations were missense changes (22) and inframe deletions (2), and largely clustered in the cysteine rich and double‐stranded β helix domains, which were essential for the integrity of the overall structure and the catalytic activity of TET2 [18].

*TNFAIP3* was also frequently mutated in thyroid MALT lymphoma. A total of 31 *TNFAIP3* mutations were seen in 23 cases, and they comprised of nonsenses (8), frameshift indels (16) and changes affecting the essential splicing site (3) (Fig. 2 and Table S5). These mutations were widely dispersed, predicting variably truncated protein products. The remaining four mutations were missense changes (3) and inframe insertion (1) in the OTU domain.

Mutations in other genes at ≥3% frequency identified in thyroid MALT lymphoma are shown in Fig. 1, with full data presented in Fig. S4. Among the 60 GPCR genes investigated, recurrent changes were seen in *CXCR5*, *CXCR3* and *CCR6* and characterised by clustered deleterious mutations in their C-terminal sequence but upstream of the phosphorylation site (Fig. 2), which mediates interaction with β-arrestin and receptor internalisation [19].

Interphase FISH showed a low frequency (7%) of *FOXP1*, but not *MALT1* translocation in thyroid MALT lymphoma (Fig. 1). *IGH* translocation was seen in 26% of cases, including four of the five cases with *FOXP1* translocation.

### High *TET2* and *TNFRSF14* mutation AAF in thyroid MALT lymphoma

**Fig. 3:**
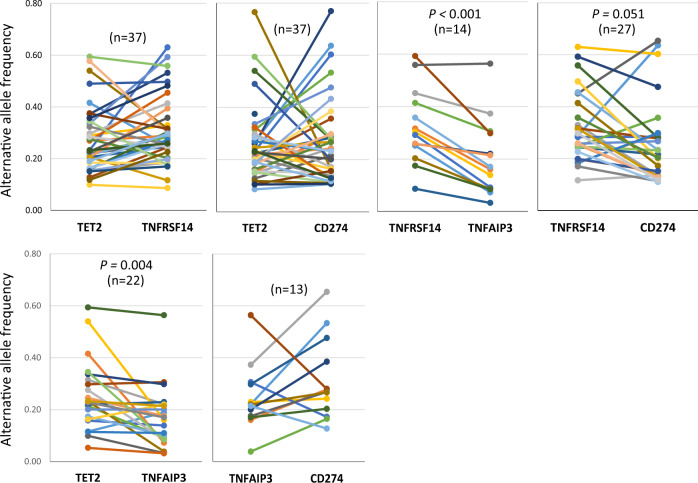
Comparison of *CD274*, *TNFRSF14*, *TET2* and *TNFAIP3* mutation AAF (alternative allele frequency) in thyroid MALT lymphoma. Both *TET2* and *TNFRSF14* have a significantly higher mutation AAF than *TNFAIP3*. *TNFRSF14* also has a higher mutation AAF than *CD274*, although not statistically significant.

*TET2* mutation occurs early in haematopoietic stem cells, typically in individuals with clonal haematopoiesis of indeterminate potential. We correlated *TET2*, *TNFRSF14*, *CD274* and *TNFAIP3* mutation AAF in thyroid MALT lymphoma in order to understand the sequence of their occurrence (Fig. 3). There was no difference in the AAF values between *TET2* and *CD274*, nor between *TET2* and *TNFRSF14*, suggesting that the *TET2* mutations seen in thyroid lymphoma were most likely lymphoma cell specific. Both *TET2* and *TNFRSF14* had a significantly higher AAF than *TNFAIP3*, suggesting that *TNFAIP3* mutations may occur later than *TET2* and *TNFRSF14* changes.

**Fig. 4:**
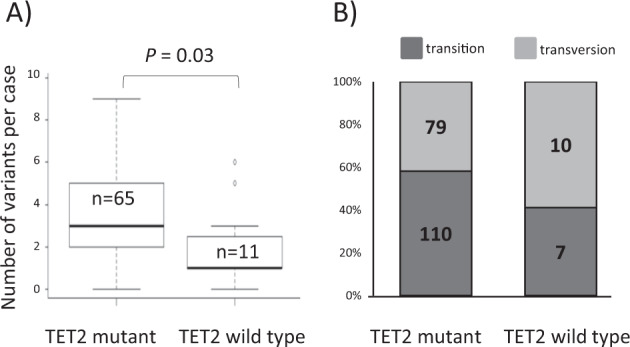
Comparison of mutation burden and characteristics according to *TET2* mutation status in thyroid MALT lymphoma. *TET2* mutation is excluded from the mutation calculation. **A** The overall mutation load is significantly higher in the cases with *TET2* mutation than those without the mutation (*p* = 0.03). **B** The proportion of transition mutations is also higher in cases with *TET2* mutation than those without the mutation, although not statistically significant.

As TET2, a methylcytosine dioxygenase, may influence DNA mutagenicity, and Tet deficient germinal centre B-cells showed hypersomatic mutations skewing towards transition changes [20, 21], we compared mutation burden and spectrum according to the *TET2* mutation status in thyroid MALT lymphoma (Fig. 4). The number of somatic variants (excluding SNPs) was significantly higher in the cases with *TET2* mutation than those without the mutation (*p* = 0.03). The proportion of transition mutations was also higher in cases with *TET2* mutation than those without the mutation, although not reaching a statistical significance (Fig. 4).

### *CD274* is also frequently targeted by deletion

As *CD274* mutations were most likely inactivating changes, we further investigated whether *CD274* was targeted by deletion. Of the 34 cases successfully investigated by MLPA, 21 showed deletion involving the *CD274* locus. In 18 cases, the deletion spanned a region from *JAK2* exon-4 to *CD274* exon 2 or 3, and appeared to be heterozygous. While in the remaining 3 cases, the deletion involved *JAK2*, whole *CD274* and most of *PDCD1LG2* locus, and was homozygous. *CD274* deletion was much higher in cases with wild-type *CD274* than those with *CD274* mutation (80% vs 47%, *p* = 0.079) (Figs. 1 and S5). As expected, there was a mutual exclusion between *CD274* double mutations and homozygous deletion (Fig. 1). Taken together, 68% of thyroid MALT lymphomas had *CD274* mutation or deletion or both. This frequency was likely underestimated as the deletion was not investigated in a high proportion of cases with wild-type *CD274* due to suboptimal DNA quality.

Interestingly, *CD274* mutation/deletion were significantly associated with *TNFRSF14* (*p* = 0.0013), but not *TET2* mutation (*p* = 0.31). There was no association between the *TNFRSF14* and *TET2* mutations.

### Absence or low level of PD-L1 expression in thyroid MALT lymphoma

PD-L1 immunohistochemistry showed no detectable protein expression in the tumour cells of 51 thyroid MALT lymphomas investigated including 35 cases with *CD274* mutation/deletion (Fig. S6). In each case, PD-L1 expression was seen in germinal centre macrophages, the thyroid epithelial cells involved in lymphoepithelial lesions but not in intact thyroid follicle.

### Increased activated T-cells in the proximity of tumour B-cells in cases with *CD274/TNFRSF14* genetic changes

**Fig. 5:**
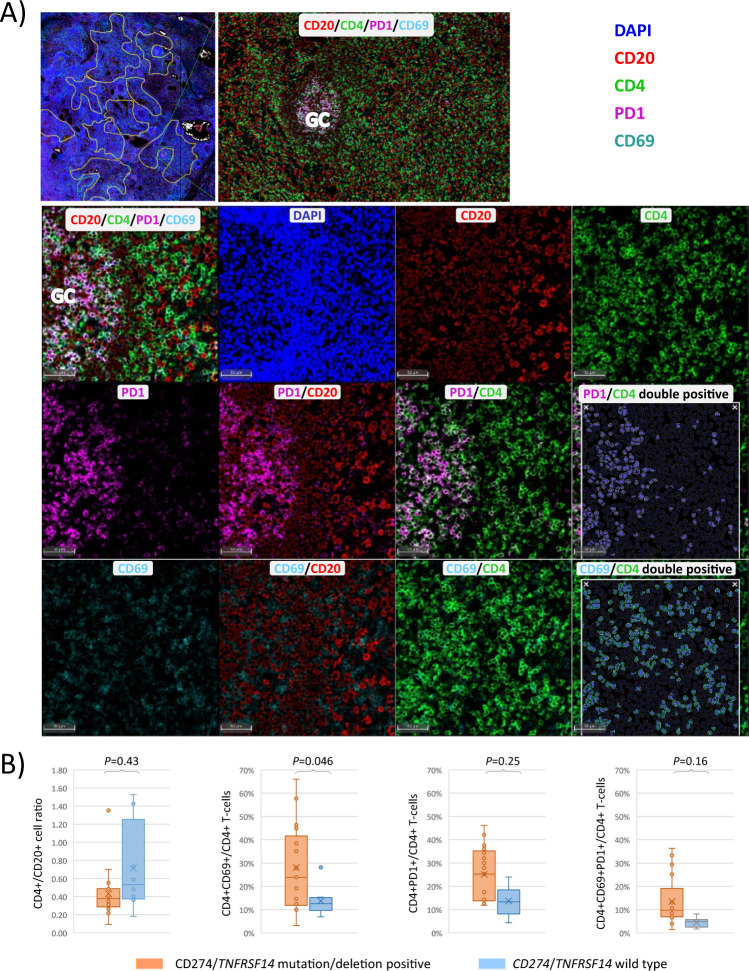
Multiplex immunofluorescent staining reveals increased activated T-cells in the vicinity of malignant B-cells harbouring *CD274*/*TNFRSF14* genetic changes. **A** An example of multiplex immunofluorescent staining in a case of thyroid MALT lymphoma with both *CD274* and *TNFRSF14* genetic changes; Diffuse tumour areas are marked, and analysed using Halo-V3.1 HighPlex FL and proximity modules. GC germinal centre. **B** Quantitative analysis CD4+/CD20+ cell ratio (left panel), and various CD4+ immunophenotypic subsets within 10 µm of CD20+ B-cells. The proportion of activated T-cells (CD4+CD69+/CD4+) is significantly higher in cases with *CD274*/*TNFRSF14* genetic abnormalities than those without these changes.

A total of 23 cases were successfully investigated by mIF including 17 cases with *CD274* mutation/deletion and/or *TNFRSF14* mutation, and six cases without these genetic changes (Fig. 5). Overall, there were no significant differences in the ratio of CD4+ T-cells/CD20+ B-cells (Fig. 5B), and the proportion of CD4+CD69+/CD4+, CD4+PD1+/CD4+ and CD4+CD69+PD1+/CD4+T-cells between cases with and without *CD274*/*TNFRSF14* genetic changes.

As the effect of PD-L1 (CD274)/TNFRSF14 inactivation to other cells is likely to be within the proximity of malignant B-cells, we quantified CD4+ T-cells and their immunophenotypic subsets (CD4+CD69+, CD4+PD1+, CD4+CD69+PD1+) within 10 µm of CD20+ B-cells using Halo-V3.1 proximity module. Interestingly, the proportion of CD4+CD69+/CD4+ T-cells within 10 µm proximity of B-cells was significantly higher in cases with *CD274*/*TNFRSF14* genetic abnormalities than those without these genetic changes (Fig. 5). There was a similar trend for the proportion of CD4+PD1+ and CD4+CD69+PD1+ T-cell subsets, but not reaching a statistical significance (Fig. 5B).

### *CD274/TNFRSF14* genetic changes significantly associate with increased transformed blasts

**Fig. 6:**
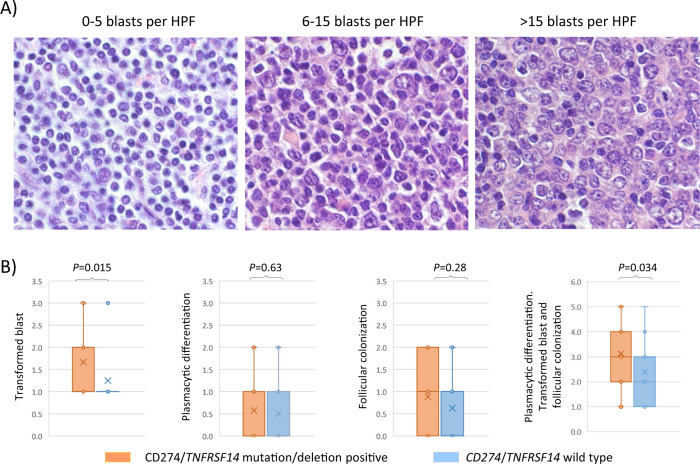
Correlation between *CD274*/*TNFRSF14* genetic changes and histological features. **A** Examples of grading for semi-quantification of transformed blasts in thyroid MALT lymphoma; HPF high power field. **B** Comparison of the extent of transformed blast, plasmacytic differentiation and follicular colonisation between cases with and without *CD274*/*TNFRSF14* genetic changes. The extent of these histological features was scored as described in the “Method” and compared using the Wilcoxon rank sum test.

Several histological features of MALT lymphoma including blast transformation, plasmacytic differentiation and follicular colonisation are likely driven by antigenic stimulation, possibly involving T-cell help. Among these features, increased transformed blasts were significantly higher in cases with *CD274*/*TNFRSF14* genetic changes than those without these changes (Fig. 6). There was also an association between increased transformed blasts and elevated serum thyroid stimulating hormone and lactate dehydrogenase, and between follicular colonisation and increased serum soluble IL2R (Table S6).

### Correlation among genetic and clinical parameters

*CD274* mutation/deletion was significantly associated with advanced age, Hashimoto’s thyroiditis and increased erythrocyte sedimentation rate (Table S6). Similarly, *TNFRSF14* mutation was significantly associated with Hashimoto’s thyroiditis. However, there was no association between *TET2* mutation and the clinical and laboratory parameters investigated.

## Discussion

The present study shows for the first time frequent *CD274* (PD-L1) inactivation by mutation and deletion in a human tumour, i.e. thyroid MALT lymphoma. Remarkably, *CD274* mutations and deletions are significantly associated with a loss of function mutations of *TNFRSF14* in this low grade B-cell lymphoma, which typically arises from a background of autoimmune Hashimoto’s thyroiditis. Given that PD-L1 and TNFRSF14 are ligands for co-inhibitory receptors PD1 and BTLA on T-helper cells respectively, their inactivation may free T-cell activities and enhance their help to malignant B-cells. This speculation is supported by findings of higher proportions of activated T-cells in the vicinity of malignant B-cells, as well as increased levels of transformed blasts in cases with *CD274*/*TNFRSF14* genetic abnormalities.

*CD274* (*PD-L1*) inactivation by mutation/deletion is highly restricted to thyroid MALT lymphoma, and rarely seen in MALT lymphoma of other sites or SMZL (Fig. S4). In fact, these *PD-L1* genetic changes have not been reported in any human cancers despite the high volume of sequencing. On the contrary, *PD-L1* is targeted for over-expression by 9p24.1 amplification or chromosome translocation in a variety of solid tumours and lymphomas, particularly mediastinal large B-cell lymphoma and classic Hodgkin lymphoma [22–26]. This forms the molecular basis for immunotherapy with immune checkpoint inhibitors. These seemingly paradoxical observations strongly suggest a unique combination of aetiological, biological and oncogenic events during the multistage development of thyroid MALT lymphoma.

**Fig. 7:**
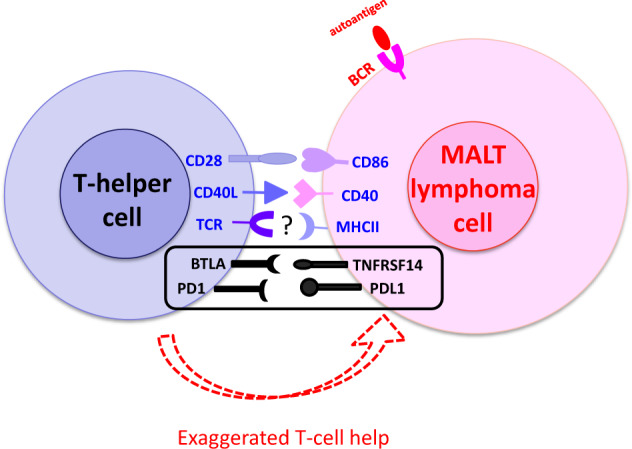
Working model of molecular mechanisms underlying thyroid MALT lymphoma. Inactivation of both *CD274* (PD-L1) and *TNFRSF14* in the lymphoma B-cells abolish their inhibitory regulation to T-helper cells, thus liberating T-cell function, leading to exaggerated T-cell help signals to the lymphoma B-cells.

B and T-cells regulate each other’s function during adaptive immune responses through concerted actions of their surface co-stimulatory [CD40/CD40L, CD80(CD86)/CD28] and co-inhibitory molecules (PD-L1/PD1, TNFRSF14/BTLA). Binding PD1 by PD-L1 attenuates T-cell receptor (TCR) signalling and suppresses T-cell expansion and cytokine production. In mice with mixed bone marrow chimeras, PD-L1 deficient B-cells out-compete their wild-type counterpart in the germinal centre, memory and plasma cell compartments [27]. Conditional knockout of PD-L1 expression in B-cells does not affect B-cell development, but promotes earlier onset and increased inflammatory foci of experimental autoimmune encephalomyelitis in mice [28]. Similarly, binding BTLA by TNFRSF14 attenuates T-cell activation by reducing TCR signalling and CD40/CD40L interactions at the immunological synaptic interface, thus restraining its help to B-cells [29]. Tnfrsf14 deficient B-cells in mice show an enhanced growth advantage due to increased CD40/CD40L co-stimulation, and Tnfrsf14 deficiency cooperates with Bcl2 over-expression in lymphomagenesis [29]. Taken together, inactivation of both *CD274* (PD-L1) and *TNFRSF14* in thyroid MALT lymphoma most likely abolishes their negative regulation to T-helper cells, hence enhances their function, leading to exaggerated T-cell help to support malignant B-cells (Fig. 7).

PD1 governs the localisation and function of follicular T-helper cells (TFH) in adaptive immune response. During the germinal centre reaction, PD1/PD-L1 interactions between TFH and B-cells modulate B-cell competition and affinity maturation [27]. PD1/PD-L1 interactions between B- and T-cells also regulates their peripheral tolerance [30], and disruption of the PD1/PD-L1 axis can cause a range of autoimmune disorders. In this context, it is worth noting that *PD-L1* mutation/deletion is significantly associated with autoimmune Hashimoto’s thyroiditis in patients with thyroid MALT lymphoma. Remarkably, autoimmune thyroiditis and appearance of autoantibodies including anti-thyroperoxidase and anti-thyroglobulin are frequently seen in cancer patients treated with checkpoint inhibitors, particularly with anti-PD1 antibody [31, 32]. Thus, PD-L1 inactivation in thyroid MALT lymphoma may impair peripheral tolerance, contributing to the autoimmunity commonly associated with these patients.

There is no detectable PD-L1 expression by immunohistochemistry in thyroid MALT lymphoma, irrespective of *CD274* genetic changes. PD-L1 expression is also not detectable by immunohistochemistry in a range of other low grade B-cell lymphomas as well as in reactive B-cells [33, 34]. These findings suggest the absence or a low level of PD-L1 expression that is beyond the sensitivity of immunohistochemistry. In view of the high (constitutive) expression of PD1 in TFH, it is plausible that B-cells may have tightly regulated PD-L1 expression, at a low level, to modulate its interaction with TFH and hence coordinate their cellular activities. In keeping with the above speculations, the proportion of activated T-cells (CD4+CD69+/CD4+) within the vicinity of malignant B-cells was significantly higher in thyroid MALT lymphoma with *CD274*/*TNFRSF14* inactivation changes than those without these abnormalities. Moreover, the level of transformed blasts was also significantly higher in cases with *CD274*/*TNFRSF14* inactivation changes than those without these abnormalities. Taken together, *CD274*/*TNFRSF14* inactivation in malignant B-cells likely deregulates their interactions with T-cells, promoting their co-stimulations.

The finding of remarkably variable involvement of *TET2* mutations in MALT lymphoma of different sites (86% in thyroid, but <8% in other sites) is intriguing as the mutation commonly occurs in haematopoietic stem/progenitor cells in individuals with clonal haematopoiesis of indeterminate potential [11, 35]. Nonetheless, TET2 has a plethora of biological activities as it facilitates DNA demethylation and promotes a permissive chromatin state for transcriptional activities. Apart from a global impact on DNA methylation and gene expression profile, TET2 also has a locus specific effect, such as regulation of the expression of transcriptional factors critical for B-cell maturation during the germinal centre reaction [20]. *TET2* inactivation by mutation may deregulate the expression of transcriptional factors important for B-cell function, and thus potentially cooperate with receptor signalling, including those by the enhanced T-helper cell signals, indirectly triggered by PD-L1/TNFRSF14 inactivation in malignant B-cells.

Differential diagnosis between thyroid MALT lymphoma and follicular lymphoma could be a challenge, particularly when MALT lymphoma shows prominent follicular colonisation. In most cases, their differential diagnosis could be resolved by carefully integrated investigations of histopathology, immunophenotype and *BCL2* and *BCL6* translocations [36]. If this conventional approach fails, somatic mutation analysis should assist their differential diagnosis, in light of the remarkable differences in the mutation profile between thyroid MALT lymphoma and follicular lymphoma [37–39], particulalry the highly frequent *CD274* and *TET2* mutations in the former. While *TNFSRF14* mutations are frequent in both MALT and follicular lymphomas, thus offering little value in their differential diagnosis.

In conclusion, thyroid MALT lymphoma is characterised by frequent and concurrent genetic inactivation of both *CD274* and *TNFRSF14*. Their inactivation most likely eliminates their inhibitory regulation to T-helper cells, consequently freeing T-cell function, and providing exaggerated T-cell help to the lymphoma B-cells. The impaired PD1/PD-L1 interaction may also debilitate peripheral tolerance and contribute to the autoimmunity in patients with thyroid MALT lymphoma. The molecular mechanisms entailed by these genetic changes provide a basis for the development of therapeutic strategies for patients with thyroid MALT lymphoma and Hashimoto’s thyroiditis.

## Supplementary information

Supplementary Materials

Supplementary figure-1

Supplementary figure-2

Supplementary figure-3

Supplementary figure-4

Supplementary figure-5

Supplementary figure-6

Supplementary table S1

Supplementary table S2

Supplementary table S3

Supplementary table S4

Supplementary table S5

Supplementary table S6
