# Supplementary material for: Thyroid MALT lymphoma: self-harm to gain potential T-cell help
Source: Leukemia. 2021 May 21;35(12):3497–508. doi: 10.1038/s41375-021-01289-z (PMC8632687; doi:10.1038/s41375-021-01289-z)
Supplement: Supplementary file 1 — Supplementary Materials [file 41375_2021_1289_MOESM1_ESM.docx]

**SUPPLEMENTARY METHODS**

**HaloPlexHS enrichment and Illumina HiSeq sequencing**

This was essentially performed as described previously [1]. Briefly, 100ng genomic DNA was subjected to targeted enrichment for 93 genes using the HaloPlexHS system that incorporates molecular barcodes (Agilent Technologies). Library preparation was performed according to the manufacturer’s instructions. The pooled libraries were sequenced on an Illumina HiSeq4000 (2 × 150bp pair-end protocol). As in our previous study, DNA samples amenable for PCR of ≥400bp genomic fragments were investigated in a single replicate, while those amenable for PCR of 300bp were analysed in duplicates (Figure S1) [1,2].

**Variant calling and annotation**

Sequence data analyses were performed using an in-house protocol established in our recent study [1,2]. Briefly, single nucleotide variants (SNV) were called using UnifiedGenotyper and additionally MuTect2 for variants at low alternative allele frequencies (AAF), while indels were identified on the recalibrated bam files using Pindel v0.2.5. Variants were filtered using vcftools v0.1.15 and bedtools v2.25 for read depth, quality score, and known PCR/sequence artefacts. Further filtering was performed to remove variants in intronic regions outside essential splicing sites, SNPs with a minor allele frequency ≥0.1% (dbSNP database, 1000 Genomes Project, the ExAC exome sequencing database) and synonymous changes. Missense variants predicted to be benign by 7 of 9 prediction tools (SIFT, Polyphen2 HDIV, Polyphen2 HVAR, LRT, MutationTaster, MutationAssessor, FATHMM, SVM score and LR score) were excluded. The resulting variants were further checked by reviewing the bam file to eliminate potential PCR and sequence artefacts (Figure S2). Only variants above the cut-off value (20 alternative allele depth for DNA samples amenable for PCR of ≥400bp, 15 alternative allele depth in both replicates for DNA samples amenable for PCR of 300bp) were considered to be a true change [1]. Finally, extensive search of the COSMIC database and published literature was performed to retain those confirmed to be somatic variants.

Where indicated, the variants identified by targeted sequencing were confirmed by PCR and Sanger Sequencing, and their somatic origin ascertained by analysis of DNA samples from microdissected non-neoplastic cells (Table S3, Figure S3).

**Validation of the targeted sequencing panel**

Prior to data collection, we optimised the experimental protocols by sequencing representative FFPE DNA samples including 89 cases with known mutations in the genes included in the current panel [3-6]. All the known mutations including 52 SNV and 26 indels across 21 genes were correctly identified. The performance of the new panel, targeted sequencing and variant calling protocol were further ascertained by the detection of the expected mutations in ocular adnexa MALT lymphoma (*TNFAIP3*: 36.7%), follicular lymphoma (*KMT2D*: 85%, *CREBBP*: 55%) and AITL (*TET2*: 73.7%) (Figure S4).

**Multiplex immunofluorescent staining (mIF)**

FFPE tissue sections of thyroid MALT lymphomas and reactive tonsils were subjected to mIF staining using antibodies against Ki67/CD8, PD1, CD4, PD-L1, CD69 and CD20 sequentially (Table S4).  The experimental conditions (antibody dilution, order of immunostaining, choice of Opal fluorophore etc) of mIF staining were optimised and validated against singleplex chromogenic immunostaining protocols on consecutive sections of reactive tonsil and lymphoma specimens. The antigen retrieval and mIF were performed on a Leica BOND RX automated immunostainer (Leica Microsystems, UK) using Opal 7-Color Automation IHC Kit (Akoya Biosciences, USA).

mIF stained slides were scanned using the Vectra 3 quantitative imaging system with data acquisition at 20× magnification. The digital data were analysed using Halo-V3.1 (Indica labs). All mIF stained slides were checked for staining quality, and any slide with unsatisfactory staining was rejected. PD-L1, Ki67 and CD8 were excluded from analysis as PD-L1 staining was difficult to interpret, and Ki67 nuclear and CD8 cytoplasmic staining could not be reliably distinguished due to the use of the same fluorophore. Prior to any data collection, the optimal algorithm for defining staining positivity was established through comprehensive testing of each parameter in the Halo-V3.1 HighPlex FL menu by real-time tuning and analyses of 10 representative mIF slides. The optimised parameters were applied to all mIF slides with the cytoplasmic positivity threshold further tuned for some of the slides due to variations in staining intensity.

1. Cucco F, Clipson A, Kennedy H, Sneath Thompson J, Wang M, Barrans S *et al.* Mutation screening using formalin-fixed paraffin-embedded tissues: a stratified approach according to DNA quality. Lab Invest*.* 2018;98**:**1084-1092.

2. Cucco F, Barrans S, Sha C, Clipson A, Crouch S, Dobson R *et al.* Distinct genetic changes reveal evolutionary history and heterogeneous molecular grade of DLBCL with MYC/BCL2 double-hit. Leukemia*.* 2020;34**:**1329-1341.

3. Moody S, Escudero-Ibarz L, Wang M, Clipson A, Ochoa Ruiz E, Dunn-Walters D *et al.* Significant association between TNFAIP3 inactivation and biased immunoglobulin heavy chain variable region 4-34 usage in mucosa-associated lymphoid tissue lymphoma. J Pathol*.* 2017;243**:**3-8.

4. Moody S, Thompson JS, Chuang SS, Liu H, Raderer M, Vassiliou G *et al.* Novel GPR34 and CCR6 mutation and distinct genetic profiles in MALT lymphomas of different sites. Haematologica*.* 2018;103**:**1329-1336.

5. Clipson A, Wang M, de Leval L, Ashton-Key M, Wotherspoon A, Vassiliou G *et al.* KLF2 mutation is the most frequent somatic change in splenic marginal zone lymphoma and identifies a subset with distinct genotype. Leukemia*.* 2015;29**:**1177-1185.

6. Yao WQ, Wu F, Zhang W, Chuang SS, Thompson JS, Chen Z *et al.* Angioimmunoblastic T-cell lymphoma contains multiple clonal T-cell populations derived from a common TET2 mutant progenitor cell. J Pathol*.* 2020;250**:**346-357.

**SUPPLEMENTARY FIGURES**

**Supplementary Figure S1.** Performance data of 93-gene panel sequencing in thyroid MALT lymphoma. Average depth read and sequence coverage with reads >50 are shown. DNA quality was assessed by a standardised quality control PCR, and 29 samples with suboptimal coverage and/or variants of uncertain (potential false positive) were investigated by the panel sequencing twice.

**Supplementary Figure S2.** Examples of mutations identified by HaloPlex target enrichment and Illumina Hiseq sequencing in thyroid MALT lymphoma. Aligned reads were transformed to a bam file and visualised using IGV software.

**Supplementary Figure S3.** Confirmation of *CD274* and *TNFRSF14* somatic mutations in thyroid MALT lymphoma by Sanger sequencing of paired tumour and non-tumour DNA.

**Supplementary Figure S4.** Mutation profile in various lymphoma entities investigated by 93 gene panel sequencing. Other comprises MALT lymphoma of the lung (n=2) and soft tissue (n=1). TH_MALT: thyroid MALT lymphoma; OA_MALT: ocular adnexal MALT lymphoma; SA_MALT: salivary gland MALT lymphoma; GA_MALT: Gastric MALT lymphoma; FL: follicular lymphoma; SMZL: splenic marginal zone lymphoma; AITL: angioimmunoblastic T-cell lymphoma; MEITL: monomorphic epitheliotropic intestinal T-cell lymphomas.

**Supplementary Figure S5.** Detection of CD274 deletion in thyroid and salivary gland MALT lymphoma by multiplex ligation-dependent probe amplification (MLPA). A) A schematic illustration of the three genes covered by the MLPA probes. B) Heatmap illustration of the normalised amplification signal. Each column denotes a case (TM: thyroid MALT lymphoma; SM: salivary gland MALT lymphoma).

**Supplementary Figure S6.** Absence of PD-L1 expression in thyroid MALT lymphoma. A representative case shows negative PD-L1 immunostaining in malignant B-cells and most of intact thyroid epithelial cells, but positive staining in epithelial cells involved in lymphoepithelial lesions.

**SUPPLEMENTARY TABLES**

**Supplementary Table S1:** Lymphoma entities and number of cases successfully investigated.

**Supplementary Table S2:** List of 93-genes investigated by targeted sequencing.

**Supplementary Table S3.** Primers used for PCR and Sanger sequencing of the *CD274* and *TNFRSF14* genes.

**Supplementary Table S4**: Antibodies and reagents used for immunoflourescence or immunohistochemical staining

**Supplementary Table S5:** Variants detected by 93-gene panel sequencing.

**Supplementary Table S6**. Clinical features and correlations with *CD274/TNFRSF14/TET2* changes and histological features.
