# Supplementary material for: Thyroid MALT lymphoma: self-harm to gain potential T-cell help
Source: Leukemia. 2021 May 21;35(12):3497–508. doi: 10.1038/s41375-021-01289-z (PMC8632687; doi:10.1038/s41375-021-01289-z)
Supplement: Supplementary file 2 — Supplementary figure-1 [file 41375_2021_1289_MOESM2_ESM.pptx]

## Slide 1
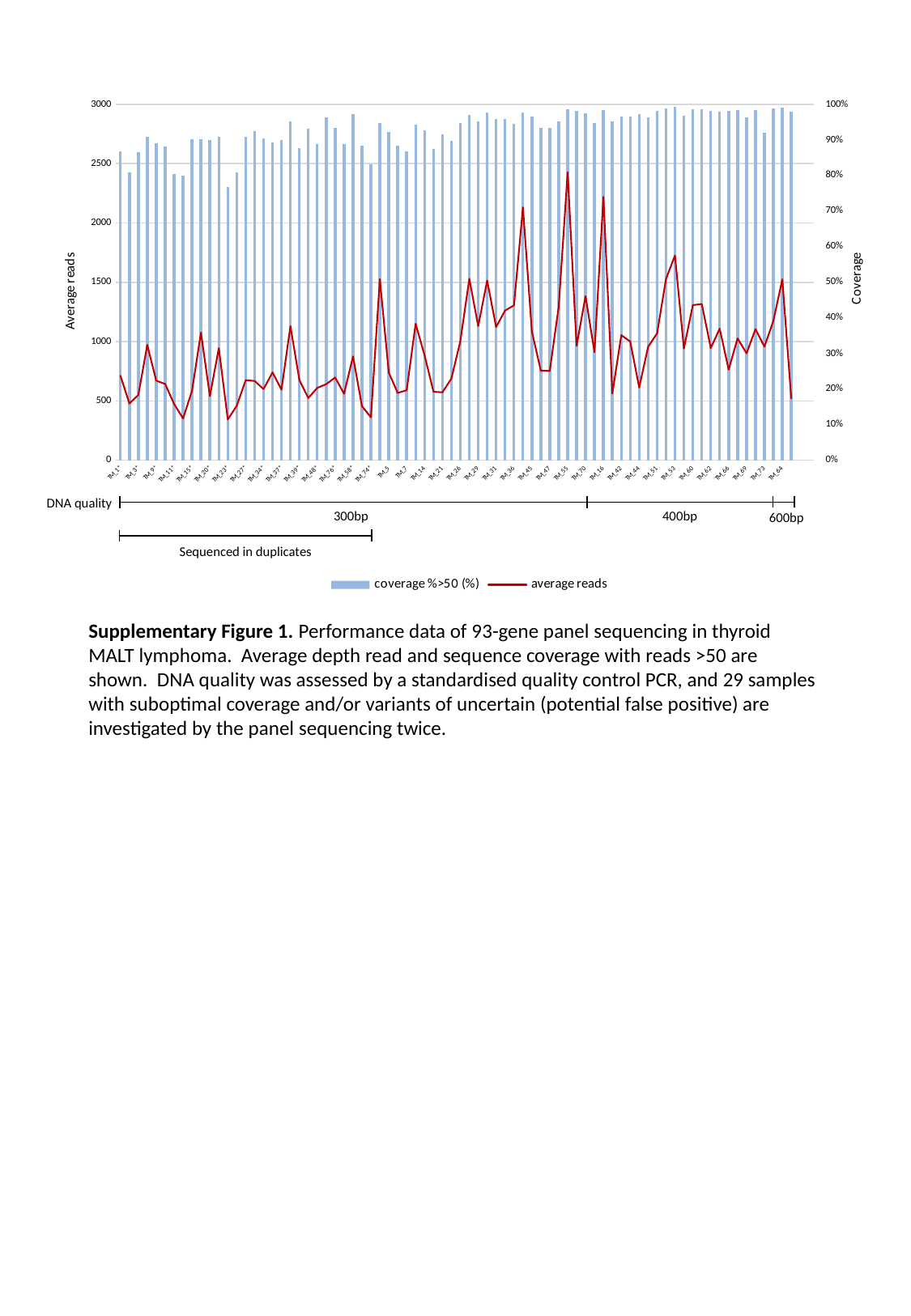

### Chart
| Category | coverage %>50 (%) | average reads |
|---|---|---|
| TM_1* | 0.867 | 712.4 |
| TM_2* | 0.80925 | 478.27 |
| TM_3* | 0.8662000000000001 | 550.53 |
| TM_8* | 0.9078499999999999 | 972.4300000000001 |
| TM_9* | 0.8896999999999999 | 670.845 |
| TM_10* | 0.8817499999999999 | 642.57 |
| TM_11* | 0.8047 | 474.46500000000003 |
| TM_13* | 0.7996500000000001 | 351.885 |
| TM_15* | 0.9028499999999999 | 584.05 |
| TM_19* | 0.90215 | 1076.54 |
| TM_20* | 0.8992 | 540.7950000000001 |
| TM_22* | 0.9086 | 944.8050000000001 |
| TM_23* | 0.7666499999999999 | 343.46999999999997 |
| TM_25* | 0.8090999999999999 | 459.19 |
| TM_27* | 0.9089 | 675.23 |
| TM_32* | 0.9239 | 668.0799999999999 |
| TM_34* | 0.9047499999999999 | 599.36 |
| TM_35* | 0.8931 | 741.115 |
| TM_37* | 0.8995000000000001 | 594.75 |
| TM_38* | 0.95195 | 1131.51 |
| TM_39* | 0.87725 | 675.555 |
| TM_41* | 0.93195 | 524.2 |
| TM_48* | 0.8892 | 609.46 |
| TM_54* | 0.96245 | 640.875 |
| TM_76* | 0.9331999999999999 | 697.52 |
| TM_57* | 0.88945 | 560.03 |
| TM_58* | 0.9724 | 875.21 |
| TM_63* | 0.8841 | 456.14 |
| TM_74* | 0.8308500000000001 | 361.745 |
| TM_4 | 0.9473999999999999 | 1526.88 |
| TM_5 | 0.9233 | 738.12 |
| TM_6 | 0.8839 | 567.36 |
| TM_7 | 0.8687 | 589.73 |
| TM_12 | 0.9437000000000001 | 1150.67 |
| TM_14 | 0.9279999999999999 | 884.93 |
| TM_17 | 0.8744 | 576.97 |
| TM_21 | 0.9158 | 572.76 |
| TM_24 | 0.8981999999999999 | 688.09 |
| TM_26 | 0.9483 | 1003.72 |
| TM_28 | 0.9695999999999999 | 1529.55 |
| TM_29 | 0.9529000000000001 | 1131.03 |
| TM_30 | 0.9763 | 1514.93 |
| TM_31 | 0.9598 | 1123.55 |
| TM_33 | 0.9590000000000001 | 1261.61 |
| TM_36 | 0.945 | 1306.21 |
| TM_75 | 0.9763 | 2131.7 |
| TM_45 | 0.9668000000000001 | 1085.89 |
| TM_46 | 0.9342 | 755.03 |
| TM_47 | 0.9331999999999999 | 752.48 |
| TM_49 | 0.9521 | 1293.4 |
| TM_55 | 0.9864 | 2427.5 |
| TM_67 | 0.9826 | 963.78 |
| TM_70 | 0.9741 | 1383.8 |
| TM_18* | 0.9481 | 910.94 |
| TM_16 | 0.9848 | 2220.59 |
| TM_40 | 0.953 | 561.9 |
| TM_42 | 0.9647 | 1054.68 |
| TM_43 | 0.9662000000000001 | 1000.79 |
| TM_44 | 0.9714 | 613.32 |
| TM_50 | 0.9643999999999999 | 955.93 |
| TM_51 | 0.9823000000000001 | 1069.09 |
| TM_52 | 0.9886 | 1528.3 |
| TM_53 | 0.9921 | 1725.79 |
| TM_59 | 0.9681000000000001 | 941.69 |
| TM_60 | 0.985 | 1307.96 |
| TM_61 | 0.9856 | 1316.62 |
| TM_62 | 0.9806999999999999 | 945.53 |
| TM_65 | 0.9802 | 1109.75 |
| TM_66 | 0.9819 | 763.95 |
| TM_68 | 0.9826999999999999 | 1027.27 |
| TM_69 | 0.9641 | 901.13 |
| TM_72 | 0.9832 | 1105.48 |
| TM_73 | 0.9198999999999999 | 956.84 |
| TM_56 | 0.9879000000000001 | 1175.04 |
| TM_64 | 0.9897 | 1524.58 |
| TM_71 | 0.9798 | 521.82 |DNA quality
300bp
400bp
600bp
Sequenced in duplicates
Supplementary Figure 1. Performance data of 93-gene panel sequencing in thyroid MALT lymphoma. Average depth read and sequence coverage with reads >50 are shown. DNA quality was assessed by a standardised quality control PCR, and 29 samples with suboptimal coverage and/or variants of uncertain (potential false positive) are investigated by the panel sequencing twice.
