# Supplementary material for: Thyroid MALT lymphoma: self-harm to gain potential T-cell help
Source: Leukemia. 2021 May 21;35(12):3497–508. doi: 10.1038/s41375-021-01289-z (PMC8632687; doi:10.1038/s41375-021-01289-z)
Supplement: Supplementary file 3 — Supplementary figure-2 [file 41375_2021_1289_MOESM3_ESM.pptx]

## Slide 1
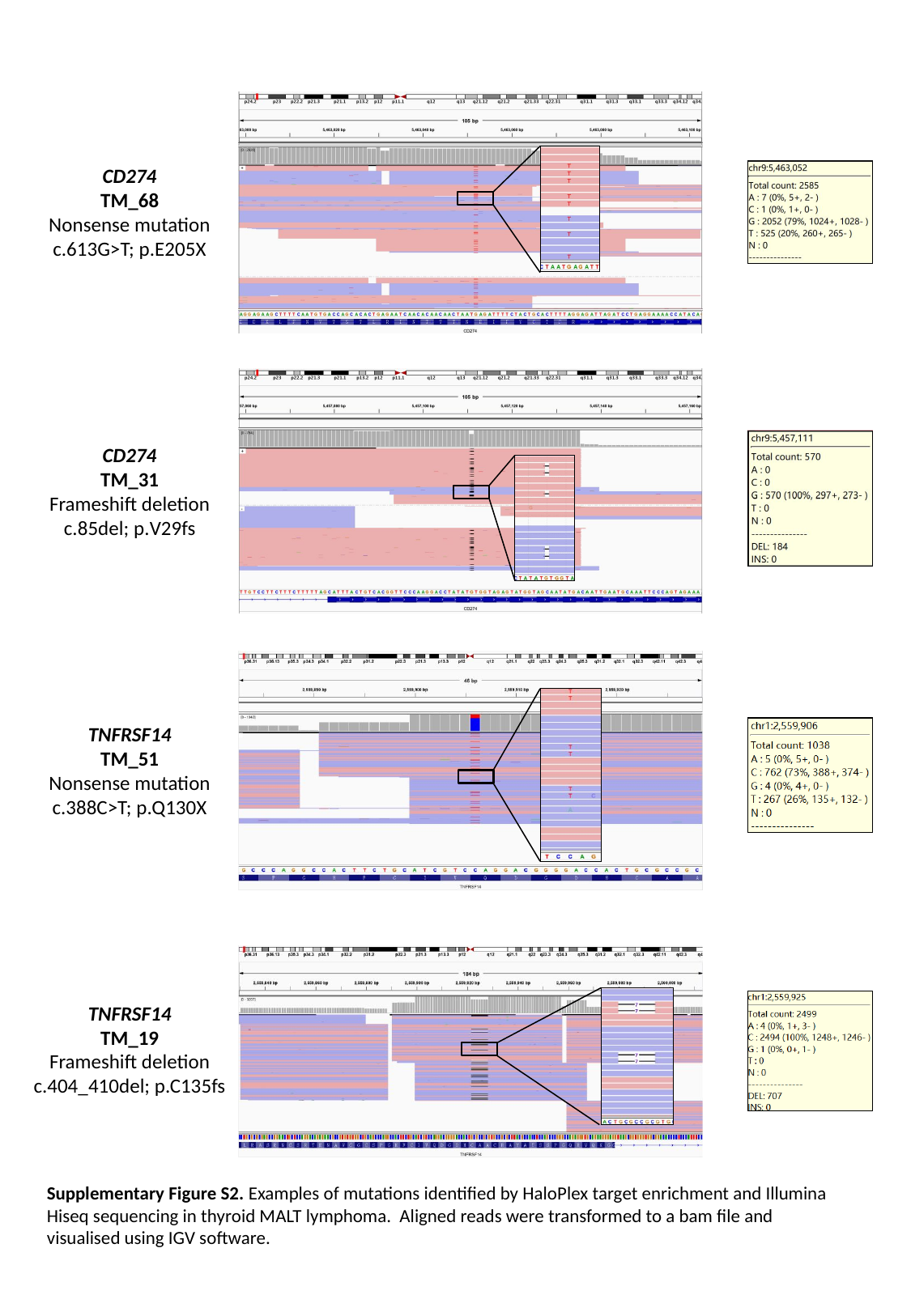

CD274
TM_68
Nonsense mutation
c.613G>T; p.E205X
CD274
TM_31
Frameshift deletion
c.85del; p.V29fs
TNFRSF14
TM_51
Nonsense mutation
c.388C>T; p.Q130X
TNFRSF14
TM_19
Frameshift deletion
c.404_410del; p.C135fs
Supplementary Figure S2. Examples of mutations identified by HaloPlex target enrichment and Illumina Hiseq sequencing in thyroid MALT lymphoma. Aligned reads were transformed to a bam file and visualised using IGV software.
