# Supplementary material for: Thyroid MALT lymphoma: self-harm to gain potential T-cell help
Source: Leukemia. 2021 May 21;35(12):3497–508. doi: 10.1038/s41375-021-01289-z (PMC8632687; doi:10.1038/s41375-021-01289-z)
Supplement: Supplementary file 4 — Supplementary figure-3 [file 41375_2021_1289_MOESM4_ESM.pptx]

## Slide 1
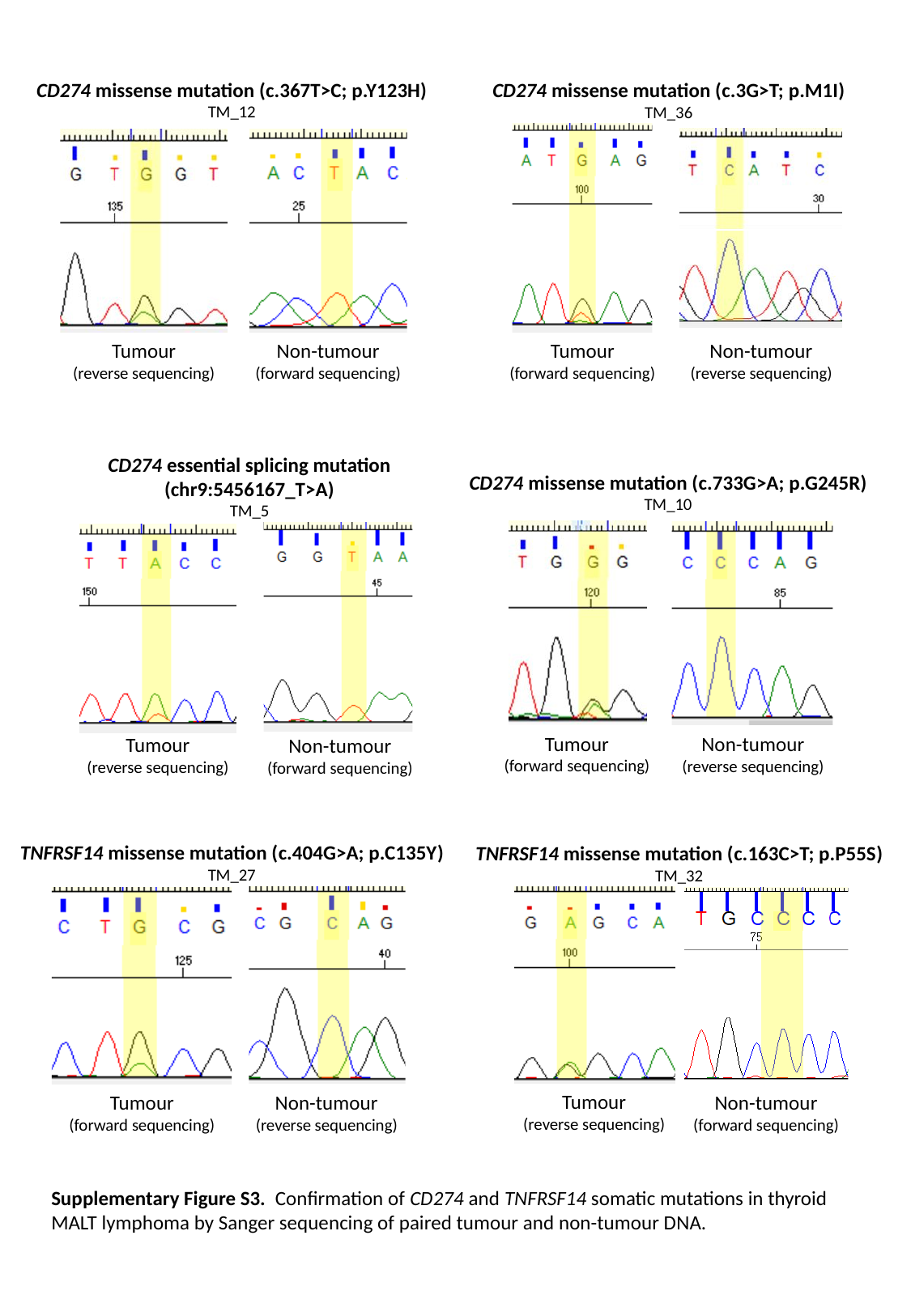

CD274 missense mutation (c.367T>C; p.Y123H)
TM_12
Tumour
(reverse sequencing)
Non-tumour
(forward sequencing)
CD274 missense mutation (c.3G>T; p.M1I)
TM_36
Tumour
(forward sequencing)
Non-tumour
(reverse sequencing)
CD274 essential splicing mutation (chr9:5456167_T>A)
TM_5
Non-tumour
(forward sequencing)
Tumour
(reverse sequencing)
CD274 missense mutation (c.733G>A; p.G245R)
TM_10
Tumour
(forward sequencing)
Non-tumour
(reverse sequencing)
TNFRSF14 missense mutation (c.404G>A; p.C135Y)
TM_27
Non-tumour
(reverse sequencing)
Tumour
(forward sequencing)
TNFRSF14 missense mutation (c.163C>T; p.P55S)
TM_32
Tumour
(reverse sequencing)
Non-tumour
(forward sequencing)
Supplementary Figure S3. Confirmation of CD274 and TNFRSF14 somatic mutations in thyroid MALT lymphoma by Sanger sequencing of paired tumour and non-tumour DNA.
