# Supplementary material for: Thyroid MALT lymphoma: self-harm to gain potential T-cell help
Source: Leukemia. 2021 May 21;35(12):3497–508. doi: 10.1038/s41375-021-01289-z (PMC8632687; doi:10.1038/s41375-021-01289-z)
Supplement: Supplementary file 5 — Supplementary figure-4 [file 41375_2021_1289_MOESM5_ESM.pptx]

## Slide 1
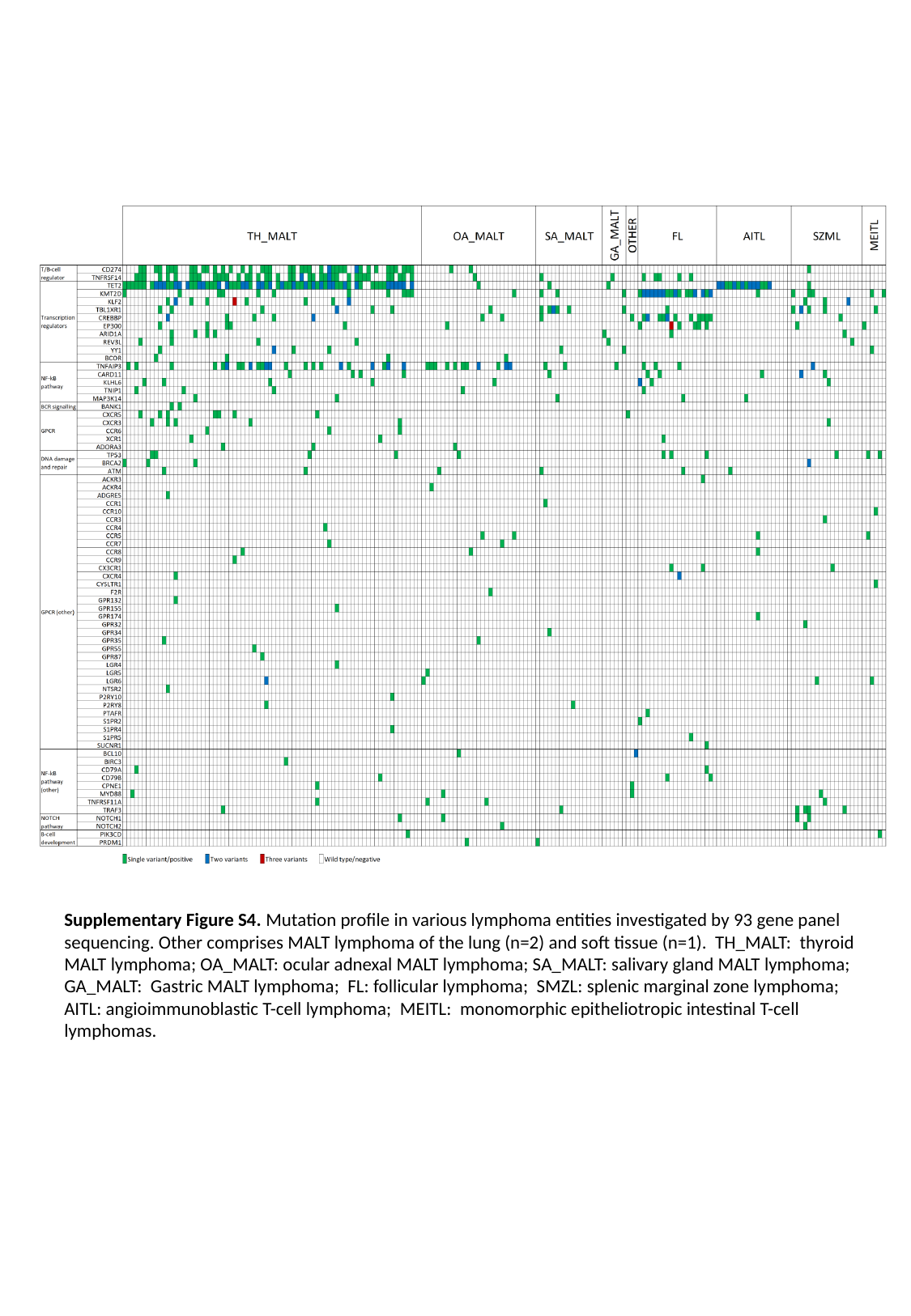

Supplementary Figure S4. Mutation profile in various lymphoma entities investigated by 93 gene panel sequencing. Other comprises MALT lymphoma of the lung (n=2) and soft tissue (n=1). TH_MALT: thyroid MALT lymphoma; OA_MALT: ocular adnexal MALT lymphoma; SA_MALT: salivary gland MALT lymphoma; GA_MALT: Gastric MALT lymphoma; FL: follicular lymphoma; SMZL: splenic marginal zone lymphoma; AITL: angioimmunoblastic T-cell lymphoma; MEITL: monomorphic epitheliotropic intestinal T-cell lymphomas.
