# Supplementary material for: Thyroid MALT lymphoma: self-harm to gain potential T-cell help
Source: Leukemia. 2021 May 21;35(12):3497–508. doi: 10.1038/s41375-021-01289-z (PMC8632687; doi:10.1038/s41375-021-01289-z)
Supplement: Supplementary file 6 — Supplementary figure-5 [file 41375_2021_1289_MOESM6_ESM.pptx]

## Slide 1
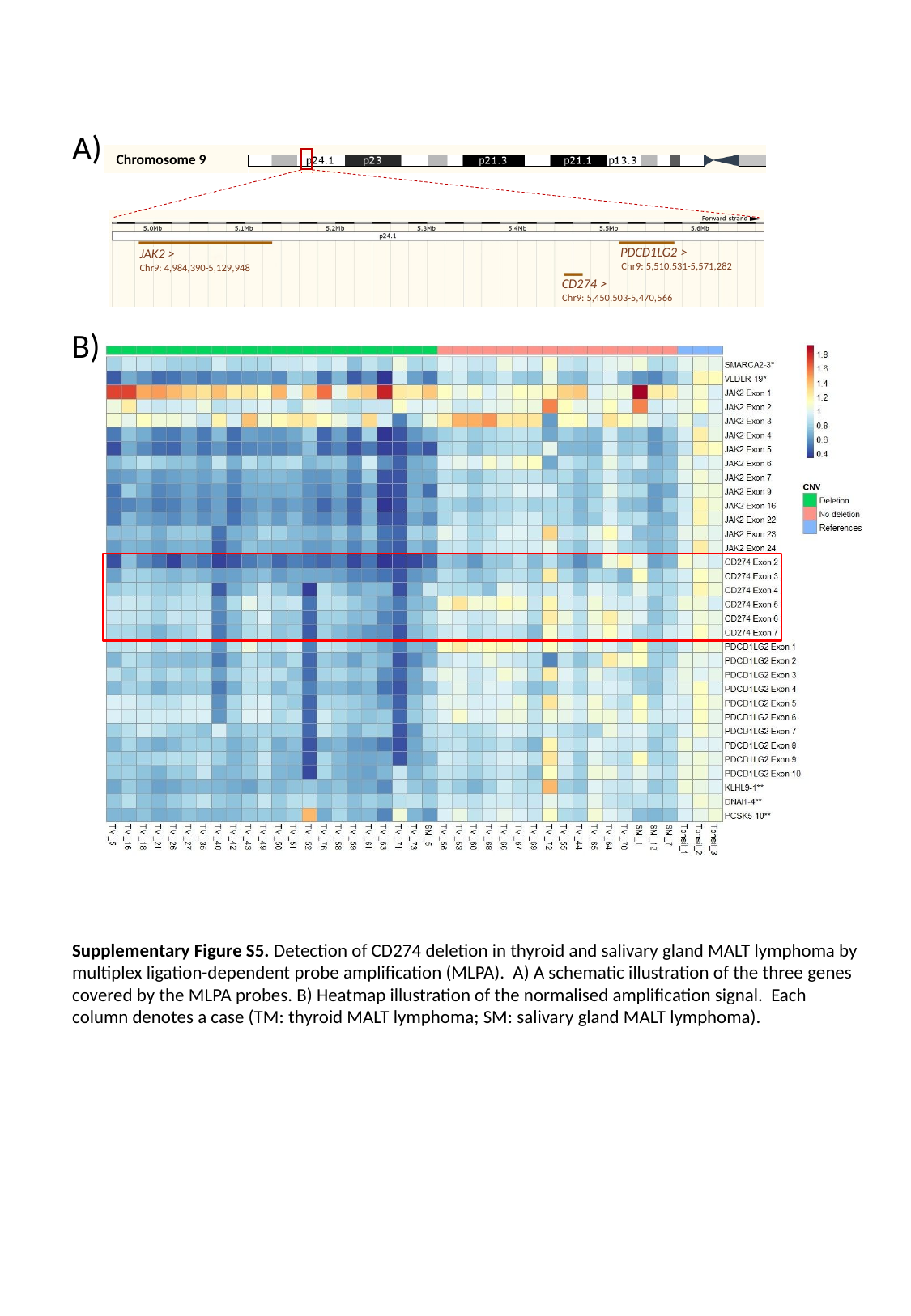

A)
B)
Supplementary Figure S5. Detection of CD274 deletion in thyroid and salivary gland MALT lymphoma by multiplex ligation-dependent probe amplification (MLPA). A) A schematic illustration of the three genes covered by the MLPA probes. B) Heatmap illustration of the normalised amplification signal. Each column denotes a case (TM: thyroid MALT lymphoma; SM: salivary gland MALT lymphoma).
