# Supplementary material for: Thyroid MALT lymphoma: self-harm to gain potential T-cell help
Source: Leukemia. 2021 May 21;35(12):3497–508. doi: 10.1038/s41375-021-01289-z (PMC8632687; doi:10.1038/s41375-021-01289-z)
Supplement: Supplementary file 7 — Supplementary figure-6 [file 41375_2021_1289_MOESM7_ESM.pptx]

## Slide 1
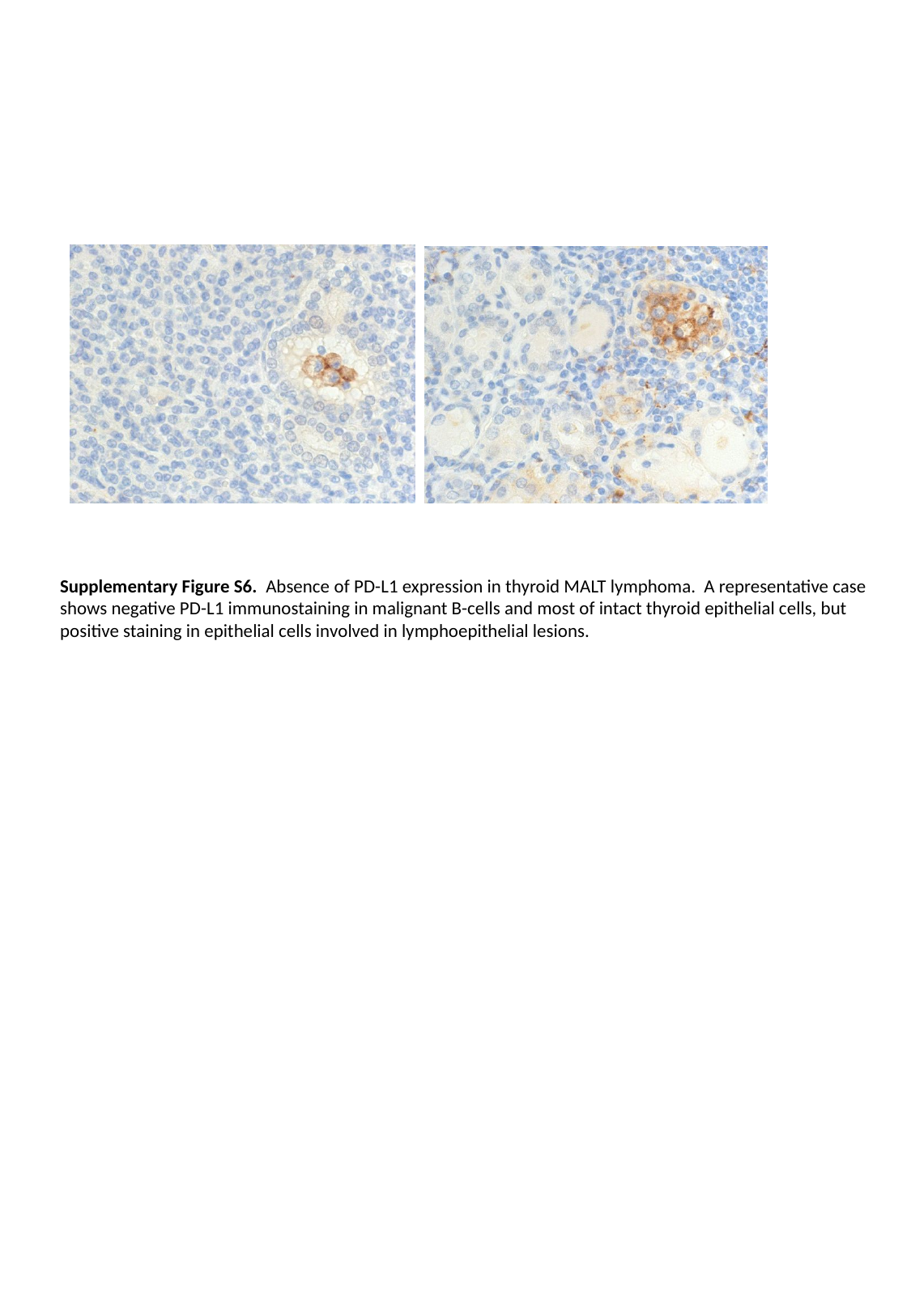

Supplementary Figure S6. Absence of PD-L1 expression in thyroid MALT lymphoma. A representative case shows negative PD-L1 immunostaining in malignant B-cells and most of intact thyroid epithelial cells, but positive staining in epithelial cells involved in lymphoepithelial lesions.
