# Supplementary material for: Thyroid MALT lymphoma: self-harm to gain potential T-cell help
Source: Leukemia. 2021 May 21;35(12):3497–508. doi: 10.1038/s41375-021-01289-z (PMC8632687; doi:10.1038/s41375-021-01289-z)
Supplement: Supplementary file 8 — Supplementary table S1 [file 41375_2021_1289_MOESM8_ESM.pdf]

Supplementary Table S1: Lymphoma entities and number of cases successfully investigated.

diagnosis		full name of the diagnosis	Number of cases	
MALT lymphoma	MALT_TH	Thyroid MALT lymphoma	76	131
	MALT_OA	Ocular adnexal MALT lymphoma	30	
	MALT_SA	Salivary gland MALT lymphoma	17	
	MALT_GA	Gastric MALT lymphoma	5	
	MALT_LU	Lung MALT lymphoma	2	
	MALT_SF	Soft tissue MALT lymphoma	1	
Other non-Hodgkin lymphoma	SMZL	Splenic marginal zone lymphoma	18	63
	FL	Follicular lymphoma	20	
	AITL	Angioimmunoblastic T-cell lymphoma	19	
	MEITL	Monomorphic epitheliotropic intestinal T cell lymphoma	6	
**Total number**			**194**	
