# Supplementary material for: Thyroid MALT lymphoma: self-harm to gain potential T-cell help
Source: Leukemia. 2021 May 21;35(12):3497–508. doi: 10.1038/s41375-021-01289-z (PMC8632687; doi:10.1038/s41375-021-01289-z)
Supplement: Supplementary file 9 — Supplementary table S2 [file 41375_2021_1289_MOESM9_ESM.pdf]

Supplementary Table S2: List of 93-genes investigated by targeted sequencing.

CANDIDATE GENE		MALT LYMPHOMA RELATED		GPCR GENE			
DNA damage and repair	REV3L	Apoptosis/DNA damage	ATM	Adenosine receptor	ADORA2B	Class A orphan receptor	GPR174
	BRCA2		TP53		ADORA3		GPR18
Transcriptional regulato	BCOR	B cell development	KLHL6	Adhesion receptor	ADGRE5		GPR183
NF-κB pathway	CPNE1		PIK3CD		ADGRG3		GPR31
BCR signaling	BANK1		PRDM1		GPR98		GPR32
		Epigenetic/Translational regulator	ARID1A	Adrenoceptor	ADRB2		GPR34
			CREBBP	Chemokine receptor	ACKR3		GPR35
			EP300		ACKR4		GPR55
			KLF2		CCR1		GPR84
			KMT2D		CCR10		GPR87
			TBL1XR1		CCR2		LGR4
			TET2		CCR3		LGR5
			YY1		CCR4		LGR6
		T/B-cell regulator	CD274		CCR5		P2RY10
OTHER REGION			TNFRSF14		CCR6		P2RY8
chr19:16434500-16435725	KLF2_5'UTR	NF-κB pathway	BCL10		CCR7	Class A receptor	PTAFR
chr19:16435810-16436026	KLF2_intron1/2		BIRC3		CCR8		SUCNR1
chr6:138180000-138196500	TNFAIP3_5'UTR		CARD11		CCR9	Estrogen receptor	GPER1
chr9:139388875-139390513	NOTCH1_3'UTR		CD79A		CCRL2	Frizzled receptor	SMO
chr1:120454166-120457919	NOTCH2_3'UTR		CD79B		CX3CR1	Leukotriene receptor	CYSLTR1
			MAP3K14		CXCR1	LPA receptor	LPAR5
			MYD88		CXCR2	Neurotensin receptor	NTSR2
			TNFAIP3		CXCR3	Prostaglandin receptor	PTGER4
			TNFRSF11A		CXCR4	rotease-activated recepto	F2R
			TNIP1		CXCR5	S1P receptor	S1PR1
			TRAF3		CXCR6		S1PR2
		NOTCH pathway	NOTCH1		XCR1		S1PR3
			NOTCH2	Class A orphan receptor	GPR132		S1PR4
					GPR15		S1PR5
					GPR171	/	GPR155
5 GENES		28 GENES		60 GENES			
IN TOTAL: 93 GENES		COVERAGE: 99.45 %		TARGET SIZE: 279kbp		SEQUENCEABLE: 549kbp	
