# Supplementary material for: Thyroid MALT lymphoma: self-harm to gain potential T-cell help
Source: Leukemia. 2021 May 21;35(12):3497–508. doi: 10.1038/s41375-021-01289-z (PMC8632687; doi:10.1038/s41375-021-01289-z)
Supplement: Supplementary file 10 — Supplementary table S3 [file 41375_2021_1289_MOESM10_ESM.pdf]

Supplementary Table S3: Primers used for PCR and Sanger sequencing of the CD274 and TNFRSF14 genes.

gene	exon	forward/reverse primer	sequence (5'-3')	amplicon size (bp)	PCR condition	target position (hg38)
CD274	CD274-exon2-1	forward	CAGTTAGAACCACCAAGTCCCA	162	60°C	chr9:5456013-5456174
		reverse	GTGTCTTACCGTTCAGCAAATG			
	CD274-exon2-3	forward	GGCATTCCAGAAAGATGAGGA	216	60°C	chr9:5456100-5456315
		reverse	AGAAGACTTTGCCATTGTGT			
	CD274-exon3-1	forward	TCGAGGAATTTTCCCTTTTCTGA	159	60°C	chr9:5457033-5457191
		reverse	GACAATTAGTGCAGCCAGGTC			
	CD274-exon3-2	forward	GTAGCTACAGACAGAGGGCC	150	60°C	chr9:5457262-5457411
		reverse	TCACAGTAATTCGCTTGTAGTCG			
	CD274-exon3-3	forward	ACAATTAGACCTGGCTGCACT	83	60°C	chr9:5457164-5457246
		reverse	GGTCTTCCTCTCCATGCACAA			
	CD274-exon3-4	forward	GTGCATGGAGAGGAAGACCTG	149	60°C	chr9:5457228-5457376
		reverse	CTGATCATGCAGCGGTACAC			
	CD274-exon3-6	forward	CCGCTGCATGATCAGCTATG	173	60°C	chr9:5457362-5457534
		reverse	CAGACTATGCTGTTCTGTTTGAA			
	CD274-exon4-1	forward	CAGCAAAAGCCCTGACTTCT	203	60°C	chr9:5462797-5462999
		reverse	TCTCTCTTGGAATTGGTGGTGG			
	CD274-exon4-2	forward	AGACCACCACCACCAATTCC	200	60°C	-
		reverse	GCTAGGGGACAGTGTTAGACA			
	CD274-exon5	forward	ATGACCATTCTCCTGACCCC	239	60°C	chr9:5465423-5465661
		reverse	ATTCTCACCCCTTCCTCACC			
TNFRSF14	TNFRSF14-exon1	forward	CAATGGCGCTGAGTTCCTCT	142	60°C	chr1:2556591-2556732
		reverse	AGCCTCAAGACGTCGGTTTT			
	TNFRSF14-exon2	forward	CATCTCCCAATGCCTGTCCT	230	60°C	chr1:2557695-2557924
		reverse	GGCAGAGAACACAGGGGT			
	TNFRSF14-exon3	forward	TCTGGGCGGCAGGTTATCGT	213	60°C	chr1:2558331-2558543
		reverse	TGGAGAGAGGGTGCAGGGTG			
	TNFRSF14-exon4	forward	CTCTCAGCCCCTCCTCTTG	188	60°C	chr1:2559796-2559983
		reverse	CTTACCTCCCTTCTGCACCC			
	TNFRSF14-exon5	forward	CTCTCTTCTCAGGCACCGAG	175	60°C	chr1:2560612-2560786
		reverse	TCATCTCCCAGGGAAGCAAC			
	TNFRSF14-exon6	forward	CTCCCTGAGGCTGAGTGAAC	213	60°C	chr1:2561604-2561816
		reverse	CCCCTTGGCTTTCTTCTTTTCA			

PCR cycle		
95°C	10min	X1
96°C	30s	X40
60°C	30s	
72°C	30s	
72°C	10min	X1

The CS1 tag (ACACTGACGACATGGTTCTACA) and the CS2 tag (TACGGTAGCAGAGACTTGGTCT) were added to the 5' of the forward and reverse primers respectively, and used for Sanger sequencing.
