# Supplementary material for: Thyroid MALT lymphoma: self-harm to gain potential T-cell help
Source: Leukemia. 2021 May 21;35(12):3497–508. doi: 10.1038/s41375-021-01289-z (PMC8632687; doi:10.1038/s41375-021-01289-z)
Supplement: Supplementary file 11 — Supplementary table S4 [file 41375_2021_1289_MOESM11_ESM.pdf]

Supplementary Table S4: Antibodies and reagens used for immunoflourescent or immunohistochemical staining

**Antibodies used for Immunoflourscent staining**	**Antibody type**	**Clone name**	**Dilution**	**Source**	**Opal fluorophore and dilution**
**Primary antibodies**					
Anti-CD4	Mouse Monoclonal	4B12	1:50	Leica Microsystems Ltd., Newcastle-upon-Tyne, UK	Opal-540 (1:100)
Anti-CD8	Mouse Monoclonal	4B11	1:100	Leica Microsystems Ltd., Newcastle-upon-Tyne, UK	Opal-620 (1:150)
Anti-CD20	Mouse Monoclonal	L26	1:100	Leica Microsystems Ltd., Newcastle-upon-Tyne, UK	Opal-690 (1:50)
Anti-CD69	Rabbit Monoclonal	EPR21814	1:500	Abcam Plc.,Cambridge, UK	Opal-650 (1:100)
Anti-Ki67	Mouse Monoclonal	MIB-1	1:100	Agilent Technologies LDA UK Ltd.Cheshire ,UK	Opal-620 (1: 150)
Anti-PD1	Mouse Monoclonal	NAT 105/E3	1:2	Kindly gifted by Dr G Roncador, CNIO, Madrid (Spain)	Opal-520 (1:50)
Anti-PD-L1	Mouse Monoclonal	22C3	ready to use	Agilent Technologies LDA UK Ltd.Cheshire ,UK	Opal-570 (1: 50)

Secondary antibodies

HRP-conjugated secondary polymer	DS9800		Leica Microsystems Ltd., Newcastle-upon-Tyne, UK	

**Antibodies used for Immunohistochemistry**					
Anti-PD-L1	Rabbit monoclonal	E1L3N	1/200	Cell Signalling	
