# Supplementary material for: Thyroid MALT lymphoma: self-harm to gain potential T-cell help
Source: Leukemia. 2021 May 21;35(12):3497–508. doi: 10.1038/s41375-021-01289-z (PMC8632687; doi:10.1038/s41375-021-01289-z)
Supplement: Supplementary file 13 — Supplementary table S6 [file 41375_2021_1289_MOESM13_ESM.pdf]

Supplementary Table S6. Clinical features and correlations with *CD274 /TNFRSF14 /TET2* changes and histological features.

				Genetic alteration															Histological features**								
				CD274			TNFRSF14			TET2			CD274&TNFRSF14			CD274&TNFRSF14&TET2			plasmacytoid differentiation			transformed blasts			follicular colonization		
Clinical features			n=76	n=52	n=24		n=40	n=36		n=65	n=11		n=34	n=42		n=32	n=44		n=43 (score=0)	n=27 (score≥1)		n=39 (score=1)	n=31 (score ≥2)		n=32 (score=0)	n=38 (score ≥1)	
variable		level	total	mutated/CNV del	WT	*p* value	mutated	WT	*p* value	mutated	WT	*p* value	mutated/CNV del	WT	*p* value	mutated/CNV del	WT	*p* value	0	1-2	*p* value	1	2-3	*p* value	0	1-2	*p* value
basic features	age	<60	10	6	4	**0.0473**	6	4	0.8988	8	2	0.3121	5	5	0.6834	4	6	0.7046	7	2	0.317	4	5	0.167	4	5	0.464
		≥60, <70	22	10	12		11	11		18	4		8	14		8	14		11	9		12	8		11	9	
		≥70, <80	27	22	5		15	12		26	1		14	13		14	13		17	9		17	9		13	13	
		≥80	13	10	3		6	7		12	1		5	8		5	8		8	5		4	9		3	10	
	gender	male	4	4	0	0.2939	2	2	1.0000	4	0	1.0000	2	2	1.0000	2	2	1.0000	2	2	0.621	34	30	0.62	2	2	1
		female	68	44	24		36	32		60	8		30	38		29	39		41	23		3	1		29	35	
	stage	IE	43	28	15	0.6838	24	19	0.8912	39	4	0.2050	19	24	0.9775	19	24	0.9720	26	15	0.406	26	15	0.157	19	22	0.375
		IIE	26	19	7		13	13		23	3		12	14		11	15		15	9		9	15		9	15	
		IIIE	2	1	1		1	1		1	1		1	1		1	1		1	1		1	1		2	0	
Hashimoto's thyroiditis (HT)	Hashimoto's thyroiditis	no	10	3	7	**0.0128**	2	8	**0.0390**	9	1	1.0000	1	9	**0.0354**	1	9	**0.0360**	6	4	1	8	2	0.097	5	5	1
		yes	62	45	17		36	26		55	7		31	31		30	32		37	21		29	29		26	32	
	average duration of HT to TH MALToma (year)	all cases	5.4y	6.5y	2.3y	0.0821	6.1y	4.4y	0.4427	5.5y	4.5y	0.7833	6.8y	3.9y	0.1918	7.0y	3.8y	0.1432	4.2y	6.4y	0.35	4.9y	5.2y	0.9	5.2y	4.9y	0.89
		exclude "simutaneous HT dia	11.3y	11.6y	9.04y	0.6316	11.3y	11.1y	0.9609	11.4y	10.1y	0.8275	11.6y	10.8y	0.8240	11.6y	10.8y	0.8240	9.6y	12.0y	0.53	9.9y	11.4	0.69	9.5y	11.8y	0.53
laboratory results	TgAb	normal	14	7	7	0.2059	5	9	0.2332	12	2	0.6483	4	10	0.2374	4	10	0.2488	10	4	0.548	7	7	0.769	8	6	0.378
		high	58	41	17		33	25		52	6		28	30		27	31		33	21		30	24		23	31	
	TPOAb	normal	31	19	12	0.6151	15	16	0.4742	29	2	0.2810	12	19	0.4661	12	19	0.6258	19	12	0.802	20	11	0.136	17	14	0.139
		high	38	26	12		22	16		32	6		19	19		18	20		22	12		15	19		12	22	
	FT3	normal	71	48	23	0.3333	37	34	1.0000	64	7	0.1111	32	39	1.0000	31	40	1.0000	43	25	/	37	31	/	31	37	/
		abnormal	1	0	1		1	0		0	1		0	1		0	1		0	0		0	0		0	0	
	FT4	normal	66	44	22	1.0000	36	30	0.4119	59	7	0.5201	31	35	0.2167	30	36	0.2268	41	22	0.349	35	28	0.653	29	34	1
		abonormal	6	4	2		2	4		5	1		1	5		1	5		2	3		2	3		2	3	
	TSH	normal	56	39	17	0.3730	32	24	0.2560	51	5	0.3645	27	29	0.2663	26	30	0.3925	34	20	1	33	21	**0.038**	27	27	0.229
		high	16	9	7		6	10		13	3		5	11		5	11		9	5		4	10		4	10	
	LDH	normal	60	41	19	0.5181	32	28	1.0000	54	6	0.6128	29	31	0.2051	28	32	0.2119	36	22	0.735	35	23	**0.035**	29	29	0.097
		high	12	7	5		6	6		10	2		3	9		3	9		7	3		2	8		2	8	
	ESR	normal	51	27	24	**0.0001**	25	26	0.4404	44	7	0.4267	19	32	0.1119	18	33	0.0678	31	16	0.782	26	21	0.791	22	25	1
		high	20	20	0		12	8		19	1		12	8		12	8		12	8		10	10		9	11	
	sIL2R	normal	47	30	17	0.4265	24	23	1.0000	43	4	0.4296	20	27	0.6187	19	28	0.4606	32	13	0.109	25	20	1	25	20	**0.038**
		high	24	18	6		13	11		20	4		12	12		12	12		11	11		12	10		6	16	
drug taking	levothyroxine/iodine taking	no	50	33	17	1.0000	24	26	0.3063	45	5	0.6933	19	31	0.1251	18	32	0.0778	30	17	1	27	20	0.599	23	24	0.442
		yes	22	15	7		14	8		19	3		13	9		13	9		13	8		10	11		8	13	
survival*	follow-up	stay remission	67	45	22	/	36	31	/	59	8	/	30	37	/	29	38	/	42	21	/	33	30	/	28	35	/
		progressed/alive	2	2	0		2	0		2	0		2	0		2	0		0	2		2	0		0	2	
		progressed/died	1	0	1		0	1		1	0		0	1		0	1		0	1		1	0		1	0	
		died due to other reason	2	1	1		0	2		2	0		0	2		0	2		1	1		1	1		2	0	

*The majority (n=62) of cases were treated by local radiotherapy alone (n=49) or thyroidectomy plus local radiotherapy (n=13), with the remaining cases treated by thyroidectomy or chemotherapy with or without Rituximab. The average follow up of this cohort is 46 months (range 0.4-107 months).

**Plasmacytoid differentiation: 0: no plasmacytoid differentiation; 1: with focal or scatted plasmacytoid differentiation; 2: diffuse plasmacytoid differentiation or ≥ 50%

Transformed blasts: 1: 0-5 large cells(resembling centroblasts or immunoblasts) per HPF; 2: 6-15 large cells per HPF; 3: ＞15 large cells per HPF

Follicular colonization: 0: no obvious follicular colonization; 1: with follicular colonization, the frequency ≤ 5/10 ; 2: with with follicular colonization, the frequency ＞ 5/10
