# Supplementary material for: Thyroid MALT lymphoma: self-harm to gain potential T-cell help
Source: Leukemia. 2021 May 21;35(12):3497–508. doi: 10.1038/s41375-021-01289-z (PMC8632687; doi:10.1038/s41375-021-01289-z)
Supplement: Supplementary file 12 — Supplementary table S5 [file 41375_2021_1289_MOESM12_ESM.pdf]

Supplementary Table S5: Variants detected by 93-gene panel sequencing.

Diagnosis	Sample.ID	Sample.ID-2	Variant	Freque	Gene	AD.Ref	AD.Alt1	AD.Alt2	DP	VAF.Ref	VAF.Alt1	VAF.Alt2	Func.refGe	func	AAChange.	Reproducil	X1000g201	avsnp144
AITL	AITL_1	AITL_1808_4006	chr4:105275143_C>T	1	TET2	1054	251	NA	1307	81%	19%	NA	exonic	stopgain	TET2:NM_	NA	.	.
AITL	AITL_1	AITL_1808_4006	chr4:105275418_C>-	1	TET2	357	82	NA	439	81%	19%	NA	exonic	frameshift	TET2:NM_	NA	.	.
AITL	AITL_2	AITL_1809_4006	chr4:105241373_TACCCATCT>-	1	TET2	895	55	NA	950	94%	6%	NA	exonic	nonframes	TET2:NM_	NA	.	.
AITL	AITL_2	AITL_1809_4006	chr4:105275149_C>T	1	TET2	1836	171	NA	2010	91%	9%	NA	exonic	stopgain	TET2:NM_	NA	.	.
AITL	AITL_3	AITL_1809_6295B	chr4:105276221_A>G	1	TET2	4597	612	NA	5215	88%	12%	NA	exonic	nonsynony	TET2:NM_	NA	.	.
AITL	AITL_4	AITL_CAM_01	chr11:108330215_TACACAG>-	1	ATM	878	217	NA	1095	80%	20%	NA	exonic	frameshift	ATM:NM_	NA	.	.
AITL	AITL_4	AITL_CAM_01	chr4:105272735_C>T	1	TET2	256	158	NA	414	62%	38%	NA	exonic	stopgain	TET2:NM_	NA	.	.
AITL	AITL_5	AITL_CAM_03	chr4:105269644_T>C	1	TET2	387	118	NA	507	76%	23%	NA	exonic	nonsynony	TET2:NM_	NA	.	.
AITL	AITL_5	AITL_CAM_03	chr4:105275987_A>-	1	TET2	2025	812	NA	2837	71%	29%	NA	exonic	frameshift	TET2:NM_	NA	.	.
AITL	AITL_6	AITL_CAM_06	chr4:105276211_T>C	1	TET2	531	45	NA	579	92%	8%	NA	exonic	nonsynony	TET2:NM_	NA	.	.
AITL	AITL_7	AITL_CAM_10	chr4:105235901_C>-	1	TET2	1175	299	NA	1474	80%	20%	NA	exonic	frameshift	TET2:NM_	NA	.	.
AITL	AITL_7	AITL_CAM_10	chr4:105237352_->T	1	TET2	1345	218	NA	1563	86%	14%	NA	exonic;spli	frameshift	TET2:NM_	NA	.	.
AITL	AITL_8	AITL_CAM_12	chr4:105259628_C>A	1	TET2	603	75	NA	682	88%	11%	NA	exonic	stopgain	TET2:NM_	NA	.	.
AITL	AITL_8	AITL_CAM_12	chr17:45289255_C>T	1	MAP3K14	106	21	NA	135	79%	16%	NA	exonic	nonsynony	MAP3K14:	NA	.	rs3690041
AITL	AITL_9	AITL_CAM_19	chr4:105236247_->A	1	TET2	374	109	NA	483	77%	23%	NA	exonic	frameshift	TET2:NM_	NA	.	.
AITL	AITL_9	AITL_CAM_19	chr4:105272591_C>T	1	TET2	1160	256	NA	1423	82%	18%	NA	exonic	stopgain	TET2:NM_	NA	.	.
AITL	AITL_10	AITL_CAM_37	chr4:105242841_C>T	1	TET2	428	89	NA	521	82%	17%	NA	exonic	stopgain	TET2:NM_	NA	.	rs7705545
AITL	AITL_10	AITL_CAM_37	chr4:105276137_C>T	1	TET2	5766	1047	NA	6825	84%	15%	NA	exonic	nonsynony	TET2:NM_	NA	.	.
AITL	AITL_11	AITL_CAM_39	chr12:49029178_C>A	1	KMT2D	2854	400	NA	3259	88%	12%	NA	exonic	stopgain	KMT2D:NM	NA	.	.
AITL	AITL_11	AITL_CAM_39	chrX:79171849_C>G	1	GPR174	1139	488	NA	1635	70%	30%	NA	exonic	nonsynony	GPR174:NM	NA	.	.
AITL	AITL_11	AITL_CAM_39	chr3:46373537_T>C	1	CCR5	1859	1179	NA	3046	61%	39%	NA	exonic	nonsynony	CCR5:NM_	NA	.	.
AITL	AITL_11	AITL_CAM_39	chr3:39332858_C>T	1	CCR8	1104	1229	NA	2344	47%	52%	NA	exonic	nonsynony	CCR8:NM_	NA	.	.
AITL	AITL_11	AITL_CAM_39	chr4:105236761_C>-	1	TET2	1105	472	NA	1577	70%	30%	NA	exonic	frameshift	TET2:NM_	NA	.	.
AITL	AITL_11	AITL_CAM_39	chr4:105272852_A>T	1	TET2	1612	459	NA	2084	77%	22%	NA	exonic	stopgain	TET2:NM_	NA	.	.
AITL	AITL_12	AITL_CAM_42	chr7:2915371_A>C	1	CARD11	445	109	NA	568	78%	19%	NA	exonic	nonsynony	CARD11:NM	NA	.	.
AITL	AITL_12	AITL_CAM_42	chr4:105236839_A>-	1	TET2	1241	907	NA	2148	58%	42%	NA	exonic	frameshift	TET2:NM_	NA	.	.
AITL	AITL_13	AITL_CAM_43	chr4:105235738_A>-	1	TET2	95	41	NA	136	70%	30%	NA	exonic	frameshift	TET2:NM_	NA	.	.
AITL	AITL_14	AITL_CAM_44	chr4:105237059_->TTATTTGACC	1	TET2	2250	266	NA	2516	89%	11%	NA	exonic	frameshift	TET2:NM_	NA	.	.
AITL	AITL_14	AITL_CAM_44	chr4:105243610_T>A	1	TET2	2670	275	NA	2953	90%	9%	NA	exonic	stopgain	TET2:NM_	NA	.	.
FL	FL_1	FL_1152	chr22:41129965_T>C	2	EP300	263	96	NA	359	73%	27%	NA	exonic	nonsynony	EP300:NM	NA	.	.
FL	FL_1	FL_1152	chr3:183555418_T>A	1	KLHL6	1653	534	NA	2195	75%	24%	NA	exonic	nonsynony	KLHL6:NM	NA	.	.
FL	FL_1	FL_1152	chr3:183555493_A>G	1	KLHL6	629	117	NA	750	84%	16%	NA	exonic	nonsynony	KLHL6:NM	NA	.	.
FL	FL_1	FL_1152	chr12:49052126_G>-	1	KMT2D	1018	1476	NA	2494	41%	59%	NA	exonic	frameshift	KMT2D:NM	NA	.	.
FL	FL_1	FL_1152	chr19:10224904_A>G	1	S1PR2	1633	358	NA	1993	82%	18%	NA	exonic	nonsynony	S1PR2:NM	NA	.	.
FL	FL_2	FL_1154	chr5:151035689_C>G	1	TNIP1	711	739	NA	1455	49%	51%	NA	exonic	nonsynony	TNIP1:NM_	NA	.	.
FL	FL_2	FL_1154	chr1:2561732_G>A	1	TNFRSF14	334	823	NA	1158	29%	71%	NA	exonic	stopgain	TNFRSF14:	NA	.	.
FL	FL_2	FL_1154	chr12:49046369_G>A	1	KMT2D	809	1097	NA	1913	42%	57%	NA	exonic	stopgain	KMT2D:NM	NA	.	.
FL	FL_2	FL_1154	chr12:49054753_T>G	1	KMT2D	2206	989	NA	3203	69%	31%	NA	splicing	.	.	NA	.	.
FL	FL_2	FL_1154	chr16:3758867_->G	1	CREBBP	1609	465	NA	2074	78%	22%	NA	exonic	frameshift	CREBBP:NM	NA	.	.
FL	FL_2	FL_1154	chr6:137871313_T>-	1	TNFAIP3	166	606	NA	772	22%	78%	NA	exonic	frameshift	TNFAIP3:N	NA	.	.
FL	FL_3	FL_1165	chr7:2939867_T>G	1	CARD11	1276	247	NA	1524	84%	16%	NA	exonic	nonsynony	CARD11:NM	NA	.	.
FL	FL_3	FL_1165	chr12:49029122_C>T	1	KMT2D	689	213	NA	910	76%	23%	NA	exonic	stopgain	KMT2D:NM	NA	.	.
FL	FL_3	FL_1165	chr12:49034282_G>A	1	KMT2D	1036	279	NA	1320	78%	21%	NA	exonic	stopgain	KMT2D:NM	NA	.	.
FL	FL_3	FL_1165	chr16:3738617_G>A	1	CREBBP	1525	472	NA	2002	76%	24%	NA	exonic	nonsynony	CREBBP:NM	NA	.	rs3981241
FL	FL_3	FL_1165	chr16:3770850_GGCATGC>-	1	CREBBP	1218	256	NA	1474	83%	17%	NA	exonic	frameshift	CREBBP:NM	NA	.	.
FL	FL_3	FL_1165	chr1:28150261_G>A	1	PTAFR	1296	472	NA	1775	73%	27%	NA	exonic	nonsynony	PTAFR:NM	NA	.	.
FL	FL_4	FL_1180	chr3:183555367_T>A	1	KLHL6	1471	659	NA	2137	69%	31%	NA	exonic	nonsynony	KLHL6:NM	NA	.	.
FL	FL_4	FL_1180	chr12:49026277_C>A	1	KMT2D	1280	1299	NA	2584	50%	50%	NA	exonic	nonsynony	KMT2D:NM	NA	.	.
FL	FL_4	FL_1180	chr12:49048065_AT>-	1	KMT2D	3096	758	NA	3854	80%	20%	NA	exonic	frameshift	KMT2D:NM	NA	.	rs3981237
FL	FL_5	FL_1182	chr1:2560657_->C	2	TNFRSF14	1151	734	NA	1885	61%	39%	NA	exonic	frameshift	TNFRSF14:	NA	.	rs7720884
FL	FL_5	FL_1182	chr12:49037303_G>-	1	KMT2D	1656	797	NA	2453	68%	32%	NA	exonic	frameshift	KMT2D:NM	NA	.	.
FL	FL_5	FL_1182	chr12:49044423_T>-	1	KMT2D	1019	569	NA	1588	64%	36%	NA	exonic	frameshift	KMT2D:NM	NA	.	.
FL	FL_5	FL_1182	chr6:137880175_G>T	1	TNFAIP3	826	682	NA	1511	55%	45%	NA	exonic	stopgain	TNFAIP3:N	NA	.	.
FL	FL_6	FL_1183	chr7:2937980_T>A	1	CARD11	1692	154	NA	1856	91%	8%	NA	exonic	nonsynony	CARD11:NM	NA	.	.
FL	FL_6	FL_1183	chr1:2559939_->GA	1	TNFRSF14	722	282	NA	1004	72%	28%	NA	exonic	stopgain	TNFRSF14:	NA	.	.
FL	FL_6	FL_1183	chr12:49027000_C>T	1	KMT2D	872	820	NA	1698	51%	48%	NA	exonic	nonsynony	KMT2D:NM	NA	.	rs7669186
FL	FL_6	FL_1183	chr12:49041175_A>-	2	KMT2D	959	98	NA	1057	91%	9%	NA	exonic	frameshift	KMT2D:NM	NA	.	rs3981237
FL	FL_6	FL_1183	chr16:3736703_A>G	1	CREBBP	2657	286	NA	2969	89%	10%	NA	exonic	nonsynony	CREBBP:NM	NA	.	.
FL	FL_7	FL_1627	chr12:49041175_A>-	2	KMT2D	917	162	NA	1079	85%	15%	NA	exonic	frameshift	KMT2D:NM	NA	.	rs3981237
FL	FL_7	FL_1627	chr12:49046756_C>T	1	KMT2D	1889	283	NA	2174	87%	13%	NA	exonic	nonsynony	KMT2D:NM	NA	.	.
FL	FL_7	FL_1627	chr16:3736794_C>G	1	CREBBP	3510	444	NA	3962	89%	11%	NA	exonic	nonsynony	CREBBP:NM	NA	.	.
FL	FL_7	FL_1627	chr17:7674217_C>A	1	TP53	1060	89	NA	1149	92%	8%	NA	exonic	nonsynony	TP53:NM_	NA	.	.
FL	FL_7	FL_1627	chr3:46021610_A>G	1	XCR1	904	744	NA	1655	55%	45%	NA	exonic	nonsynony	XCR1:NM_	NA	.	.
FL	FL_8	FL_1638	chr12:49034478_T>C	1	KMT2D	1344	507	NA	1854	72%	27%	NA	splicing	.	.	NA	.	.
FL	FL_8	FL_1638	chr16:3736765_T>G	1	CREBBP	2250	1149	NA	3402	66%	34%	NA	exonic	nonsynony	CREBBP:NM	NA	.	.
FL	FL_8	FL_1638	chr16:3739596_C>T	1	CREBBP	2243	1097	NA	3350	67%	33%	NA	exonic	nonsynony	CREBBP:NM	NA	.	rs2006165
FL	FL_8	FL_1638	chr3:177038396_A>C	1	TBL1XR1	1186	618	NA	1805	66%	34%	NA	exonic	nonsynony	TBL1XR1:N	NA	.	.
FL	FL_8	FL_1638	chr17:63929304_->A	1	CD79B	2129	910	NA	3039	70%	30%	NA	exonic	frameshift	CD79B:NM	NA	.	.
FL	FL_9	FL_1639	chr12:49038850_GA>-	1	KMT2D	1012	654	NA	1666	61%	39%	NA	exonic	frameshift	KMT2D:NM	NA	.	.
FL	FL_9	FL_1639	chr1:26731200_C>T	1	ARID1A	1783	1182	NA	2974	60%	40%	NA	exonic	stopgain	ARID1A:NM	NA	.	.
FL	FL_9	FL_1639	chr3:39266311_C>T	1	CX3CR1	507	506	NA	1014	50%	50%	NA	exonic	nonsynony	CX3CR1:NM	NA	.	rs7660760
FL	FL_9	FL_1639	chr22:41170489_T>C	1	EP300	759	410	NA	1172	65%	35%	NA	exonic	nonsynony	EP300:NM	NA	.	.
FL	FL_9	FL_1639	chr22:41170566_T>G	1	EP300	1051	583	NA	1648	64%	35%	NA	exonic	nonsynony	EP300:NM	NA	.	.
FL	FL_9	FL_1639	chr22:41170420_A>T	1	EP300	1552	774	NA	2340	66%	33%	NA	exonic	nonsynony	EP300:NM	NA	.	.
FL	FL_9	FL_1639	chr17:7675218_T>C	1	TP53	744	1271	NA	2025	37%	63%	NA	exonic	nonsynony	TP53:NM_	NA	.	rs7473420
FL	FL_10	FL_1649	chr12:49026905_G>A	1	KMT2D	2852	1113	NA	3971	72%	28%	NA	exonic	stopgain	KMT2D:NM	NA	.	rs5877836
FL	FL_10	FL_1649	chr12:49051057_C>-	1	KMT2D	491	412	NA	959	51%	43%	NA	exonic	frameshift	KMT2D:NM	NA	.	.
FL	FL_10	FL_1649	chr16:3738616_C>T	1	CREBBP	617	2224	NA	2846	22%	78%	NA	exonic	nonsynony	CREBBP:NM	NA	.	.
FL	FL_11	FL_1667	chr1:2558470_T>C	1	TNFRSF14	3556	583	NA	4151	86%	14%	NA	splicing	.	.	NA	.	.
FL	FL_11	FL_1667	chr12:49038807_->C	1	KMT2D	2296	610	NA	2906	79%	21%	NA	exonic	frameshift	KMT2D:NM	NA	.	.
FL	FL_11	FL_1667	chr2:136114968_CA>-	2	CXCR4	1871	95	NA	1966	95%	5%	NA	exonic	frameshift	CXCR4:NM	NA	.	.
FL	FL_11	FL_1667	chr2:136118045_C>T	1	CXCR4	1919	266	NA	2187	88%	12%	NA	splicing	.	.	NA	.	rs1134978
FL	FL_11	FL_1667	chr22:41170517_G>T	1	EP300	1061	304	NA	1367	78%	22%	NA	exonic	nonsynony	EP300:NM	NA	.	.
FL	FL_11	FL_1667	chr6:137871522_G>-	1	TNFAIP3	1395	355	NA	1750	80%	20%	NA	exonic	frameshift	TNFAIP3:N	NA	.	.
FL	FL_12	FL_1675	chr11:108353835_T>C	1	ATM	2269	291	NA	2570	88%	11%	NA	exonic	nonsynony	ATM:NM_	NA	.	rs7803033
FL	FL_12	FL_1675	chr17:45266630_G>A	1	MAP3K14	945	664	NA	1613	59%	41%	NA	exonic	nonsynony	MAP3K14:	NA	.	rs3696468
FL	FL_13	FL_1792	chr12:49049991_GA>-	1	KMT2D	597	160	NA	757	79%	21%	NA	exonic	frameshift	KMT2D:NM	NA	.	.
FL	FL_14	FL_1801	chr1:2562895_A>C	1	TNFRSF14	262	139	NA	402	65%	35%	NA	exonic	nonsynony	TNFRSF14:	NA	.	.
FL	FL_14	FL_1801	chr12:49034851_->TC	1	KMT2D	2026	2065	NA	4091	50%	50%	NA	exonic	frameshift	KMT2D:NM	NA	.	.
FL	FL_14	FL_1801	chr16:3767720_T>-	9	CREBBP	2415	772	NA	3187	76%	24%	NA	exonic	frameshift	CREBBP:NM	NA	.	.
FL	FL_14	FL_1801	chr19:10513849_G>T	1	S1PR5	812	839	NA	1653	49%	51%	NA	exonic	nonsynony	S1PR5:NM	NA	.	rs1472075
FL	FL_15	FL_1987	chr12:49022073_GAT>-	1	KMT2D	4428	1734	NA	6162	72%	28%	NA	exonic	nonframes	KMT2D:NM	NA	.	rs5877837
FL	FL_15	FL_1987	chr12:49039989_->G	1	KMT2D	3136	1342	NA	4478	70%	30%	NA	exonic	frameshift	KMT2D:NM	NA	.	.
FL	FL_15	FL_1987	chr22:41160723_G>A	1	EP300	1222	576	NA	1798	68%	32%	NA	splicing	.	.	NA	.	.
FL	FL_16	FL_2115	chr16:3758916_G>A	1	CREBBP	1209	447	NA	1660	73%	27%	NA	exonic	stopgain	CREBBP:NM	NA	.	.
FL	FL_16	FL_2115	chr22:41170518_T>G	1	EP300	445	190	NA	635	70%	30%	NA	exonic	nonsynony	EP300:NM	NA	.	.
FL	FL_17	FL_685	chr2:236581412_G>C	1	ACKR3	1011	214	NA	1226	82%	17%	NA	exonic	nonsynony	ACKR3:NM	NA	.	.
FL	FL_17	FL_685	chr12:49026410_G>-	1	KMT2D	2938	567	NA	3505	84%	16%	NA	exonic	frameshift	KMT2D:NM	NA	.	.
FL	FL_17	FL_685	chr12:49031861_G>A	1	KMT2D	1705	405	NA	2112	81%	19%	NA	exonic	stopgain	KMT2D:NM	NA	.	.
FL	FL_17	FL_685	chr16:3736749_G>T	1	CREBBP	1525	2316	NA	3846	40%	60%	NA	exonic	nonsynony	CREBBP:NM	NA	.	.
FL	FL_17	FL_685	chr3:39266290_C>G	1	CX3CR1	1009	713	NA	1728	58%	41%	NA	exonic	nonsynony	CX3CR1:NM	NA	.	.
FL	FL_18	FL_804	chr12:49048054_A>T	1	KMT2D	2818	1202	NA	4037	70%	30%	NA	exonic	nonsynony	KMT2D:NM	NA	.	.
FL	FL_18	FL_804	chr16:3736762_A>T	1	CREBBP	3376	1387	NA	4779	71%	29%	NA	exonic	nonsynony	CREBBP:NM	NA	.	.
FL	FL_18	FL_804	chr19:41880734_ATG>-	1	CD79A	329	87	NA	416	79%	21%	NA	exonic	nonframes	CD79A:NM	NA	.	.
FL	FL_18	FL_804	chr22:41129965_T>C	2	EP300	364	154	NA	518	70%	30%	NA	exonic	nonsynony	EP300:NM	NA	.	.
FL	FL_18	FL_804	chr17:7673777_G>C	1	TP53	1226	723	NA	1956	63%	37%	NA	exonic	nonsynony	TP53:NM_	NA	.	.
FL	FL_18	FL_804	chr3:151881045_A>C	1	SUCNR1	1033	286	NA	1322	78%	22%	NA	exonic	nonsynony	SUCNR1:NM	NA	.	.
FL	FL_19	FL_817	chr12:49044447_T>C	1	KMT2D	1032	136	NA	1170	88%	12%	NA	exonic	nonsynony	KMT2D:NM	NA	.	.
FL	FL_19	FL_817	chr12:49054620_G>-	1	KMT2D	732	197	NA	929	79%	21%	NA	exonic	frameshift	KMT2D:NM	NA	.	.
FL	FL_19	FL_817	chr16:3745275_A>C	1	CREBBP	795	316	NA	1116	71%	28%	NA	splicing	.	.	NA	.	.
FL	FL_19	FL_817	chr17:63929260_->TA	1	CD79B	1625	444	NA	2069	79%	21%	NA	exonic	frameshift	CD79B:NM	NA	.	.
MALT_GA	GM_1	MALT_GA_28549_12	chr1:26780628_G>T	2	ARID1A	19	16	NA	36	53%	44%	NA	exonic	nonsynony	ARID1A:NM	TRUE	.	rs3728787
MALT_GA	GM_2	MALT_GA_G0735	chr4:105235551_G>T	2	TET2	218	307	NA	526	41%	58%	NA	exonic	stopgain	TET2:NM_	TRUE	.	.
MALT_GA	GM_2	MALT_GA_G0735	chr6:111375303_C>T	2	REV3L	719	454	NA	1176	61%	39%	NA	exonic	nonsynony	REV3L:NM	TRUE	.	rs3757019
MALT_GA	GM_3	MALT_GA_G0906	chr1:2558381_A>G	2	TNFRSF14	378	234	NA	612	62%	38%	NA	exonic	nonsynony	TNFRSF14:	TRUE	.	.
MALT_GA	GM_4	MALT_GA_G3604	chr6:137877081_C>T	2	TNFAIP3	375	181	NA	558	67%	32%	NA	exonic	stopgain	TNFAIP3:N	TRUE	.	.
MALT_LU	LM_2	MALT_LU_01H1968	chr14:100277460_A>G	1	YY1	34	22	NA	56	61%	39%	NA	exonic	nonsynony	YY1:NM_0	NA	.	.
MALT_LU	LM_2	MALT_LU_01H1968	chr12:49022670_G>T	1	KMT2D	180	28	NA	209	86%	13%	NA	exonic	nonsynony	KMT2D:NM	NA	.	.
MALT_LU	LM_2	MALT_LU_01H1968	chr3:177033051_A>C	1	TBL1XR1	83	38	NA	121	69%	31%	NA	exonic	nonsynony	TBL1XR1:N	NA	.	.
MALT_LU	LM_1	MALT_LU_AN2	chr11:118894569_GTACC>-	1	CXCR5	183	51	NA	234	78%	22%	NA	exonic	frameshift	CXCR5:NM	NA	.	.
MALT_OA	OM_1	MALT_OA_12_2353	chr20:35632818_C>T	1	CPNE1	990	990	NA	1980	50%	50%	NA	exonic	nonsynony	CPNE1:NM	NA	.	rs1472012
MALT_OA	OM_1	MALT_OA_12_2353	chr3:38140801_G>A	1	MYD88	1436	1126	NA	2562	56%	44%	NA	exonic	nonsynony	MYD88:NM	NA	.	.
MALT_OA	OM_1	MALT_OA_12_2353	chr16:3770884_T>C	1	CREBBP	793	744	NA	1545	51%	48%	NA	exonic	nonsynony	CREBBP:NM	NA	.	rs3735312
MALT_OA	OM_2	MALT_OA_12_2576	chr1:202304624_G>A	1	LGR6	144	125	NA	269	54%	46%	NA	exonic	nonsynony	LGR6:NM_	NA	.	rs5616142
MALT_OA	OM_3	MALT_OA_14_1615	chr18:62361721_T>G	1	TNFRSF11	90	134	NA	226	40%	59%	NA	exonic	nonsynony	TNFRSF11	NA	.	rs2003410
MALT_OA	OM_3	MALT_OA_14_1615	chr12:71440268_A>G	1	LGR5	205	175	NA	380	54%	46%	NA	exonic	nonsynony	LGR5:NM_	NA	.	.
MALT_OA	OM_3	MALT_OA_14_1615	chr6:137877127_->A	2	TNFAIP3	583	256	NA	839	69%	31%	NA	exonic	frameshift	TNFAIP3:N	NA	.	.
MALT_OA	OM_4	MALT_OA_14_1639	chr3:132601236_G>A	1	ACKR4	300	185	NA	486	62%	38%	NA	exonic	nonsynony	ACKR4:NM	NA	.	rs7702905
MALT_OA	OM_4	MALT_OA_14_1639	chr6:137877127_->A	2	TNFAIP3	772	176	NA	948	81%	19%	NA	exonic	frameshift	TNFAIP3:N	NA	.	.
MALT_OA	OM_5	MALT_OA_14_1744	chr6:137875997_->A	1	TNFAIP3	699	127	NA	826	85%	15%	NA	exonic	frameshift	TNFAIP3:N	NA	.	.
MALT_OA	OM_6	MALT_OA_14_512	chr11:108250861_C>G	1	ATM	754	505	NA	1263	60%	40%	NA	exonic	nonsynony	ATM:NM_	NA	.	.
MALT_OA	OM_7	MALT_OA_14_851	chr3:38141150_T>C	1	MYD88	476	232	NA	708	67%	33%	NA	exonic	nonsynony	MYD88:NM	NA	0.001	rs3879072
MALT_OA	OM_7	MALT_OA_14_851	chr9:136497388_G>T	1	NOTCH1	88	110	NA	198	44%	56%	NA	exonic	nonsynony	NOTCH1:N	NA	.	rs7555097
MALT_OA	OM_8	MALT_OA_2004_1021	chr22:41157247_C>T	1	EP300	282	220	NA	504	56%	44%	NA	exonic	nonsynony	EP300:NM	NA	.	rs7476917
MALT_OA	OM_8	MALT_OA_2004_1021	chr6:137876094_A>T	1	TNFAIP3	52	137	NA	190	27%	72%	NA	exonic	stopgain	TNFAIP3:N	NA	.	.
MALT_OA	OM_9	MALT_OA_2005_1199	chr9:5457211_A>T	1	CD274	156	18	NA	174	90%	10%	NA	exonic	nonsynony	CD274:NM	NA	.	.
MALT_OA	OM_10	MALT_OA_2005_1318	chr1:111500224_G>A	1	ADORA3	529	220	NA	749	71%	29%	NA	exonic	nonsynony	ADORA3:N	NA	.	rs7574728
MALT_OA	OM_10	MALT_OA_2005_1318	chr6:137877108_CGGGGAAGATT>	1	TNFAIP3	94	118	NA	212	44%	56%	NA	exonic	frameshift	TNFAIP3:N	NA	.	.
MALT_OA	OM_11	MALT_OA_2005_1953	chr1:85267963_A>C	1	BCL10	804	7061	NA	7881	10%	90%	NA	exonic	nonsynony	BCL10:NM	NA	.	.
MALT_OA	OM_11	MALT_OA_2005_1953	chr17:7675095_C>T	1	TP53	126	188	NA	314	40%	60%	NA	exonic	nonsynony	TP53:NM_	NA	.	.
MALT_OA	OM_12	MALT_OA_2006_1992	chr5:151042900_T>G	1	TNIP1	106	88	NA	194	55%	45%	NA	exonic	nonsynony	TNIP1:NM	NA	.	rs1482430
MALT_OA	OM_12	MALT_OA_2006_1992	chr6:137878939_C>A	1	TNFAIP3	174	207	NA	382	46%	54%	NA	exonic	stopgain	TNFAIP3:N	NA	.	.
MALT_OA	OM_13	MALT_OA_2006_2154	chr6:106105327_C>-	1	PRDM1	96	124	NA	220	44%	56%	NA	exonic	frameshift	PRDM1:NM	NA	.	.
MALT_OA	OM_13	MALT_OA_2006_2154	chr6:137878808_G>-	1	TNFAIP3	309	139	NA	448	69%	31%	NA	exonic	frameshift	TNFAIP3:N	NA	.	.
MALT_OA	OM_14	MALT_OA_2006_2190	chr3:39332648_G>A	1	CCR8	134	168	NA	304	44%	55%	NA	exonic	nonsynony	CCR8:NM_	NA	.	rs7614843
MALT_OA	OM_14	MALT_OA_2006_2190	chr9:5462968_G>-	1	CD274	859	199	NA	1058	81%	19%	NA	exonic	frameshift	CD274:NM	NA	.	.
MALT_OA	OM_15	MALT_OA_2006_2319	chr1:2557774_C>T	1	TNFRSF14	97	71	NA	168	58%	42%	NA	exonic	nonsynony	TNFRSF14:	NA	.	.
MALT_OA	OM_16	MALT_OA_2006_68	chr2:240630850_A>G	1	GPR35	100	131	NA	231	43%	57%	NA	exonic	nonsynony	GPR35:NM	NA	.	rs7492943
MALT_OA	OM_16	MALT_OA_2006_68	chr4:105237229_C>G	1	TET2	172	47	NA	219	79%	21%	NA	exonic	nonsynony	TET2:NM_	NA	.	.
MALT_OA	OM_16	MALT_OA_2006_68	chr6:137871524_T>C	1	TNFAIP3	246	73	NA	319	77%	23%	NA	splicing	.	.	NA	.	.
MALT_OA	OM_16	MALT_OA_2006_68	chr6:137874953_->A	1	TNFAIP3	745	188	NA	933	80%	20%	NA	exonic	frameshift	TNFAIP3:N	NA	.	.
MALT_OA	OM_17	MALT_OA_2007_1678	chr16:3767778_C>-	1	CREBBP	1803	84	NA	1887	96%	4%	NA	exonic	frameshift	CREBBP:NM	NA	.	.
MALT_OA	OM_17	MALT_OA_2007_1678	chr3:46373330_C>T	1	CCR5	443	473	NA	919	48%	51%	NA	exonic	nonsynony	CCR5:NM_	NA	.	.

Supplementary Table S5: Variants detected by 93-gene panel sequencing.

MALT_OA	OM_18	MALT_OA_2007_1697	chr18:62369319_C>T	1	TNFRSF11/	646	672	NA	1319	49%	51%	NA	exonic	nonsynony	TNFRSF11/	NA	.	rs7482114
MALT_OA	OM_19	MALT_OA_2007_201_	chr3:177051507_->TA	1	TBL1XR1	175	472	NA	647	27%	73%	NA	exonic	stopgain	TBL1XR1:N	NA	.	.
MALT_OA	OM_19	MALT_OA_2007_201_	chr5:76733022_A>G	1	F2R	235	219	NA	457	51%	48%	NA	exonic	nonsynony	F2R:NM_0	NA	.	rs1444475
MALT_OA	OM_20	MALT_OA_2007_2072	chr3:183555481_A>G	1	KLHL6	112	75	NA	187	60%	40%	NA	exonic	nonsynony	KLHL6:NM_	NA	.	rs7493980
MALT_OA	OM_21	MALT_OA_2007_467	chr6:137878727_G>T	1	TNFAIP3	71	97	NA	169	42%	57%	NA	exonic	stopgain	TNFAIP3:N	NA	.	.
MALT_OA	OM_22	MALT_OA_2008_1245	chr17:40555631_T>C	1	CCR7	320	309	NA	629	51%	49%	NA	exonic	nonsynony	CCR7:NM_	NA	.	.
MALT_OA	OM_22	MALT_OA_2008_1245	chr16:3728424_TGTTGCTGCTGC>-	1	CREBBP	86	80	NA	166	52%	48%	NA	exonic	nonframes	CREBBP:NI	NA	.	rs7786786
MALT_OA	OM_22	MALT_OA_2008_1245	chr1:119916544_G>A	1	NOTCH2	78	76	NA	155	50%	49%	NA	exonic	nonsynony	NOTCH2:N	NA	.	rs7465518
MALT_OA	OM_23	MALT_OA_2008_468	chr6:137871518_->A	1	TNFAIP3	652	266	NA	918	71%	29%	NA	exonic	frameshift	TNFAIP3:N	NA	.	.
MALT_OA	OM_23	MALT_OA_2008_468	chrX:40055449_C>A	1	BCOR	1249	797	NA	2051	61%	39%	NA	exonic	nonsynony	BCOR:NM_	NA	.	.
MALT_OA	OM_23	MALT_OA_2008_468	chr6:137877093_->TTGT	1	TNFAIP3	506	140	NA	646	78%	22%	NA	exonic	frameshift	TNFAIP3:N	NA	.	.
MALT_OA	OM_24	MALT_OA_2008_739	chr6:137871249_C>T	1	TNFAIP3	443	49	NA	493	90%	10%	NA	exonic	stopgain	TNFAIP3:N	NA	.	.
MALT_OA	OM_24	MALT_OA_2008_739	chr6:137871519_A>G	1	TNFAIP3	277	46	NA	324	85%	14%	NA	exonic	nonsynony	TNFAIP3:N	NA	.	.
MALT_OA	OM_25	MALT_OA_2008_795	chr12:49040199_G>A	1	KMT2D	265	257	NA	523	51%	49%	NA	exonic	nonsynony	KMT2D:NM	NA	.	rs7602630
MALT_OA	OM_25	MALT_OA_2008_795	chr3:46373909_G>T	1	CCR5	227	213	NA	440	52%	48%	NA	exonic	nonsynony	CCR5:NM_	NA	.	rs7650099
MALT_SA	SM_1	MALT_SA_07HS27466P	chr6:106088228_G>A	2	PRDM1	148	85	NA	237	62%	36%	NA	exonic	nonsynony	PRDM1:NM	TRUE	.	rs7561562
MALT_SA	SM_2	MALT_SA_1408_0295	chr1:2561777_T>G	1	TNFRSF14	756	739	NA	1498	50%	49%	NA	exonic	nonsynony	TNFRSF14:	NA	.	.
MALT_SA	SM_2	MALT_SA_1408_0295	chr12:49046259_C>G	1	KMT2D	620	209	NA	831	75%	25%	NA	splicing	.	.	NA	.	.
MALT_SA	SM_2	MALT_SA_1408_0295	chr11:108326077_T>G	1	ATM	236	147	NA	385	61%	38%	NA	exonic	nonsynony	ATM:NM_(	NA	.	rs7533896
MALT_SA	SM_2	MALT_SA_1408_0295	chr16:3738648_A>C	1	CREBBP	880	290	NA	1177	75%	25%	NA	exonic	nonsynony	CREBBP:NI	NA	.	.
MALT_SA	SM_2	MALT_SA_1408_0295	chr3:177050074_ACTGTGTAGAGC	1	TBL1XR1	280	450	NA	730	38%	62%	NA	exonic	frameshift	TBL1XR1:N	NA	.	.
MALT_SA	SM_3	MALT_SA_1709_6793C	chr3:46204201_G>A	1	CCR1	570	411	NA	985	58%	42%	NA	exonic	nonsynony	CCR1:NM_	NA	.	.
MALT_SA	SM_3	MALT_SA_1709_6793C	chr6:137874903_AG>-	1	TNFAIP3	1694	110	NA	1804	94%	6%	NA	exonic	frameshift	TNFAIP3:N	NA	.	.
MALT_SA	SM_4	MALT_SA_18319_13	chr3:177038425_A>G	1	TBL1XR1	248	63	NA	312	79%	20%	NA	exonic	nonsynony	TBL1XR1:N	NA	.	.
MALT_SA	SM_4	MALT_SA_18319_13	chr4:105234621_->A	2	TET2	380	464	NA	844	45%	55%	NA	exonic	frameshift	TET2:NM_	NA	.	rs7590555
MALT_SA	SM_4	MALT_SA_18319_13	chr7:2939888_TCC>-	1	CARD11	351	47	NA	398	88%	12%	NA	exonic	nonframes	CARD11:NI	NA	.	.
MALT_SA	SM_4	MALT_SA_18319_13	chrX:41696651_C>T	1	GPR34	76	26	NA	104	73%	25%	NA	exonic	stopgain	GPR34:NM	NA	.	.
MALT_SA	SM_5	MALT_SA_204_07	chr3:177047350_A>C	2	TBL1XR1	382	91	NA	475	80%	19%	NA	exonic	nonsynony	TBL1XR1:N	TRUE	.	.
MALT_SA	SM_5	MALT_SA_204_07	chr3:177047371_G>C	2	TBL1XR1	702	103	NA	806	87%	13%	NA	exonic	nonsynony	TBL1XR1:N	TRUE	.	.
MALT_SA	SM_6	MALT_SA_2644_98	chr12:49040739_C>T	2	KMT2D	145	187	NA	337	43%	55%	NA	exonic	nonsynony	KMT2D:NM	TRUE	.	rs1998452
MALT_SA	SM_6	MALT_SA_2644_98	chr3:177046135_GAA>-	2	TBL1XR1	745	88	NA	833	89%	11%	NA	exonic	nonframes	TBL1XR1:N	TRUE	.	.
MALT_SA	SM_6	MALT_SA_2644_98	chr17:45287254_C>T	2	MAP3K14	353	394	NA	749	47%	53%	NA	exonic	nonsynony	MAP3K14:	TRUE	.	.
MALT_SA	SM_7	MALT_SA_64_02	chr14:102903403_GC>-	1	TRAF3	632	313	NA	945	67%	33%	NA	exonic	frameshift	TRAF3:NM	NA	.	.
MALT_SA	SM_7	MALT_SA_64_02	chr14:100239422_G>A	1	YY1	637	145	NA	790	81%	18%	NA	exonic	nonsynony	YY1:NM_0	NA	.	.
MALT_SA	SM_8	MALT_SA_8528_10	chr6:137871402_C>T	2	TNFAIP3	1386	325	NA	1713	81%	19%	NA	exonic	stopgain	TNFAIP3:N	TRUE	.	.
MALT_SA	SM_9	MALT_SA_G1348	chr3:177050136_G>A	2	TBL1XR1	16	33	NA	49	33%	67%	NA	exonic	nonsynony	TBL1XR1:N	TRUE	.	.
MALT_SA	SM_10	MALT_SA_G1915	chrX:1466507_G>A	2	P2RY8	464	306	NA	778	60%	39%	NA	exonic	nonsynony	P2RY8:NM	TRUE	.	.
MALT_SF	SFM_1	MALT_SF_S1700948	chr1:85267854_C>A	1	BCL10	488	57	NA	546	89%	10%	NA	exonic	stopgain	BCL10:NM	NA	.	.
MALT_SF	SFM_1	MALT_SF_S1700948	chr1:85267646_C>T	1	BCL10	470	97	NA	567	83%	17%	NA	exonic	nonsynony	BCL10:NM	NA	.	.
MALT_TH	TM_1	MALT_TH_10_1442	chr13:32337561_C>T	1	BRCA2	934	102	NA	1036	90%	10%	NA	exonic	nonsynony	BRCA2:NM	NA	.	rs8035856
MALT_TH	TM_1	MALT_TH_10_1442	chr12:49026194_->GTCA	1	KMT2D	1341	246	NA	1587	84%	16%	NA	exonic	stopgain	KMT2D:NM	NA	.	.
MALT_TH	TM_1	MALT_TH_10_1442	chr4:105236667_C>T	1	TET2	1385	188	NA	1573	88%	12%	NA	exonic	stopgain	TET2:NM_	NA	.	rs7542871
MALT_TH	TM_2	MALT_TH_10_350	chr4:105275493_T>A	1	TET2	106	14	NA	121	88%	12%	NA	exonic	stopgain	TET2:NM_	NA	.	.
MALT_TH	TM_2	MALT_TH_10_350	chr6:137874944_AAACAGACACAC	1	TNFAIP3	1977	460	NA	2437	81%	19%	NA	exonic	frameshift	TNFAIP3:N	NA	.	.
MALT_TH	TM_3	MALT_TH_12_1365	chr3:38140543_A>T	1	MYD88	241	264	NA	505	48%	52%	NA	exonic	nonsynony	MYD88:NM	NA	.	.
MALT_TH	TM_3	MALT_TH_12_1365	chr4:105272735_C>T	3	TET2	319	47	NA	367	87%	13%	NA	exonic	stopgain	TET2:NM_	NA	.	.
MALT_TH	TM_4	MALT_TH_12_149R	chr19:41877383_GGTATGTGGCCA	1	CD79A	3801	94	NA	3895	98%	2%	NA	exonic	frameshift	CD79A:NM	NA	.	.
MALT_TH	TM_4	MALT_TH_12_149R	chr5:151064972_T>A	1	TNIP1	1067	997	NA	2077	51%	48%	NA	exonic	nonsynony	TNIP1:NM_	NA	.	.
MALT_TH	TM_4	MALT_TH_12_149R	chr1:2559903_G>T	1	TNFRSF14	2651	894	NA	3563	74%	25%	NA	exonic	nonsynony	TNFRSF14:	NA	.	.
MALT_TH	TM_4	MALT_TH_12_149R	chr4:105275065_G>-	1	TET2	304	216	NA	520	58%	42%	NA	exonic	frameshift	TET2:NM_	NA	.	.
MALT_TH	TM_4	MALT_TH_12_149R	chr6:137876064_C>T	1	TNFAIP3	855	67	NA	925	92%	7%	NA	exonic	nonsynony	TNFAIP3:N	NA	.	.
MALT_TH	TM_5	MALT_TH_13_1042	chr6:111380205_C>T	1	REV3L	410	59	NA	471	87%	13%	NA	exonic	nonsynony	REV3L:NM	NA	.	.
MALT_TH	TM_5	MALT_TH_13_1042	chr1:2559906_C>T	1	TNFRSF14	844	342	NA	1186	71%	29%	NA	exonic	stopgain	TNFRSF14:	NA	.	.
MALT_TH	TM_5	MALT_TH_13_1042	chr11:118894626_->T	3	CXCR5	1155	32	NA	1187	97%	3%	NA	exonic	frameshift	CXCR5:NM	NA	.	.
MALT_TH	TM_5	MALT_TH_13_1042	chr4:105259763_A>-	1	TET2	260	96	NA	356	73%	27%	NA	exonic	frameshift	TET2:NM_	NA	.	.
MALT_TH	TM_5	MALT_TH_13_1042	chr9:5456167_T>A	1	CD274	32	56	NA	88	36%	64%	NA	splicing	.	.	NA	.	.
MALT_TH	TM_6	MALT_TH_13_1086	chr1:2558447_C>T	1	TNFRSF14	535	122	NA	660	81%	18%	NA	exonic	stopgain	TNFRSF14:	NA	.	.
MALT_TH	TM_6	MALT_TH_13_1086	chr3:183492545_C>G	1	KLHL6	298	65	NA	363	82%	18%	NA	exonic	nonsynony	KLHL6:NM	NA	.	.
MALT_TH	TM_6	MALT_TH_13_1086	chr4:105275863_A>-	1	TET2	1048	190	NA	1238	85%	15%	NA	exonic	frameshift	TET2:NM_	NA	.	.
MALT_TH	TM_6	MALT_TH_13_1086	chr9:5462940_G>A	1	CD274	689	287	NA	977	71%	29%	NA	exonic	stopgain	CD274:NM	NA	.	.
MALT_TH	TM_7	MALT_TH_13_1127	chr13:32339966_AGTAA>-	1	BRCA2	39	37	NA	76	51%	49%	NA	exonic	frameshift	BRCA2:NM	NA	.	rs7630697
MALT_TH	TM_8	MALT_TH_13_1250	chr17:7674229_C>G	1	TP53	2066	316	NA	2419	85%	13%	NA	exonic	nonsynony	TP53:NM_	NA	.	.
MALT_TH	TM_8	MALT_TH_13_1250	chr4:105235529_C>A	1	TET2	564	132	NA	696	81%	19%	NA	exonic	stopgain	TET2:NM_	NA	.	.
MALT_TH	TM_8	MALT_TH_13_1250	chrX:71616412_C>-	1	CXCR3	431	34	NA	465	93%	7%	NA	exonic	frameshift	CXCR3:NM	NA	.	.
MALT_TH	TM_9	MALT_TH_13_1285_3	chrX:40062170_A>C	1	BCOR	691	104	NA	797	87%	13%	NA	exonic	nonsynony	BCOR:NM_	NA	.	.
MALT_TH	TM_9	MALT_TH_13_1285_3	chr17:7675080_G>C	1	TP53	1318	133	NA	1451	91%	9%	NA	exonic	nonsynony	TP53:NM_	NA	.	.
MALT_TH	TM_9	MALT_TH_13_1285_3	chr4:105236247_C>T	1	TET2	280	38	NA	318	88%	12%	NA	exonic	stopgain	TET2:NM_	NA	.	rs7512331
MALT_TH	TM_9	MALT_TH_13_1285_3	chr4:105243630_CA>-	1	TET2	261	45	NA	306	85%	15%	NA	exonic	frameshift	TET2:NM_	NA	.	.
MALT_TH	TM_9	MALT_TH_13_1285_3	chr9:5463025_A>C	1	CD274	1976	637	NA	2615	76%	24%	NA	exonic	nonsynony	CD274:NM	NA	.	.
MALT_TH	TM_10	MALT_TH_13_1429	chr1:2559922_G>T	1	TNFRSF14	1248	614	NA	1867	67%	33%	NA	exonic	nonsynony	TNFRSF14:	NA	.	.
MALT_TH	TM_10	MALT_TH_13_1429	chr22:41131635_T>G	1	EP300	273	35	NA	310	88%	11%	NA	splicing	.	.	NA	.	.
MALT_TH	TM_10	MALT_TH_13_1429	chr14:100239503_C>T	1	YY1	1025	202	NA	1232	83%	16%	NA	exonic	stopgain	YY1:NM_0	NA	.	.
MALT_TH	TM_10	MALT_TH_13_1429	chr11:118883994_T>G	1	CXCR5	150	30	NA	180	83%	17%	NA	splicing	.	.	NA	.	.
MALT_TH	TM_10	MALT_TH_13_1429	chr3:177050509_A>G	1	TBL1XR1	456	125	NA	583	78%	21%	NA	exonic	nonsynony	TBL1XR1:N	NA	.	.
MALT_TH	TM_10	MALT_TH_13_1429	chr4:105269710_A>G	1	TET2	673	129	NA	802	84%	16%	NA	exonic	nonsynony	TET2:NM_	NA	.	.
MALT_TH	TM_10	MALT_TH_13_1429	chr4:105275267_C>G	1	TET2	138	57	NA	196	70%	29%	NA	exonic	stopgain	TET2:NM_	NA	.	.
MALT_TH	TM_10	MALT_TH_13_1429	chr9:5465549_G>A	1	CD274	638	100	NA	741	86%	13%	NA	exonic	nonsynony	CD274:NM	NA	.	.
MALT_TH	TM_26	MALT_TH_14_915	chr1:111500530_C>A	1	ADORA3	703	240	NA	944	74%	25%	NA	exonic	nonsynony	ADORA3:N	NA	.	.
MALT_TH	TM_11	MALT_TH_13_1853	chr3:183492154_C>T	1	KLHL6	680	174	NA	856	79%	20%	NA	exonic	nonsynony	KLHL6:NM	NA	.	.
MALT_TH	TM_11	MALT_TH_13_1853	chr2:240630312_C>G	1	GPR35	106	79	NA	186	57%	42%	NA	exonic	nonsynony	GPR35:NM	NA	.	.
MALT_TH	TM_11	MALT_TH_13_1853	chr4:105236751_->A	1	TET2	563	20	NA	583	97%	3%	NA	exonic	frameshift	TET2:NM_	NA	.	.
MALT_TH	TM_11	MALT_TH_13_1853	chr4:105272653_T>-	1	TET2	1708	679	NA	2387	72%	28%	NA	exonic	frameshift	TET2:NM_	NA	.	.
MALT_TH	TM_12	MALT_TH_13_273	chr1:2557822_A>T	1	TNFRSF14	405	702	NA	1114	36%	63%	NA	exonic	stopgain	TNFRSF14:	NA	.	.
MALT_TH	TM_12	MALT_TH_13_273	chr11:118894592_C>T	1	CXCR5	1032	224	NA	1261	82%	18%	NA	exonic	stopgain	CXCR5:NM	NA	.	.
MALT_TH	TM_12	MALT_TH_13_273	chr19:14391060_G>C	1	ADGRE5	122	107	NA	230	53%	47%	NA	exonic	nonsynony	ADGRE5:N	NA	.	.
MALT_TH	TM_12	MALT_TH_13_273	chr2:11669546_G>A	1	NTSR2	878	345	NA	1224	72%	28%	NA	exonic	nonsynony	NTSR2:NM	NA	.	.
MALT_TH	TM_12	MALT_TH_13_273	chr16:3792074_G>A	1	CREBBP	127	46	NA	174	73%	26%	NA	exonic	stopgain	CREBBP:NI	NA	.	.
MALT_TH	TM_12	MALT_TH_13_273	chr16:3850812_C>T	1	CREBBP	559	680	NA	1248	45%	54%	NA	exonic	nonsynony	CREBBP:NI	NA	.	rs7568029
MALT_TH	TM_12	MALT_TH_13_273	chr19:16326917_G>C	3	KLF2	776	280	NA	1059	73%	26%	NA	exonic	nonsynony	KLF2:NM_(	NA	.	.
MALT_TH	TM_12	MALT_TH_13_273	chr4:105275267_C>A	1	TET2	362	110	NA	473	77%	23%	NA	exonic	stopgain	TET2:NM_	NA	.	.
MALT_TH	TM_12	MALT_TH_13_273	chr9:5457393_T>C	1	CD274	541	823	NA	1365	40%	60%	NA	exonic	nonsynony	CD274:NM	NA	.	.
MALT_TH	TM_12	MALT_TH_13_273	chrX:71616401_C>T	1	CXCR3	737	206	NA	944	78%	22%	NA	exonic	stopgain	CXCR3:NM	NA	.	.
MALT_TH	TM_13	MALT_TH_13_356	chr1:26774881_C>T	1	ARID1A	776	221	NA	997	78%	22%	NA	exonic	stopgain	ARID1A:NM	NA	.	.
MALT_TH	TM_49	MALT_TH_16_763	chr1:111500293_C>T	1	ADORA3	111	82	NA	193	58%	42%	NA	exonic	nonsynony	ADORA3:N	NA	.	rs7641242
MALT_TH	TM_13	MALT_TH_13_356	chr6:111387886_G>T	1	REV3L	56	90	NA	146	38%	62%	NA	exonic	nonsynony	REV3L:NM	NA	.	.
MALT_TH	TM_13	MALT_TH_13_356	chr3:177038386_C>T	1	TBL1XR1	344	86	NA	431	80%	20%	NA	exonic	nonsynony	TBL1XR1:N	NA	.	.
MALT_TH	TM_13	MALT_TH_13_356	chr4:105237215_ACCA>-	1	TET2	737	338	NA	1075	69%	31%	NA	exonic	frameshift	TET2:NM_	NA	.	.
MALT_TH	TM_13	MALT_TH_13_356	chr6:137874985_TC>-	1	TNFAIP3	989	309	NA	1400	71%	22%	NA	exonic	frameshift	TNFAIP3:N	NA	.	.
MALT_TH	TM_13	MALT_TH_13_356	chr9:5456116_G>A	1	CD274	14	16	NA	30	47%	53%	NA	exonic	nonsynony	CD274:NM	NA	.	rs1114012
MALT_TH	TM_14	MALT_TH_13_398	chr1:2557718_CCCCTTAGG>-	1	TNFRSF14	208	82	NA	290	72%	28%	NA	exonic	frameshift	TNFRSF14:	NA	.	.
MALT_TH	TM_14	MALT_TH_13_398	chr14:105051818_G>A	1	GPR132	667	99	NA	767	87%	13%	NA	exonic	nonsynony	GPR132:NI	NA	.	rs2010022
MALT_TH	TM_14	MALT_TH_13_398	chr19:16325351_C>T	1	KLF2	151	25	NA	176	86%	14%	NA	exonic	nonsynony	KLF2:NM_(	NA	.	.
MALT_TH	TM_14	MALT_TH_13_398	chr19:16326917_G>C	3	KLF2	463	86	NA	549	84%	16%	NA	exonic	nonsynony	KLF2:NM_(	NA	.	.
MALT_TH	TM_14	MALT_TH_13_398	chr2:136115474_C>T	1	CXCR4	870	105	NA	976	89%	11%	NA	exonic	nonsynony	CXCR4:NM	NA	.	.
MALT_TH	TM_14	MALT_TH_13_398	chr4:105243607_G>A	1	TET2	348	97	NA	446	78%	22%	NA	exonic	nonsynony	TET2:NM_	NA	.	.
MALT_TH	TM_14	MALT_TH_13_398	chr4:105272644_C>G	1	TET2	2338	678	NA	3017	77%	22%	NA	exonic	stopgain	TET2:NM_	NA	.	.
MALT_TH	TM_14	MALT_TH_13_398	chr9:5465607_G>A	1	CD274	935	372	NA	1309	71%	28%	NA	splicing	.	.	NA	.	.
MALT_TH	TM_14	MALT_TH_13_398	chrX:71617027_G>A	1	CXCR3	1544	413	NA	1957	79%	21%	NA	exonic	nonsynony	CXCR3:NM	NA	.	.
MALT_TH	TM_15	MALT_TH_13_574	chr12:49022042_CCT>-	1	KMT2D	412	163	NA	575	72%	28%	NA	exonic	frameshift	KMT2D:NM	NA	.	.
MALT_TH	TM_11	MALT_TH_13_1853	chr11:108267171_G>A	1	ATM	498	209	NA	714	70%	29%	NA	exonic	nonsynony	ATM:NM_(	NA	.	.
MALT_TH	TM_15	MALT_TH_13_574	chr4:105237281_->A	1	TET2	52	36	NA	88	59%	41%	NA	exonic	frameshift	TET2:NM_	NA	.	.
MALT_TH	TM_15	MALT_TH_13_574	chr4:105275863_A>T	1	TET2	1638	907	NA	2556	64%	35%	NA	exonic	stopgain	TET2:NM_	NA	.	.
MALT_TH	TM_16	MALT_TH_13536_02	chr5:151045868_C>T	1	TNIP1	2331	384	NA	2721	86%	14%	NA	exonic	nonsynony	TNIP1:NM_	NA	.	.
MALT_TH	TM_17	MALT_TH_14_1195	chr4:105235795_A>-	1	TET2	295	23	NA	318	93%	7%	NA	exonic	frameshift	TET2:NM_	NA	.	.
MALT_TH	TM_17	MALT_TH_14_1195	chr4:105236640_A>-	1	TET2	532	62	NA	594	90%	10%	NA	exonic	frameshift	TET2:NM_	NA	.	rs7511930
MALT_TH	TM_18	MALT_TH_14_1279	chr1:2561743_G>A	1	TNFRSF14	1310	432	NA	1748	75%	25%	NA	exonic	nonsynony	TNFRSF14:	NA	.	.
MALT_TH	TM_18	MALT_TH_14_1279	chr19:16326917_G>C	3	KLF2	603	112	NA	716	84%	16%	NA	exonic	nonsynony	KLF2:NM_(	NA	.	.
MALT_TH	TM_18	MALT_TH_14_1279	chr4:105259627_G>C	1	TET2	462	121	NA	587	79%	21%	NA	exonic	nonsynony	TET2:NM_	NA	.	.
MALT_TH	TM_47	MALT_TH_16_541	chr11:108365473_A>G	1	ATM	554	318	NA	877	63%	36%	NA	exonic	nonsynony	ATM:NM_(	NA	.	.
MALT_TH	TM_18	MALT_TH_14_1279	chr9:5457141_G>T	3	CD274	27	15	NA	42	64%	36%	NA	exonic	stopgain	CD274:NM	NA	.	.
MALT_TH	TM_19	MALT_TH_14_1315	chr1:2559922_GCGCCGC>-	1	TNFRSF14	2517	813	NA	3330	76%	24%	NA	exonic	frameshift	TNFRSF14:	NA	.	.
MALT_TH	TM_19	MALT_TH_14_1315	chr1:26760958_AATCCAG>-	1	ARID1A	2436	107	NA	2543	96%	4%	NA	exonic	frameshift	ARID1A:NM	NA	.	.
MALT_TH	TM_19	MALT_TH_14_1315	chr13:32338722_A>C	1	BRCA2	427	247	NA	675	63%	37%	NA	exonic	nonsynony	BRCA2:NM	NA	.	.
MALT_TH	TM_13	MALT_TH_13_356	chr4:101790884_C>G	1	BANK1	838	336	NA	1176	71%	29%	NA	exonic	nonsynony	BANK1:NM	NA	.	.
MALT_TH	TM_19	MALT_TH_14_1315	chr4:105235239_->A	1	TET2	567	83	NA	650	87%	13%	NA	exonic	frameshift	TET2:NM_	NA	.	.
MALT_TH	TM_19	MALT_TH_14_1315	chr9:5462899_A>C	1	CD274	895	246	NA	1142	78%	22%	NA	exonic	nonsynony	CD274:NM	NA	.	.
MALT_TH	TM_20	MALT_TH_14_1501	chr1:2556667_G>A	1	TNFRSF14	185	64	NA	249	74%	26%	NA	exonic	nonsynony	TNFRSF14:	NA	.	.
MALT_TH	TM_20	MALT_TH_14_1501	chr4:105234508_G>-	1	TET2	1062	301	NA	1363	78%	22%	NA	exonic	frameshift	TET2:NM_	NA	.	.
MALT_TH	TM_20	MALT_TH_14_1501	chr4:105259627_G>A	1	TET2	369	51	NA	422	87%	12%	NA	exonic	nonsynony	TET2:NM_	NA	.	.
MALT_TH	TM_21	MALT_TH_14_1573	chr4:105237030_C>T	1	TET2	141	25	NA	166	85%	15%	NA	exonic	stopgain	TET2:NM_	NA	.	rs7800439
MALT_TH	TM_21	MALT_TH_14_1573	chr4:105243754_A>G	1	TET2	91	297	NA	388	23%	77%	NA	exonic	nonsynony	TET2:NM_	NA	.	rs3678665
MALT_TH	TM_21	MALT_TH_14_1573	chr9:5462905_C>T	1	CD274	639	125	NA	766	83%	16%	NA	exonic	stopgain	CD274:NM	NA	.	.
MALT_TH	TM_22	MALT_TH_14_1617	chr1:26696917_C>T	2	ARID1A	140	41	NA	181	77%	23%	NA	exonic	stopgain	ARID1A:NM	NA	.	.
MALT_TH	TM_22	MALT_TH_14_1617	chr22:41093046_G>C	2	EP300	880	236	NA	1118	79%	21%	NA	exonic	nonsynony	EP300:NM	NA	.	.
MALT_TH	TM_22	MALT_TH_14_1617	chr19:16326855_G>A	2	KLF2	727	128	NA	856	85%	15%	NA	splicing	.	.	NA	.	.
MALT_TH	TM_22	MALT_TH_14_1617	chr4:105272735_C>T	3	TET2	460	163	NA	625	74%	26%	NA	exonic	stopgain	TET2:NM_	NA	.	.
MALT_TH	TM_22	MALT_TH_14_1617	chr6:167137305_CAGAC>-	1	CCR6	59	50	NA	110	54%	45%	NA	exonic	frameshift	CCR6:NM_	NA	.	.
MALT_TH	TM_22	MALT_TH_14_1617	chr9:5456116_G>T	3	CD274	18	60	NA	78	23%	77%	NA	exonic	nonsynony	CD274:NM	NA	.	.
MALT_TH	TM_23	MALT_TH_14_1791	chr4:105272823_C>-	1	TET2	116	20	NA	136	85%	15%	NA	exonic	frameshift	TET2:NM_	NA	.	.
MALT_TH	TM_24	MALT_TH_14_2	chr1:2561732_G>A	1	TNFRSF14	775	360	NA	1138	68%	32%	NA	exonic	stopgain	TNFRSF14:	NA	.	.
MALT_TH	TM_24	MALT_TH_14_2	chr11:118894610_C>T	1	CXCR5	881	233	NA	1116	79%	21%	NA	exonic	nonsynony	CXCR5:NM	NA	.	.
MALT_TH	TM_24	MALT_TH_14_2	chr1:26697037_->AC	1	ARID1A	245	111	NA	356	69%	31%	NA	exonic	frameshift	ARID1A:NM	NA	.	.
MALT_TH	TM_24	MALT_TH_14_2	chr4:105235468_C>G	1	TET2	804	951	NA	1761	46%	54%	NA	exonic	stopgain	TET2:NM_	NA	.	rs7714085
MALT_TH	TM_24	MALT_TH_14_2	chr6:137878848_TTCTG>-	1	TNFAIP3	434	83	NA	517	84%	16%	NA	exonic	frameshift	TNFAIP3:N	NA	.	.
MALT_TH	TM_24	MALT_TH_14_2	chr9:5465542_A>-	1	CD274	156	60	NA	216	72%	28%	NA	exonic	frameshift	CD274:NM	NA	.	.
MALT_TH	TM_25	MALT_TH_14_675	chr1:2557797_C>G	1	TNFRSF14	212	243	NA	457	46%	53%	NA	exonic	stopgain	TNFRSF14:	NA	.	.
MALT_TH	TM_25	MALT_TH_14_675	chr11:118894621_TC>-	2	CXCR5	940	76	NA	1016	93%	7%	NA	exonic	frameshift	CXCR5:NM	NA	.	.
MALT_TH	TM_25	MALT_TH_14_675	chr12:49047987_T>C	1	KMT2D	474	147	NA	622	76%	24%	NA	exonic	nonsynony	KMT2D:NM	NA	.	.
MALT_TH	TM_25	MALT_TH_14_675	chr4:105243597_A>T	1	TET2	110	66	NA	177	62%	37%	NA	exonic	stopgain	TET2:NM_	NA	.	.
MALT_TH	TM_25	MALT_TH_14_675	chr4:105259707_->GTAA	1	TET2	305	101	NA	406	75%	25%	NA	exonic	frameshift	TET2:NM_	NA	.	.
MALT_TH	TM_15	MALT_TH_13_574	chr4:102030199_C>T	1	BANK1	126	86	NA	212	59%	41%	NA	exonic	nonsynony	BANK1:NM	NA	.	.
MALT_TH	TM_26	MALT_TH_14_915	chr1:2559899_C>G	1	TNFRSF14	501	413	NA	916	55%	45%	NA	exonic	nonsynony	TNFRSF14:	NA	.	.
MALT_TH	TM_26	MALT_TH_14_915	chr12:49052914_C>A	1	KMT2D	760	223	NA	983	77%	23%	NA	splicing	.	.	NA	.	.
MALT_TH	TM_26	MALT_TH_14_915	chr14:102897288_->A	1	TRAF3	524	22	NA	546	96%	4%	NA	exonic	frameshift	TRAF3:NM	NA	.	.
MALT_TH	TM_26	MALT_TH_14_915	chr6:137877081_C>T	1	TNFAIP3	354	211	NA	566	63%	37%	NA	exonic	stopgain	TNFAIP3:N	NA	.	.
MALT_TH	TM_26	MALT_TH_14_915	chr9:5456157_T>A	1	CD274	36	68	NA	104	35%	65%	NA	exonic	stopgain	CD274:NM	NA	.	.

OM_18	.	.	.	.	.	.	.	.	0.926	P	0.335	B	0.674	N	1	N	2.015	M	.	.	-0.985	T	0.095	T	0.273	2.482	14.26
OM_19	.	.	.	.	.	.	.	.	.	.	.	.	.	.	.	.	.	.	.	.	.	.	.	.	.	.	.
OM_19	.	.	.	.	.	.	0.11	T	0.997	D	0.905	P	0.024	N	0.998	N	2.325	M	1.14	T	-0.923	T	0.171	T	0.515	1.968	12.54
OM_20	ID=COSM1	.	.	.	.	.	0.02	D	0.997	D	0.931	D	0	D	1	D	0.895	L	-0.87	T	-0.107	T	0.43	T	0.992	4.195	21.8
OM_21	ID=COSM3	.	.	.	.	.	0.04	D	.	.	.	.	0.003	N	1	A	.	.	.	.	.	.	.	.	.	4.264	22.2
OM_22	.	.	.	.	.	.	0	D	1	D	0.999	D	0	D	1	D	3.065	M	1.19	T	-0.337	T	0.284	T	0.715	3.15	16.53
OM_22	.	.	.	.	.	.	.	.	.	.	.	.	.	.	.	.	.	.	.	.	.	.	.	.	.	.	.
OM_22	.	.	.	.	.	.	0	D	0.999	D	0.731	P	0.002	U	1	D	2.39	M	0.54	T	-0.555	T	0.242	T	0.882	4.729	26.3
OM_23	.	.	.	.	.	.	.	.	.	.	.	.	.	.	.	.	.	.	.	.	.	.	.	.	.	.	.
OM_23	.	.	.	.	.	.	0	D	1	D	0.999	D	.	.	1	D	2.63	M	-1.1	T	0.47	D	0.669	D	0.879	5.208	33
OM_23	.	.	.	.	.	.	.	.	.	.	.	.	.	.	.	.	.	.	.	.	.	.	.	.	.	.	.
OM_24	.	.	.	.	.	.	0.94	T	.	.	.	.	0	D	1	A	.	.	.	.	.	.	.	.	.	4.331	22.8
OM_24	.	.	.	.	.	.	0.08	T	0.989	D	0.902	P	0	D	1	D	0.805	L	1.58	T	-1.07	T	0.074	T	0.354	4.245	22.1
OM_25	.	.	.	.	.	.	0	D	0.997	D	0.469	P	0.002	N	0.989	N	0	N	-1.37	T	-0.519	T	0.312	T	0.351	0.786	8.147
OM_25	.	.	.	.	.	.	0.07	T	0.825	P	0.466	P	0.924	U	0.997	N	3.35	M	1.18	T	-0.916	T	0.152	T	0.312	3.722	18.9
SM_1	.	.	.	.	.	.	0.01	D	0.976	D	0.726	P	.	.	1	D	1.79	L	3.06	T	-1.197	T	0.03	T	0.591	2.664	14.87
SM_2	.	.	.	.	.	.	0	D	0	B	0.001	B	.	.	1	N	0	N	-2.49	D	-0.746	T	0.35	T	0.714	0.56	7.028
SM_2	.	.	.	.	.	.	.	.	.	.	.	.	.	.	1	D	.	.	.	.	.	.	.	.	.	4.031	20.7
SM_2	.	.	.	.	.	.	0.11	T	1	D	0.983	D	0	D	1	D	2.075	M	-0.41	T	-0.242	T	0.417	T	0.744	3.677	18.68
SM_2	.	.	.	.	.	.	0.01	D	0.988	D	0.988	D	0	D	1	D	3.81	H	-5.86	D	1.084	D	0.985	D	0.931	3.252	16.91
SM_2	.	.	.	.	.	.	.	.	.	.	.	.	.	.	.	.	.	.	.	.	.	.	.	.	.	.	.
SM_3	.	.	.	.	.	.	0	D	0.999	D	0.959	D	0.002	N	1	D	3.17	M	1.22	T	-0.256	T	0.299	T	0.847	4.351	22.9
SM_3	.	.	.	.	.	.	.	.	.	.	.	.	.	.	.	.	.	.	.	.	.	.	.	.	.	.	.
SM_4	.	.	.	.	.	.	0.21	T	0.999	D	0.99	D	0	D	1	D	3.22	M	-1.44	T	0.515	D	0.723	D	0.934	4.177	21.6
SM_4	.	.	.	.	.	.	.	.	.	.	.	.	.	.	.	.	.	.	.	.	.	.	.	.	.	.	.
SM_4	.	.	.	.	.	.	.	.	.	.	.	.	.	.	.	.	.	.	.	.	.	.	.	.	.	.	.
SM_4	.	.	.	.	.	.	0.26	T	.	.	.	.	0.002	N	1	D	.	.	.	.	.	.	.	.	.	5.504	35
SM_5	.	.	.	.	.	.	1	T	0.857	P	0.498	P	0	D	1	D	0.385	N	0.31	T	-0.946	T	0.121	T	0.768	3.847	19.54
SM_5	.	.	.	.	.	.	0	D	0.976	D	0.817	P	0	D	1	D	3.48	M	-1.47	T	0.651	D	0.728	D	0.975	4.838	27.5
SM_6	.	.	.	.	.	.	0	D	0.958	D	0.609	P	0.001	N	0.928	D	1.04	L	-1.5	D	-0.036	T	0.418	T	0.427	0.946	8.856
SM_6	.	.	.	.	.	.	.	.	.	.	.	.	.	.	.	.	.	.	.	.	.	.	.	.	.	.	.
SM_6	.	.	.	.	.	.	0	D	1	D	0.988	D	0.009	N	0.621	D	.	.	.	.	-0.03	T	0.447	T	0.46	4.805	27.1
SM_7	.	.	.	.	.	.	.	.	.	.	.	.	.	.	.	.	.	.	.	.	.	.	.	.	.	.	.
SM_7	.	.	.	.	.	.	0.02	D	0.022	B	0.001	B	.	.	1	N	0.975	L	2.5	T	-1	T	0.024	T	0.309	2.666	14.88
SM_8	.	.	.	.	.	.	0.12	T	.	.	.	.	0	D	1	A	.	.	.	.	.	.	.	.	.	5.45	35
SM_9	.	.	.	.	.	.	0	D	1	D	0.991	D	0	D	1	D	3.43	M	-0.85	T	0.588	D	0.685	D	0.954	4.967	29
SM_10	.	.	.	.	.	.	0.02	D	0.996	D	0.522	P	0.905	U	1	N	0	N	1.18	T	-1.013	T	0.051	T	0.137	0.707	7.771
SFM_1	.	.	.	.	.	.	0.2	T	.	.	.	.	0	D	1	D	.	.	.	.	.	.	.	.	.	8.576	41
SFM_1	.	.	.	.	.	.	0.04	D	0.999	D	0.912	D	0	D	1	D	1.67	L	.	.	-0.477	T	0.322	T	0.686	4.43	23.6
TM_1	.	not provid	Familial_ca	RCV00004	MedGen:C	C0346153:	0.01	D	0.998	D	0.87	P	0.002	N	1	N	2.005	M	5.26	T	-1.087	T	0.013	T	0.756	1.239	10.03
TM_1	.	.	.	.	.	.	.	.	.	.	.	.	.	.	.	.	.	.	.	.	.	.	.	.	.	.	.
TM_1	.	.	.	.	.	.	0.42	T	.	.	.	.	0.993	U	1	D	.	.	.	.	.	.	.	.	.	7.45	39
TM_2	.	.	.	.	.	.	1	T	.	.	.	.	.	.	1	D	.	.	.	.	.	.	.	.	.	14.638	48
TM_2	.	.	.	.	.	.	.	.	.	.	.	.	.	.	.	.	.	.	.	.	.	.	.	.	.	.	.
TM_3	.	.	.	.	.	.	0.03	D	0.998	D	0.993	D	0	D	1	D	2.56	M	3.13	T	-1.105	T	0.08	T	0.828	5.225	33
TM_3	ID=COSM4	.	.	.	.	.	1	T	.	.	.	.	.	.	1	D	.	.	.	.	.	.	.	.	.	14.765	48
TM_4	.	.	.	.	.	.	.	.	.	.	.	.	.	.	.	.	.	.	.	.	.	.	.	.	.	.	.
TM_4	.	.	.	.	.	.	0.63	T	0.991	D	0.978	D	0	D	1	D	1.825	L	1.51	T	-0.945	T	0.144	T	0.355	2.853	15.5
TM_4	.	.	.	.	.	.	0.71	T	0.179	B	0.023	B	.	.	1	N	0.345	N	-0.08	T	-1.01	T	0.08	T	0.424	0.985	9.02
TM_4	.	.	.	.	.	.	.	.	.	.	.	.	.	.	.	.	.	.	.	.	.	.	.	.	.	.	.
TM_4	.	.	.	.	.	.	0	D	1	D	0.999	D	0	D	1	D	3.005	M	1.38	T	-0.525	T	0.251	T	0.967	5.183	32
TM_5	.	.	.	.	.	.	0.01	D	0.999	D	0.965	D	0	D	1	D	0.805	L	1.46	T	-1.01	T	0.097	T	0.088	5.382	34
TM_5	.	.	.	.	.	.	0.56	T	.	.	.	.	.	.	1	A	.	.	.	.	.	.	.	.	.	4.545	24.6
TM_5	.	.	.	.	.	.	.	.	.	.	.	.	.	.	.	.	.	.	.	.	.	.	.	.	.	.	.
TM_5	.	.	.	.	.	.	.	.	.	.	.	.	.	.	.	.	.	.	.	.	.	.	.	.	.	.	.
TM_5	.	.	.	.	.	.	.	.	.	.	.	.	.	.	1	D	.	.	.	.	.	.	.	.	.	2.98	15.94
TM_6	.	.	.	.	.	.	0.54	T	.	.	.	.	.	.	1	A	.	.	.	.	.	.	.	.	.	3.653	18.57
TM_6	.	.	.	.	.	.	0.04	D	0.979	D	0.579	P	0	D	1	D	1.355	L	-0.19	T	-0.543	T	0.255	T	0.723	2.087	12.94
TM_6	.	.	.	.	.	.	.	.	.	.	.	.	.	.	.	.	.	.	.	.	.	.	.	.	.	.	.
TM_6	.	.	.	.	.	.	1	T	.	.	.	.	0	D	1	A	.	.	.	.	.	.	.	.	.	6.721	38
TM_7	.	Pathogenic	Familial_ca	RCV00004	MedGen:C	C0346153:	.	.	.	.	.	.	.	.	.	.	.	.	.	.	.	.	.	.	.	.	.
TM_8	ID=COSM3	.	.	.	.	.	0	D	1	D	1	D	0	D	1	D	3.255	M	-7.57	D	0.903	D	0.995	D	0.917	4.681	25.9
TM_8	.	.	.	.	.	.	0.52	T	.	.	.	.	0	N	1	D	.	.	.	.	.	.	.	.	.	4.987	29.2
TM_8	.	.	.	.	.	.	.	.	.	.	.	.	.	.	.	.	.	.	.	.	.	.	.	.	.	.	.
TM_9	.	.	.	.	.	.	0.03	D	1	D	0.999	D	.	.	1	D	2.105	M	-0.59	T	0.04	D	0.504	D	0.989	4.596	25
TM_9	ID=COSM9	.	.	.	.	.	0	D	1	D	1	D	0	D	1	D	2.455	M	-6.64	D	1	D	0.988	D	0.86	3.724	18.91
TM_9	.	.	.	.	.	.	0.17	T	.	.	.	.	0	N	1	D	.	.	.	.	.	.	.	.	.	8.903	41
TM_9	.	.	.	.	.	.	.	.	.	.	.	.	.	.	.	.	.	.	.	.	.	.	.	.	.	.	.
TM_9	.	.	.	.	.	.	0.01	D	0.814	P	0.772	P	0.138	N	0.996	N	2.24	M	-1.2	T	-0.452	T	0.482	T	0.583	1.676	11.56
TM_10	.	.	.	.	.	.	0	D	1	D	0.997	D	.	.	1	N	2.74	M	-0.09	T	-0.555	T	0.447	T	0.891	2.698	14.98
TM_10	.	.	.	.	.	.	.	.	.	.	.	.	.	.	1	D	.	.	.	.	.	.	.	.	.	4.789	27
TM_10	.	.	.	.	.	.	0	D	.	.	.	.	0	U	1	A	.	.	.	.	.	.	.	.	.	6.549	37
TM_10	.	.	.	.	.	.	.	.	.	.	.	.	.	.	1	D	.	.	.	.	.	.	.	.	.	1.171	9.763
TM_10	.	.	.	.	.	.	0	D	1	D	0.999	D	0	D	1	D	2.075	M	-0.23	T	-0.044	T	0.461	T	0.986	4.76	26.7
TM_10	.	.	.	.	.	.	0	D	1	D	1	D	.	.	1	D	2.82	M	0.99	T	-0.309	T	0.314	T	0.967	4.652	25.6
TM_10	.	.	.	.	.	.	0.27	T	.	.	.	.	.	.	1	D	.	.	.	.	.	.	.	.	.	15.545	49
TM_10	.	.	.	.	.	.	0.02	D	0.993	D	0.881	P	0.002	N	0.567	N	1.845	L	5.08	T	-0.95	T	0.123	T	0.502	3.359	17.32
TM_26	.	.	.	.	.	.	0.09	T	0.998	D	0.981	D	.	.	1	D	3.39	M	1.04	T	-0.373	T	0.263	T	0.84	4.75	26.6
TM_11	.	.	.	.	.	.	1	T	0.604	P	0.252	B	0	D	1	D	0.64	N	-0.17	T	-0.904	T	0.149	T	0.795	1.888	12.27
TM_11	.	.	.	.	.	.	0.15	T	0.999	D	0.972	D	0.041	N	0.668	N	2.185	M	1.05	T	-0.848	T	0.19	T	0.409	2.377	13.91
TM_11	.	.	.	.	.	.	.	.	.	.	.	.	.	.	.	.	.	.	.	.	.	.	.	.	.	.	.
TM_11	.	.	.	.	.	.	.	.	.	.	.	.	.	.	.	.	.	.	.	.	.	.	.	.	.	.	.
TM_12	.	.	.	.	.	.	1	T	.	.	.	.	.	.	1	A	.	.	.	.	.	.	.	.	.	4.957	28.9
TM_12	ID=COSM2	.	.	.	.	.	1	T	.	.	.	.	0.454	U	1	D	.	.	.	.	.	.	.	.	.	4.849	27.6
TM_12	.	.	.	.	.	.	0.11	T	0.967	D	0.7	P	.	.	1	N	0.85	L	-3.01	D	-0.185	T	0.65	D	0.157	2.887	15.62
TM_12	.	.	.	.	.	.	0.01	D	1	D	0.985	D	0.062	N	0.876	D	1.975	M	1.16	T	-0.727	T	0.171	T	0.457	2.882	15.6
TM_12	.	.	.	.	.	.	1	T	.	.	.	.	.	.	1	D	.	.	.	.	.	.	.	.	.	12.107	46
TM_12	.	.	.	.	.	.	0.12	T	0.998	D	0.883	P	0	D	0.989	D	1.39	L	-1.68	D	0.176	D	0.618	D	0.461	3.242	16.87
TM_12	.	.	.	.	.	.	0	D	1	D	0.999	D	.	.	1	N	-0.26	N	0.75	T	-0.921	T	0.121	T	0.407	2.616	14.71
TM_12	ID=COSM1	.	.	.	.	.	1	T	.	.	.	.	.	.	1	D	.	.	.	.	.	.	.	.	.	15.608	49
TM_12	.	.	.	.	.	.	0.11	T	1	D	0.998	D	0	D	1	D	2.835	M	4.21	T	-1.183	T	0.038	T	0.616	3.122	16.43
TM_12	.	.	.	.	.	.	0.01	D	.	.	.	.	.	.	1	D	.	.	.	.	-0.306	T	0.369	T	.	5.998	37
TM_13	.	.	.	.	.	.	0.14	T	.	.	.	.	0	D	1	D	.	.	.	.	.	.	.	.	.	11.392	45
TM_49	ID=COSM1	.	.	.	.	.	0.28	T	0.994	D	0.903	P	.	.	1	N	1.85	L	0.98	T	-1.059	T	0.097	T	0.353	1.986	12.6
TM_13	.	.	.	.	.	.	0.05	D	0.745	P	0.301	B	0.002	N	1	N	1.61	L	1.4	T	-1.024	T	0.034	T	0.294	3.536	18.05
TM_13	.	.	.	.	.	.	0.02	D	1	D	0.999	D	0	D	1	D	3.115	M	-1.57	D	0.695	D	0.779	D	0.975	4.817	27.3
TM_13	.	.	.	.	.	.	.	.	.	.	.	.	.	.	.	.	.	.	.	.	.	.	.	.	.	.	.
TM_13	ID=COSM3	.	.	.	.	.	.	.	.	.	.	.	.	.	.	.	.	.	.	.	.	.	.	.	.	.	.
TM_13	.	.	.	.	.	.	0	D	0.993	D	0.971	D	0	D	0.926	N	.	.	5.21	T	-0.828	T	0.214	T	0.932	2.429	14.08
TM_14	.	.	.	.	.	.	.	.	.	.	.	.	.	.	.	.	.	.	.	.	.	.	.	.	.	.	.
TM_14	.	.	.	.	.	.	0.01	D	1	D	0.91	D	0.07	N	1	N	2.085	M	-0.73	T	-0.648	T	0.407	T	0.05	1.735	11.76
TM_14	.	.	.	.	.	.	0.97	T	0.9	P	0.262	B	.	.	0.994	N	1.24	L	3.14	T	-1.012	T	0.012	T	0.184	-0.791	0.7
TM_14	.	.	.	.	.	.	0	D	1	D	0.999	D	.	.	1	N	-0.26	N	0.75	T	-0.921	T	0.121	T	0.407	2.616	14.71
TM_14	.	.	.	.	.	.	0.39	T	1	D	0.995	D	0	D	1	D	1.795	L	1.25	T	-0.939	T	0.205	T	0.717	4.843	27.6
TM_14	ID=COSM8	.	.	.	.	.	0	D	1	D	0.993	D	.	.	1	D	2.565	M	0.8	T	-1.181	T	0.058	T	0.966	3.936	20.1
TM_14	.	.	.	.	.	.	1	T	.	.	.	.	.	.	1	D	.	.	.	.	.	.	.	.	.	15.487	49
TM_14	.	.	.	.	.	.	.	.	.	.	.	.	.	.	0.907	D	.	.	.	.	.	.	.	.	.	0.927	8.778
TM_14	.	.	.	.	.	.	0	D	1	D	0.984	D	0	D	1	D	3.385	M	-4.28	D	1.088	D	0.955	D	0.92	3.584	18.26
TM_15	.	.	.	.	.	.	.	.	.	.	.	.	.	.	.	.	.	.	.	.	.	.	.	.	.	.	.
TM_11	ID=COSM1	.	.	.	.	.	.	.	0.049	B	0.022	B	0.025	N	0.998	N	1.95	M	-0.86	T	-0.838	T	0.271	T	0.209	1.297	10.24
TM_15	.	.	.	.	.	.	.	.	.	.	.	.	.	.	.	.	.	.	.	.	.	.	.	.	.	.	.
TM_15	.	.	.	.	.	.	1	T	.	.	.	.	.	.	1	D	.	.	.	.	.	.	.	.	.	16.487	50
TM_16	.	.	.	.	.	.	0.04	D	1	D	0.972	D	0	D	1	D	2.495	M	2.47	T	-0.691	T	0.199	T	0.645	4.809	27.2
TM_17	.	.	.	.	.	.	.	.	.	.	.	.	.	.	.	.	.	.	.	.	.	.	.	.	.	.	.
TM_17	.	.	.	.	.	.	.	.	.	.	.	.	.	.	.	.	.	.	.	.	.	.	.	.	.	.	.
TM_18	.	.	.	.	.	.	0.53	T	0.914	P	0.2	B	.	.	1	N	0.695	N	-2.13	D	-0.712	T	0.32	T	0.516	0.181	4.977
TM_18	.	.	.	.	.	.	0	D	1	D	0.999	D	.	.	1	N	-0.26	N	0.75	T	-0.921	T	0.121	T	0.407	2.616	14.71
TM_18	ID=COSM2	.	.	.	.	.	0.01	D	1	D	0.999	D	.	.	1	D	2.865	M	2.09	T	-0.662	T	0.183	T	0.947	3.575	18.22
TM_47	.	.	.	.	.	.	0.42	T	1	D	0.989	D	0	D	1	D	1.545	L	-1.23	T	-0.143	T	0.526	D	0.791	4.527	24.4
TM_18	.	.	.	.	.	.	0.27	T	.	.	.	.	0	D	1	D	.	.	.	.	.	.	.	.	.	6.55	37
TM_19	.	.	.	.	.	.	.	.	.	.	.	.	.	.	.	.	.	.	.	.	.	.	.	.	.	.	.
TM_19	.	.	.	.	.	.	.	.	.	.	.	.	.	.	.	.	.	.	.	.	.	.	.	.	.	.	.
TM_19	.	.	.	.	.	.	0.02	D	0.979	D	0.681	P	0.019	N	0.963	D	1.445	L	5.42	T	-0.699	T	0.007	T	0.641	2.54	14.45
TM_13	.	.	.	.	.	.	0.01	D	0.999	D	0.992	D	0.011	N	1	N	1.15	L	2.71	T	-1.072	T	0.118	T	0.362	2.653	14.83
TM_19	.	.	.	.	.	.	.	.	.	.	.	.	.	.	.	.	.	.	.	.	.	.	.	.	.	.	.
TM_19	.	.	.	.	.	.	0	D	0.999	D	0.982	D	0.051	N	0.973	N	2.395	M	-1.24	T	-0.045	T	0.619	D	0.741	2.882	15.6
TM_20	.	.	.	.	.	.	0	D	0.924	P	0.827	P	.	.	1	D	.	.	-2.2	D	0.303	D	0.713	D	0.828	2.591	14.62
TM_20	.	.	.	.	.	.	.	.	.	.	.	.	.	.	.	.	.	.	.	.	.	.	.	.	.	.	.
TM_20	ID=COSM2	.	.	.	.	.	0	D	1	D	0.999	D	.	.	1	D	2.865	M	2.02	T	-0.653	T	0.184	T	0.952	3.414	17.54
TM_21	.	.	.	.	.	.	0.04	D	.	.	.	.	0.089	N	1	D	.	.	.	.	.	.	.	.	.	10.634	44
TM_21	.	.	.	.	.	.	0.76	T	0.546	P	0.621	P	.	.	1	D	-0.625	N	1.21	T	-1.075	T	0.047	T	0.091	2.21	13.35
TM_21	.	.	.	.	.	.	0	D	.	.	.	.	0	D	1	A	.	.	.	.	.	.	.	.	.	5.661	36
TM_22	.	.	.	.	.	.	0.27	T	.	.	.	.	0	U	1	D	.	.	.	.	.	.	.	.	.	4.164	21.5
TM_22	.	.	.	.	.	.	0	D	0.999	D	0.972	D	0.022	N	1	D	1.965	M	-2.77	D	0.783	D	0.81	D	0.173	5.107	32
TM_22	.	.	.	.	.	.	.	.	.	.	.	.	.	.	1	D	.	.	.	.	.	.	.	.	.	3.497	17.88
TM_22	ID=COSM4	.	.	.	.	.	1	T	.	.	.	.	.	.	1	D	.	.	.	.	.	.	.	.	.	14.765	48
TM_22	.	.	.	.	.	.	.	.	.	.	.	.	.	.	.	.	.	.	.	.	.	.	.	.	.	.	.
TM_22	.	.	.	.	.	.	0	D	0.993	D	0.971	D	0	D	0.937	N	.	.	5.21	T	-0.828	T	0.214	T	0.932	2.369	13.88
TM_23	.	.	.	.	.	.	.	.	.	.	.	.	.	.	.	.	.	.	.	.	.	.	.	.	.	.	.
TM_24	.	.	.	.	.	.	0.21	T	.	.	.	.	.	.	1	A	.	.	.	.	.	.	.	.	.	2.665	14.87
TM_24	.	.	.	.	.	.	0.08	T	0.999	D	0.799	P	0.42	U	1	D	1.905	M	1.12	T	-0.761	T	0.199	T	0.535	3.909	19.88
TM_24	.	.	.	.	.	.	.	.	.	.	.	.	.	.	.	.	.	.	.	.	.	.	.	.	.	.	.
TM_24	ID=COSM1	.	.	.	.	.	0.05	D	.	.	.	.	.	.	1	D	.	.	.	.	.	.	.	.	.	5.87	36
TM_24	.	.	.	.	.	.	.	.	.	.	.	.	.	.	.	.	.	.	.	.	.	.	.	.	.	.	.
TM_24	.	.	.	.	.	.	.	.	.	.	.	.	.	.	.	.	.	.	.	.	.	.	.	.	.	.	.
TM_25	.	.	.	.	.	.	1	T	.	.	.	.	.	.	1	A	.	.	.	.	.	.	.	.	.	3.628	18.46
TM_25	.	.	.	.	.	.	.	.	.	.	.	.	.	.	.	.	.	.	.	.	.	.	.	.	.	.	.
TM_25	.	.	.	.	.	.	0	D	1	D	0.998	D	0.001	N	1	D	2.35	M	-5.32	D	1.097	D	0.973	D	0.917	2.925	15.75
TM_25	ID=COSM2	.	.	.	.	.	0	D	.	.	.	.	.	.	1	D	.	.	3.78	T	-1.213	T	0.043	T	.	14.635	48
TM_25	.	.	.	.	.	.	.	.	.	.	.	.	.	.	.	.	.	.	.	.	.	.	.	.	.	.	.
TM_15	.	.	.	.	.	.	0.21	T	1	D	1	D	0	D	0.994	D	1.935	M	0.6	T	-0.594	T	0.317	T	0.591	4.328	22.7
TM_26	.	.	.	.	.	.	0	D	1	D	0.999	D	.	.	0.994	D	2.975	M	-0.08	T	-0.353	T	0.406	T	0.602	2.372	13.89
TM_26	.	.	.	.	.	.	.	.	.	.	.	.	.	.	1	D	.	.	.	.	.	.	.	.	.	2.968	15.89
TM_26	ID=COSM2	.	.	.	.	.	.	.	.	.	.	.	.	.	.	.	.	.	.	.	.	.	.	.	.	.	.
TM_26	ID=COSM3	Pathogenic	Autoinflam	RCV00020	MedGen:C	CN234876	1	T	.	.	.	.	0	D	1	A	.	.	.	.	.	.	.	.	.	6.29	37
TM_26	.	.	.	.	.	.	0.25	T	.	.	.	.	0.438	N	1	A	.	.	.	.	.	.	.	.	.	6.761	38

MALT_OA	OM_18	MALT_OA_chr18:6236	1	TNFRSF11/	698	724	NA	-878.491	-153774%	-219698%	NA	exonic	nonsynony	TNFRSF11/	NA	.	rs748211485
MALT_OA	OM_19	MALT_OA_chr3:17705	1	TBL1XR2	769	1066	NA	-430.27	-75341%	-107654%	NA	exonic	stopgain	TBL1XR1:N	NA	.	.
MALT_OA	OM_19	MALT_OA_chr5:76733	1	F2R	203	187	NA	-303.856	-53212%	-76038%	NA	exonic	nonsynony	F2R:NM_0	NA	.	rs144447562
MALT_OA	OM_20	MALT_OA_chr3:18355	1	KLHL7	38	1	NA	-123.932	-21723%	-31053%	NA	exonic	nonsynony	KLHL6:NM	NA	.	rs749398006
MALT_OA	OM_21	MALT_OA_chr6:13787	1	TNFAIP4	123	149	NA	-111.761	-19597%	-28019%	NA	exonic	stopgain	TNFAIP3:N	NA	.	.
MALT_OA	OM_22	MALT_OA_chr17:4055	1	CCR8	298	287	NA	-418.509	-73276%	-104702%	NA	exonic	nonsynony	CCR7:NM_	NA	.	.
MALT_OA	OM_22	MALT_OA_chr16:3728	1	CREBBP	74	68	NA	-109.851	-19261%	-27537%	NA	exonic	nonframes	CREBBP:NI	NA	.	rs778678603
MALT_OA	OM_22	MALT_OA_chr1:11991	1	NOTCH3	74	72	NA	-102.512	-17977%	-25702%	NA	exonic	nonsynony	NOTCH2:N	NA	.	rs746551843
MALT_OA	OM_23	MALT_OA_chr6:13787	1	TNFAIP4	-120	-506	NA	-611.377	-107023%	-152909%	NA	exonic	frameshift	TNFAIP3:N	NA	.	.
MALT_OA	OM_23	MALT_OA_chrX:40055	1	BCOR	345	-107	NA	-1366.61	-239192%	-341722%	NA	exonic	nonsynony	BCOR:NM_	NA	.	.
MALT_OA	OM_23	MALT_OA_chr6:13787	1	TNFAIP3	-226	-592	NA	-430.117	-75301%	-107590%	NA	exonic	frameshift	TNFAIP3:N	NA	.	.
MALT_OA	OM_24	MALT_OA_chr6:13787	1	TNFAIP3	-345	-739	NA	-328.235	-57468%	-82114%	NA	exonic	stopgain	TNFAIP3:N	NA	.	.
MALT_OA	OM_24	MALT_OA_chr6:13787	1	TNFAIP3	-185	-416	NA	-215.526	-37745%	-53938%	NA	exonic	nonsynony	TNFAIP3:N	NA	.	.
MALT_OA	OM_25	MALT_OA_chr12:4904	1	KMT2D	249	241	NA	-347.843	-60910%	-87035%	NA	exonic	nonsynony	KMT2D:NM	NA	.	rs760263014
MALT_OA	OM_25	MALT_OA_chr3:46373	1	CCR6	199	185	NA	-292.516	-51227%	-73203%	NA	exonic	nonsynony	CCR5:NM_	NA	.	rs765009973
MALT_SA	SM_1	MALT_SA_chr6:10608	2	PRDM2	22	-41	NA	-157.314	-27563%	-39395%	NA	exonic	nonsynony	PRDM1:NM	TRUE	.	rs756156242
MALT_SA	SM_2	MALT_SA_chr1:25617	1	TNFRSF15	722	705	NA	-997.841	-174659%	-249535%	NA	exonic	nonsynony	TNFRSF14:	NA	.	.
MALT_SA	SM_2	MALT_SA_chr12:4904	1	KMT2D	-202	-613	NA	-553.416	-96879%	-138416%	NA	splicing	.	.	NA	.	.
MALT_SA	SM_2	MALT_SA_chr11:1083	1	ATM	58	-31	NA	-255.953	-44826%	-64057%	NA	exonic	nonsynony	ATM:NM_(	NA	.	rs753389617
MALT_SA	SM_2	MALT_SA_chr16:3738	1	CREBBP	-300	-890	NA	-784.089	-137247%	-196084%	NA	exonic	nonsynony	CREBBP:NI	NA	.	.
MALT_SA	SM_2	MALT_SA_chr3:17705	1	TBL1XR2	620	790	NA	-485.717	-85041%	-121510%	NA	exonic	frameshift	TBL1XR1:N	NA	.	.
MALT_SA	SM_3	MALT_SA_chr3:46204	1	CCR2	252	93	NA	-655.917	-114821%	-164050%	NA	exonic	nonsynony	CCR1:NM_	NA	.	.
MALT_SA	SM_3	MALT_SA_chr6:13787	1	TNFAIP4	-1474	-3058	NA	-1202.27	-210424%	-300621%	NA	exonic	frameshift	TNFAIP3:N	NA	.	.
MALT_SA	SM_4	MALT_SA_chr3:17703	1	TBL1XR2	-122	-307	NA	-207.466	-36336%	-51926%	NA	exonic	nonsynony	TBL1XR1:N	NA	.	.
MALT_SA	SM_4	MALT_SA_chr4:10523	2	TET3	548	632	NA	-561.784	-98351%	-140523%	NA	exonic	frameshift	TET2:NM_	NA	.	rs759055582
MALT_SA	SM_4	MALT_SA_chr7:29398	1	CARD12	-257	-561	NA	-264.882	-46382%	-66276%	NA	exonic	nonframes	CARD11:NI	NA	.	.
MALT_SA	SM_4	MALT_SA_chrX:41696	1	GPR35	-24	-74	NA	-68.7564	-12063%	-17251%	NA	exonic	stopgain	GPR34:NM	NA	.	.
MALT_SA	SM_5	MALT_SA_chr3:17704	2	TBL1XR1	-200	-491	NA	-316.143	-55355%	-79095%	NA	exonic	nonsynony	TBL1XR1:N	TRUE	.	.
MALT_SA	SM_5	MALT_SA_chr3:17704	2	TBL1XR1	-496	-1095	NA	-536.873	-93981%	-134274%	NA	exonic	nonsynony	TBL1XR1:N	TRUE	.	.
MALT_SA	SM_6	MALT_SA_chr12:4904	2	KMT2D	229	271	NA	-223.783	-39201%	-56023%	NA	exonic	nonsynony	KMT2D:NM	TRUE	.	rs199845252
MALT_SA	SM_6	MALT_SA_chr3:17704	2	TBL1XR2	-569	-1226	NA	-554.894	-97134%	-138779%	NA	exonic	nonframes	TBL1XR1:N	TRUE	.	.
MALT_SA	SM_6	MALT_SA_chr17:4528	2	MAP3K15	435	476	NA	-498.475	-87271%	-124695%	NA	exonic	nonsynony	MAP3K14:	TRUE	.	.
MALT_SA	SM_7	MALT_SA_chr14:1029	1	TRAF4	-6	-325	NA	-629.335	-110167%	-157400%	NA	exonic	frameshift	TRAF3:NM	NA	.	.
MALT_SA	SM_7	MALT_SA_chr14:1002	1	YY2	-347	-839	NA	-526.153	-92106%	-131597%	NA	exonic	nonsynony	YY1:NM_0	NA	.	.
MALT_SA	SM_8	MALT_SA_chr6:13787	2	TNFAIP4	-736	-1797	NA	-1141.48	-199788%	-285429%	NA	exonic	stopgain	TNFAIP3:N	TRUE	.	.
MALT_SA	SM_9	MALT_SA_chr3:17705	2	TBL1XR2	50	67	NA	-31.6599	-5582%	-7999%	NA	exonic	nonsynony	TBL1XR1:N	TRUE	.	.
MALT_SA	SM_10	MALT_SA_chrX:1466	2	P2RY9	148	-10	NA	-517.943	-90675%	-129555%	NA	exonic	nonsynony	P2RY8:NM	TRUE	.	.
MALT_SF	SFM_1	MALT_SF_chr1:85267	1	BCL10	-374	-805	NA	-363.563	-63651%	-90946%	NA	exonic	stopgain	BCL10:NM	NA	.	.
MALT_SF	SFM_1	MALT_SF_chr1:85267	1	BCL10	-276	-649	NA	-377.496	-66091%	-94432%	NA	exonic	nonsynony	BCL10:NM	NA	.	.
MALT_TH	TM_1	MALT_TH_chr13:3233	1	BRCA3	-730	-1562	NA	-690.235	-120819%	-172614%	NA	exonic	nonsynony	BRCA2:NM	NA	.	rs80358564
MALT_TH	TM_1	MALT_TH_chr12:4902	1	KMT2D	-849	-1944	NA	-1057.51	-185093%	-264436%	NA	exonic	stopgain	KMT2D:NM	NA	.	.
MALT_TH	TM_1	MALT_TH_chr4:10523	1	TET2	-1009	-2206	NA	-1048.21	-183465%	-262109%	NA	exonic	stopgain	TET2:NM_	NA	.	rs754287181
MALT_TH	TM_2	MALT_TH_chr4:10527	1	TET2	-78	-170	NA	-80.2204	-14066%	-20110%	NA	exonic	stopgain	TET2:NM_	NA	.	.
MALT_TH	TM_2	MALT_TH_chr6:13787	1	TNFAIP4	-1057	-2574	NA	-1624.14	-284255%	-406096%	NA	exonic	frameshift	TNFAIP3:N	NA	.	.
MALT_TH	TM_3	MALT_TH_chr3:3814(	1	MYD89	287	310	NA	-335.811	-58805%	-84029%	NA	exonic	nonsynony	MYD88:NM	NA	.	.
MALT_TH	TM_3	MALT_TH_chr4:10527	3	TET3	-225	-497	NA	-244.206	-42764%	-61108%	NA	exonic	stopgain	TET2:NM_	NA	.	.
MALT_TH	TM_4	MALT_TH_chr19:4187	1	CD79A	-3613	-7320	NA	-2596.31	-454380%	-649129%	NA	exonic	frameshift	CD79A:NM	NA	.	.
MALT_TH	TM_4	MALT_TH_chr5:1510(	1	TNIP2	927	857	NA	-1383.86	-242212%	-346038%	NA	exonic	nonsynony	TNIP1:NM_	NA	.	.
MALT_TH	TM_4	MALT_TH_chr1:25599	1	TNFRSF15	-863	-2620	NA	-2374.75	-415613%	-593750%	NA	exonic	nonsynony	TNFRSF14:	NA	.	.
MALT_TH	TM_4	MALT_TH_chr4:10527	1	TET3	128	40	NA	-345.918	-60571%	-86550%	NA	exonic	frameshift	TET2:NM_	NA	.	.
MALT_TH	TM_4	MALT_TH_chr6:13787	1	TNFAIP4	-721	-1509	NA	-616.262	-107873%	-154119%	NA	exonic	nonsynony	TNFAIP3:N	NA	.	.
MALT_TH	TM_5	MALT_TH_chr6:11138	1	REV3L	-292	-643	NA	-313.543	-54898%	-78442%	NA	exonic	nonsynony	REV3L:NM	NA	.	.
MALT_TH	TM_5	MALT_TH_chr1:25599	1	TNFRSF15	-160	-662	NA	-790.045	-138290%	-197576%	NA	exonic	stopgain	TNFRSF14:	NA	.	.
MALT_TH	TM_5	MALT_TH_chr11:1188	3	CXCR6	-1091	-2214	NA	-790.973	-138446%	-197795%	NA	exonic	frameshift	CXCR5:NM	NA	.	.
MALT_TH	TM_5	MALT_TH_chr4:10525	1	TET3	-68	-232	NA	-236.73	-41460%	-59246%	NA	exonic	frameshift	TET2:NM_	NA	.	.
MALT_TH	TM_5	MALT_TH_chr9:54561	1	CD275	80	104	NA	-57.697	-10138%	-14506%	NA	splicing	.	.	NA	.	.
MALT_TH	TM_6	MALT_TH_chr1:25584	1	TNFRSF15	-291	-704	NA	-439.483	-76939%	-109930%	NA	exonic	stopgain	TNFRSF14:	NA	.	.
MALT_TH	TM_6	MALT_TH_chr3:18349	1	KLHL7	-168	-401	NA	-241.488	-42290%	-60431%	NA	exonic	nonsynony	KLHL6:NM	NA	.	.
MALT_TH	TM_6	MALT_TH_chr4:10527	1	TET3	-668	-1526	NA	-824.847	-144377%	-206269%	NA	exonic	frameshift	TET2:NM_	NA	.	.
MALT_TH	TM_6	MALT_TH_chr9:54629	1	CD275	-115	-517	NA	-650.707	-113906%	-162741%	NA	exonic	stopgain	CD274:NM	NA	.	.
MALT_TH	TM_7	MALT_TH_chr13:3233	1	BRCA3	35	33	NA	-49.8465	-8760%	-12536%	NA	exonic	frameshift	BRCA2:NM	NA	.	rs763069722
MALT_TH	TM_8	MALT_TH_chr17:7674	1	TP54	-1434	-3184	NA	-1612.21	-282164%	-403108%	NA	exonic	nonsynony	TP53:NM_	NA	.	.
MALT_TH	TM_8	MALT_TH_chr4:10523	1	TET3	-300	-732	NA	-463.477	-81138%	-115929%	NA	exonic	stopgain	TET2:NM_	NA	.	.
MALT_TH	TM_8	MALT_TH_chrX:7161(	1	CXCR4	-363	-760	NA	-309.594	-54206%	-77452%	NA	exonic	frameshift	CXCR3:NM	NA	.	.
MALT_TH	TM_9	MALT_TH_chrX:40062	1	BCOR	-483	-1070	NA	-530.87	-92931%	-132774%	NA	exonic	nonsynony	BCOR:NM_	NA	.	.
MALT_TH	TM_9	MALT_TH_chr17:7675	1	TP54	-1052	-2237	NA	-966.908	-169236%	-241782%	NA	exonic	nonsynony	TP53:NM_	NA	.	.
MALT_TH	TM_9	MALT_TH_chr4:10523	1	TET2	-204	-446	NA	-211.547	-37049%	-52943%	NA	exonic	stopgain	TET2:NM_	NA	.	rs751233118
MALT_TH	TM_9	MALT_TH_chr4:10524	1	TET2	-171	-387	NA	-203.52	-35645%	-50937%	NA	exonic	frameshift	TET2:NM_	NA	.	.
MALT_TH	TM_9	MALT_TH_chr9:5463(	1	CD275	-702	-2041	NA	-1742.76	-305013%	-435751%	NA	exonic	nonsynony	CD274:NM	NA	.	.
MALT_TH	TM_10	MALT_TH_chr1:25599	1	TNFRSF15	-20	-654	NA	-1244.01	-217734%	-311068%	NA	exonic	nonsynony	TNFRSF14:	NA	.	.
MALT_TH	TM_10	MALT_TH_chr22:4113	1	EP301	-203	-441	NA	-206.223	-36117%	-51611%	NA	splicing	.	.	NA	.	.
MALT_TH	TM_10	MALT_TH_chr14:1002	1	YY2	-621	-1444	NA	-820.837	-143676%	-205267%	NA	exonic	stopgain	YY1:NM_0	NA	.	.
MALT_TH	TM_10	MALT_TH_chr11:1188	1	CXCR6	-90	-210	NA	-119.5	-20942%	-29933%	NA	splicing	.	.	NA	.	.
MALT_TH	TM_10	MALT_TH_chr3:17705	1	TBL1XR2	-206	-537	NA	-388.12	-67951%	-97091%	NA	exonic	nonsynony	TBL1XR1:N	NA	.	.
MALT_TH	TM_10	MALT_TH_chr4:10526	1	TET2	-415	-959	NA	-534.172	-93509%	-133601%	NA	exonic	nonsynony	TET2:NM_	NA	.	.
MALT_TH	TM_10	MALT_TH_chr4:10527	1	TET2	-24	-105	NA	-130.044	-22790%	-32575%	NA	exonic	stopgain	TET2:NM_	NA	.	.
MALT_TH	TM_10	MALT_TH_chr9:54655	1	CD275	-438	-976	NA	-493.533	-86397%	-123440%	NA	exonic	nonsynony	CD274:NM	NA	.	.
MALT_TH	TM_26	MALT_TH_chr1:1115(	1	ADORA4	-223	-686	NA	-628.746	-110062%	-157249%	NA	exonic	nonsynony	ADORA3:N	NA	.	.
MALT_TH	TM_11	MALT_TH_chr3:18349	1	KLHL7	-332	-838	NA	-570.131	-99803%	-142593%	NA	exonic	nonsynony	KLHL6:NM	NA	.	.
MALT_TH	TM_11	MALT_TH_chr2:24063	1	GPR36	52	25	NA	-123.244	-21603%	-30882%	NA	exonic	nonsynony	GPR35:NM	NA	.	.
MALT_TH	TM_11	MALT_TH_chr4:10523	1	TET2	-523	-1066	NA	-388.299	-67978%	-97126%	NA	exonic	frameshift	TET2:NM_	NA	.	.
MALT_TH	TM_11	MALT_TH_chr4:10527	1	TET2	-350	-1379	NA	-1590.72	-278407%	-397743%	NA	exonic	frameshift	TET2:NM_	NA	.	.
MALT_TH	TM_12	MALT_TH_chr1:25578	1	TNFRSF15	999	1296	NA	-741.705	-129839%	-185508%	NA	exonic	stopgain	TNFRSF14:	NA	.	.
MALT_TH	TM_12	MALT_TH_chr11:1188	1	CXCR6	-584	-1392	NA	-840.157	-147057%	-210098%	NA	exonic	stopgain	CXCR5:NM	NA	.	.
MALT_TH	TM_12	MALT_TH_chr19:1439	1	ADGRE6	92	77	NA	-152.536	-26730%	-38207%	NA	exonic	nonsynony	ADGRE5:N	NA	.	.
MALT_TH	TM_12	MALT_TH_chr2:11669	1	NTSR3	-188	-721	NA	-815.385	-142724%	-203910%	NA	exonic	nonsynony	NTSR2:NM	NA	.	.
MALT_TH	TM_12	MALT_TH_chr16:3792	1	CREBBP	-35	-116	NA	-115.404	-20227%	-28914%	NA	exonic	stopgain	CREBBP:NI	NA	.	.
MALT_TH	TM_12	MALT_TH_chr16:3850	1	CREBBP	801	922	NA	-831.124	-145485%	-207858%	NA	exonic	nonsynony	CREBBP:NI	NA	.	rs756802947
MALT_TH	TM_12	MALT_TH_chr19:1632	3	KLF3	-216	-712	NA	-705.403	-123477%	-176414%	NA	exonic	nonsynony	KLF2:NM_(	NA	.	.
MALT_TH	TM_12	MALT_TH_chr4:10527	1	TET3	-142	-394	NA	-314.768	-55115%	-78754%	NA	exonic	stopgain	TET2:NM_	NA	.	.
MALT_TH	TM_12	MALT_TH_chr9:54573	1	CD275	1105	1387	NA	-909.064	-159126%	-227346%	NA	exonic	nonsynony	CD274:NM	NA	.	.
MALT_TH	TM_12	MALT_TH_chrX:7161(	1	CXCR4	-325	-856	NA	-628.782	-110067%	-157256%	NA	exonic	stopgain	CXCR3:NM	NA	.	.
MALT_TH	TM_13	MALT_TH_chr1:26774	1	ARID1A	-334	-889	NA	-664.112	-116250%	-166089%	NA	exonic	stopgain	ARID1A:NM	NA	.	.
MALT_TH	TM_49	MALT_TH_chr1:1115(	1	ADORA4	53	24	NA	-127.908	-22420%	-32048%	NA	exonic	nonsynony	ADORA3:N	NA	.	rs764124231
MALT_TH	TM_13	MALT_TH_chr6:11138	1	REV3L	124	158	NA	-96.3836	-16908%	-24177%	NA	exonic	nonsynony	REV3L:NM	NA	.	.
MALT_TH	TM_13	MALT_TH_chr3:17703	1	TBL1XR2	-172	-430	NA	-286.801	-50220%	-71760%	NA	exonic	nonsynony	TBL1XR1:N	NA	.	.
MALT_TH	TM_13	MALT_TH_chr4:10523	1	TET3	-61	-460	NA	-716.019	-125336%	-179070%	NA	exonic	frameshift	TET2:NM_	NA	.	.
MALT_TH	TM_13	MALT_TH_chr6:13787	1	TNFAIP4	-371	-1051	NA	-932.804	-163269%	-233258%	NA	exonic	frameshift	TNFAIP3:N	NA	.	.
MALT_TH	TM_13	MALT_TH_chr9:54561	1	CD275	18	20	NA	-19.1333	-3387%	-4860%	NA	exonic	nonsynony	CD274:NM	NA	.	rs111401208
MALT_TH	TM_14	MALT_TH_chr1:25577	1	TNFRSF15	-44	-170	NA	-192.717	-33758%	-48243%	NA	exonic	frameshift	TNFRSF14:	NA	.	.
MALT_TH	TM_14	MALT_TH_chr14:105(	1	GPR133	-469	-1037	NA	-510.871	-89431%	-127774%	NA	exonic	nonsynony	GPR132:NI	NA	.	rs201002261
MALT_TH	TM_14	MALT_TH_chr19:1632	1	KLF2	-101	-227	NA	-116.858	-20479%	-29272%	NA	exonic	nonsynony	KLF2:NM_(	NA	.	.
MALT_TH	TM_14	MALT_TH_chr19:1632	3	KLF2	-291	-668	NA	-365.51	-63993%	-91435%	NA	exonic	nonsynony	KLF2:NM_(	NA	.	.
MALT_TH	TM_14	MALT_TH_chr2:1361	1	CXCR5	-660	-1425	NA	-650.226	-113817%	-162612%	NA	exonic	nonsynony	CXCR4:NM	NA	.	.
MALT_TH	TM_14	MALT_TH_chr4:10524	1	TET2	-154	-405	NA	-296.783	-51967%	-74257%	NA	exonic	nonsynony	TET2:NM_	NA	.	.
MALT_TH	TM_14	MALT_TH_chr4:10527	1	TET2	-982	-2642	NA	-2010.78	-351916%	-502755%	NA	exonic	stopgain	TET2:NM_	NA	.	.
MALT_TH	TM_14	MALT_TH_chr9:54656	1	CD275	-191	-754	NA	-872.05	-152641%	-218077%	NA	splicing	.	.	NA	.	.
MALT_TH	TM_14	MALT_TH_chrX:7161	1	CXCR4	-718	-1849	NA	-1304.12	-228252%	-326091%	NA	exonic	nonsynony	CXCR3:NM	NA	.	.
MALT_TH	TM_15	MALT_TH_chr12:4902	1	KMT2D	-86	-335	NA	-382.717	-67007%	-95743%	NA	exonic	frameshift	KMT2D:NM	NA	.	.
MALT_TH	TM_11	MALT_TH_chr11:1082	1	ATM	-80	-369	NA	-475.377	-83223%	-118908%	NA	exonic	nonsynony	ATM:NM_(	NA	.	.
MALT_TH	TM_15	MALT_TH_chr4:10523	1	TET2	20	4	NA	-57.9242	-10172%	-14552%	NA	exonic	frameshift	TET2:NM_	NA	.	.
MALT_TH	TM_15	MALT_TH_chr4:10527	1	TET2	176	-555	NA	-1703.31	-298114%	-425896%	NA	exonic	stopgain	TET2:NM_	NA	.	.
MALT_TH	TM_16	MALT_TH_chr5:1510	1	TNIP2	-1563	-3510	NA	-1813.53	-317396%	-453439%	NA	exonic	nonsynony	TNIP1:NM_	NA	.	.
MALT_TH	TM_17	MALT_TH_chr4:10523	1	TET2	-249	-521	NA	-211.594	-37056%	-52952%	NA	exonic	frameshift	TET2:NM_	NA	.	.
MALT_TH	TM_17	MALT_TH_chr4:10523	1	TET2	-408	-878	NA	-395.562	-69251%	-98946%	NA	exonic	frameshift	TET2:NM_	NA	.	rs751193038
MALT_TH	TM_18	MALT_TH_chr1:25617	1	TNFRSF15	-446	-1324	NA	-1164.75	-203863%	-291251%	NA	exonic	nonsynony	TNFRSF14:	NA	.	.
MALT_TH	TM_18	MALT_TH_chr19:1632	3	KLF3	-379	-870	NA	-476.844	-83477%	-119269%	NA	exonic	nonsynony	KLF2:NM_(	NA	.	.
MALT_TH	TM_18	MALT_TH_chr4:10525	1	TET3	-220	-561	NA	-390.796	-68419%	-97759%	NA	exonic	nonsynony	TET2:NM_	NA	.	.
MALT_TH	TM_47	MALT_TH_chr11:1083	1	ATM	82	-154	NA	-583.973	-102229%	-146061%	NA	exonic	nonsynony	ATM:NM_(	NA	.	.
MALT_TH	TM_18	MALT_TH_chr9:54571	3	CD275	3	-9	NA	-27.3095	-4813%	-6895%	NA	exonic	stopgain	CD274:NM	NA	.	.
MALT_TH	TM_19	MALT_TH_chr1:25599	1	TNFRSF15	-891	-2595	NA	-2219.42	-388430%	-554918%	NA	exonic	frameshift	TNFRSF14:	NA	.	.
MALT_TH	TM_19	MALT_TH_chr1:2676(	1	ARID1A	-2222	-4551	NA	-1694.96	-296644%	-423792%	NA	exonic	frameshift	ARID1A:NM	NA	.	.
MALT_TH	TM_19	MALT_TH_chr13:3233	1	BRCA3	67	-113	NA	-449.301	-78662%	-112394%	NA	exonic	nonsynony	BRCA2:NM	NA	.	.
MALT_TH	TM_13	MALT_TH_chr4:1017	1	BANK2	-166	-668	NA	-783.382	-137124%	-195910%	NA	exonic	nonsynony	BANK1:NM	NA	.	.
MALT_TH	TM_19	MALT_TH_chr4:10523	1	TET3	-401	-885	NA	-432.872	-75781%	-108274%	NA	exonic	frameshift	TET2:NM_	NA	.	.
MALT_TH	TM_19	MALT_TH_chr9:5462	1	CD275	-403	-1052	NA	-760.785	-133168%	-190257%	NA	exonic	nonsynony	CD274:NM	NA	.	.
MALT_TH	TM_20	MALT_TH_chr1:2556(	1	TNFRSF15	-57	-178	NA	-165.41	-28978%	-41415%	NA	exonic	nonsynony	TNFRSF14:	NA	.	.
MALT_TH	TM_20	MALT_TH_chr4:10523	1	TET2	-460	-1221	NA	-908.112	-158950%	-227089%	NA	exonic	frameshift	TET2:NM_	NA	.	.
MALT_TH	TM_20	MALT_TH_chr4:10525	1	TET2	-267	-585	NA	-280.881	-49182%	-70276%	NA	exonic	nonsynony	TET2:NM_	NA	.	.
MALT_TH	TM_21	MALT_TH_chr4:10523	1	TET2	-91	-207	NA	-110.183	-19311%	-27603%	NA	exonic	stopgain	TET2:NM_	NA	.	rs780043982
MALT_TH	TM_21	MALT_TH_chr4:10524	1	TET2	503	709	NA	-257.568	-45119%	-64480%	NA	exonic	nonsynony	TET2:NM_	NA	.	rs367866583
MALT_TH	TM_21	MALT_TH_chr9:5462	1	CD275	-389	-903	NA	-510.171	-89309%	-127601%	NA	exonic	stopgain	CD274:NM	NA	.	.
MALT_TH	TM_22	MALT_TH_chr1:2669(	2	ARID1A	-58	-157	NA	-120.107	-21049%	-30088%	NA	exonic	stopgain	ARID1A:NM	NA	.	.
MALT_TH	TM_22	MALT_TH_chr22:410	2	EP301	-408	-1052	NA	-744.79	-130368%	-186258%	NA	exonic	nonsynony	EP300:NM	NA	.	.
MALT_TH	TM_22	MALT_TH_chr19:1632	2	KLF3	-471	-1070	NA	-570.184	-99811%	-142603%	NA	splicing	.	.	NA	.	.
MALT_TH	TM_22	MALT_TH_chr4:10527	3	TET3	-134	-431	NA	-416.074	-72844%	-104081%	NA	exonic	stopgain	TET2:NM_	NA	.	.
MALT_TH	TM_22	MALT_TH_chr6:1671	1	CCR7	41	32	NA	-72.5485	-12732%	-18209%	NA	exonic	frameshift	CCR6:NM_	NA	.	.
MALT_TH	TM_22	MALT_TH_chr9:5456	3	CD275	102	144	NA	-50.8974	-8951%	-12813%	NA	exonic	nonsynony	CD274:NM	NA	.	.
MALT_TH	TM_23	MALT_TH_chr4:10527	1	TET3	-76	-172	NA	-90.1863	-15811%	-22604%	NA	exonic	frameshift	TET2:NM_	NA	.	.
MALT_TH	TM_24	MALT_TH_chr1:25617	1	TNFRSF15	-55	-470	NA	-758.018	-132686%	-189570%	NA	exonic	stopgain	TNFRSF14:	NA	.	.
MALT_TH	TM_24	MALT_TH_chr11:1188	1	CXCR6	-415	-1063	NA	-743.458	-130135%	-185925%	NA	exonic	nonsynony	CXCR5:NM	NA	.	.
MALT_TH	TM_24	MALT_TH_chr1:26697	1	ARID1A	-23	-157	NA	-236.688	-41453%	-59238%	NA	exonic	frameshift	ARID1A:NM	NA	.	.
MALT_TH	TM_24	MALT_TH_chr4:10523	1	TET3	1098	1245	NA	-1173.13	-205336%	-293359%	NA	exonic	stopgain	TET2:NM_	NA	.	rs771408534
MALT_TH	TM_24	MALT_TH_chr6:13787	1	TNFAIP4	-268	-619	NA	-344.173	-60259%	-86101%	NA	exonic	frameshift	TNFAIP3:N	NA	.	.
MALT_TH	TM_24	MALT_TH_chr9:54655	1	CD275	-36	-132	NA	-143.389	-25125%	-35911%	NA	exonic	frameshift	CD274:NM	NA	.	.
MALT_TH	TM_25	MALT_TH_chr1:25577	1	TNFRSF15	274	305	NA	-303.803	-53204%	-76027%	NA	exonic	stopgain	TNFRSF14:	NA	.	.
MALT_TH	TM_25	MALT_TH_chr11:1188	2	CXCR6	-788	-1652	NA	-676.925	-118489%	-169285%	NA	exonic	frameshift	CXCR5:NM	NA	.	.
MALT_TH	TM_25	MALT_TH_chr12:4904	1	KMT2D	-180	-507	NA	-414.098	-72498%	-103586%	NA	exonic	nonsynony	KMT2D:NM	NA	.	.
MALT_TH	TM_25	MALT_TH_chr4:10524	1	TET2	22	-22	NA	-117.296	-20561%	-29392%	NA	exonic	stopgain	TET2:NM_	NA	.	.
MALT_TH	TM_25	MALT_TH_chr4:10525	1	TET2	-103	-307	NA	-270.085	-47296%	-67584%	NA	exonic	frameshift	TET2:NM_	NA	.	.
MALT_TH	TM_15	MALT_TH_chr4:10203	1	BANK2	46	6	NA	-140.594	-24639%	-35219%	NA	exonic	nonsynony	BANK1:NM	NA	.	.
MALT_TH	TM_26	MALT_TH_chr1:25598	1	TNFRSF15	325	237	NA	-609.883	-106766%	-152543%	NA	exonic	nonsynony	TNFRSF14:	NA	.	.
MALT_TH	TM_26	MALT_TH_chr12:4905	1	KMT2D	-314	-851	NA	-654.773	-114616%	-163755%	NA	splicing	.	.	NA	.	.
MALT_TH	TM_26	MALT_TH_chr14:1028	1	TRAF4	-480	-982	NA	-363.626	-63661%	-90959%	NA	exonic	frameshift	TRAF3:NM	NA	.	.
MALT_TH	TM_26	MALT_TH_chr6:13787	1	TNFAIP4	68	-75	NA	-376.628	-65944%	-94226%	NA	exonic	stopgain	TNFAIP3:N	NA	.	.
MALT_TH	TM_26	MALT_TH_chr9:5456	1	CD275	100	132	NA	-68.3462	-12002%	-17169%	NA	exonic	stopgain	CD274:NM	NA	.	.

MALT_TH	TM_27	MALT_TH_14_997	chr1:2559922_G>A	1	TNFRSF14	1178	515	NA	1699	69%	30%	NA	exonic	nonsynony	TNFRSF14:	NA	.	.	.	.	.	.	.	.	0	D	1	D	0.997	D	.	.	1	N	2.19	M	-0.1	T	-0.649	T	0.396	T	0.861	2.763	15.2	MALT_TH	TM_27	MALT_TH_	chr1:2559	1	TNFRSF15	-148	-811	NA	-1132.03	-198138%	-283073%	NA	exonic	nonsynony	TNFRSF14:	NA	.	.
MALT_TH	TM_27	MALT_TH_14_997	chr16:3728737_G>A	1	CREBBP	699	67	NA	766	91%	9%	NA	exonic	nonsynony	CREBBP:NM	NA	.	.	ID=COSM9	.	.	.	.	.	0	D	1	D	0.998	D	0	D	1	D	2.14	M	-2.34	D	0.709	D	0.788	D	0.919	0.787	8.149	MALT_TH	TM_27	MALT_TH_	chr16:3728	1	CREBBP	-565	-1197	NA	-510.246	-89320%	-127616%	NA	exonic	nonsynony	CREBBP:NM	NA	.	.
MALT_TH	TM_27	MALT_TH_14_997	chrX:40055460_A>G	1	BCOR	1006	159	NA	1169	86%	14%	NA	exonic	nonsynony	BCOR:NM_	NA	.	.	.	.	.	.	.	.	0	D	1	D	1	D	.	.	1	D	3.76	H	-0.77	T	0.504	D	0.615	D	0.983	3.639	18.51	MALT_TH	TM_27	MALT_TH_	chrX:4005	1	BCOR	-688	-1535	NA	-778.865	-136330%	-194773%	NA	exonic	nonsynony	BCOR:NM_	NA	.	.
MALT_TH	TM_27	MALT_TH_14_997	chr22:41147905_C>T	1	EP300	180	20	NA	200	90%	10%	NA	exonic	stopgain	EP300:NM	NA	.	.	.	.	.	.	.	.	0.78	T	.	.	.	.	0.014	N	1	A	.	.	.	.	.	.	.	.	.	16.29	50	MALT_TH	TM_27	MALT_TH_	chr22:4114	1	EP301	-140	-300	NA	-132.9	-23285%	-33280%	NA	exonic	stopgain	EP300:NM	NA	.	.
MALT_TH	TM_27	MALT_TH_14_997	chr4:105241412_A>T	1	TET2	187	29	NA	216	87%	13%	NA	exonic	nonsynony	TET2:NM_	NA	.	.	.	.	.	.	.	.	0.02	D	1	D	0.999	D	.	.	1	D	2.015	M	1.01	T	-0.599	T	0.261	T	0.993	3.656	18.58	MALT_TH	TM_27	MALT_TH_	chr4:1052	1	TET2	-129	-287	NA	-143.532	-25147%	-35940%	NA	exonic	nonsynony	TET2:NM_	NA	.	.
MALT_TH	TM_27	MALT_TH_14_997	chr4:105275233_C>-	1	TET2	229	43	NA	272	84%	16%	NA	exonic	frameshift	TET2:NM_	NA	.	.	.	.	.	.	.	.	.	.	.	.	.	.	.	.	.	.	.	.	.	.	.	.	.	.	.	.	.	MALT_TH	TM_27	MALT_TH_	chr4:1052	1	TET2	-143	-329	NA	-180.842	-31676%	-45268%	NA	exonic	frameshift	TET2:NM_	NA	.	.
MALT_TH	TM_27	MALT_TH_14_997	chr6:137874887_G>A	1	TNFAIP3	858	121	NA	985	87%	12%	NA	exonic	stopgain	TNFAIP3:N	NA	.	.	.	.	.	.	.	.	1	T	.	.	.	.	0	D	1	A	.	.	.	.	.	.	.	.	.	5.244	33	MALT_TH	TM_27	MALT_TH_	chr6:1378	1	TNFAIP3	-616	-1353	NA	-656.213	-114865%	-164109%	NA	exonic	stopgain	TNFAIP3:N	NA	.	.
MALT_TH	TM_27	MALT_TH_14_997	chr6:137878788_G>A	1	TNFAIP3	2057	332	NA	2395	86%	14%	NA	exonic	stopgain	TNFAIP3:N	NA	.	.	.	.	.	.	.	.	0.59	T	.	.	.	.	0	D	1	A	.	.	.	.	.	.	.	.	.	3.615	18.4	MALT_TH	TM_27	MALT_TH_	chr6:1378	1	TNFAIP3	-1393	-3118	NA	-1596.2	-279363%	-399106%	NA	exonic	stopgain	TNFAIP3:N	NA	.	.
MALT_TH	TM_28	MALT_TH_15_1172	chr22:41172632_G>A	1	EP300	1217	113	NA	1334	91%	8%	NA	exonic	nonsynony	EP300:NM	NA	.	.	.	.	.	.	.	.	0.19	T	1	D	0.998	D	0.001	N	0.999	D	0.855	L	-4.01	D	0.908	D	0.896	D	0.666	4.829	27.4	MALT_TH	TM_28	MALT_TH_	chr22:4117	1	EP301	-991	-2095	NA	-888.916	-155587%	-222283%	NA	exonic	nonsynony	EP300:NM	NA	.	.
MALT_TH	TM_28	MALT_TH_15_1172	chr4:105275364_C>G	2	TET2	1094	103	NA	1197	91%	9%	NA	exonic	stopgain	TET2:NM_	NA	.	.	ID=COSM9	.	.	.	.	.	1	T	.	.	.	.	.	.	1	D	.	.	.	.	.	.	.	.	.	15.303	49	MALT_TH	TM_28	MALT_TH_	chr4:1052	2	TET3	-888	-1879	NA	-797.581	-139604%	-199449%	NA	exonic	stopgain	TET2:NM_	NA	.	.
MALT_TH	TM_28	MALT_TH_15_1172	chr9:5457078_G>C	1	CD274	679	81	NA	765	89%	11%	NA	splicing	.	.	NA	.	.	.	.	.	.	.	.	.	.	.	.	.	.	.	.	1	D	.	.	.	.	.	.	.	.	.	2.969	15.9	MALT_TH	TM_28	MALT_TH_	chr9:5457	1	CD275	-517	-1115	NA	-509.563	-89201%	-127446%	NA	splicing	.	.	NA	.	.
MALT_TH	TM_29	MALT_TH_15_1458	chr11:118894508_T>G	1	CXCR5	2175	204	NA	2382	91%	9%	NA	exonic	nonsynony	CXCR5:NM	NA	.	.	.	.	.	.	.	.	0	D	1	D	1	D	0	D	1	D	3.83	H	-3.2	D	1.065	D	0.921	D	0.923	4.179	21.6	MALT_TH	TM_29	MALT_TH_	chr11:1188	1	CXCR6	-1767	-3738	NA	-1587.58	-277854%	-396950%	NA	exonic	nonsynony	CXCR5:NM	NA	.	.
MALT_TH	TM_29	MALT_TH_15_1458	chr19:16325390_T>A	1	KLF2	249	28	NA	277	90%	10%	NA	exonic	nonsynony	KLF2:NM_	NA	.	.	.	.	.	.	.	.	0.31	T	0.997	D	0.915	D	.	.	1	D	1.445	L	2.97	T	-1.055	T	0.041	T	0.218	3.103	16.37	MALT_TH	TM_29	MALT_TH_	chr19:1632	1	KLF2	-193	-414	NA	-184.232	-32268%	-46113%	NA	exonic	nonsynony	KLF2:NM_	NA	.	.
MALT_TH	TM_29	MALT_TH_15_1458	chr19:16325925_G>A	1	KLF2	147	29	NA	179	82%	16%	NA	exonic	nonsynony	KLF2:NM_	NA	.	.	.	.	.	.	.	.	0.02	D	1	D	0.981	D	.	.	1	D	2.33	M	2.75	T	-1.041	T	0.08	T	0.492	3.587	18.27	MALT_TH	TM_29	MALT_TH_	chr19:1632	1	KLF2	-89	-207	NA	-118.844	-20826%	-29768%	NA	exonic	nonsynony	KLF2:NM_	NA	.	.
MALT_TH	TM_29	MALT_TH_15_1458	chr19:16325964_G>C	2	KLF2	345	38	NA	384	90%	10%	NA	exonic	nonsynony	KLF2:NM_	NA	.	.	ID=COSM3	.	.	.	.	.	0.47	T	0.04	B	0.056	B	.	.	1	D	0.735	N	3.17	T	-0.973	T	0.015	T	0.125	-1.156	0.098	MALT_TH	TM_29	MALT_TH_	chr19:1632	2	KLF2	-269	-576	NA	-255.569	-44752%	-63947%	NA	exonic	nonsynony	KLF2:NM_	NA	.	.
MALT_TH	TM_29	MALT_TH_15_1458	chr3:45901543_C>T	1	CCR9	1043	80	NA	1123	93%	7%	NA	exonic	nonsynony	CCR9:NM_	NA	.	.	.	.	.	.	.	.	0	D	0.999	D	0.995	D	0.001	D	1	D	1.875	L	1.18	T	-0.78	T	0.187	T	0.809	4.44	23.6	MALT_TH	TM_29	MALT_TH_	chr3:4590	1	CCR10	-883	-1846	NA	-748.262	-130973%	-187119%	NA	exonic	nonsynony	CCR9:NM_	NA	.	.
MALT_TH	TM_29	MALT_TH_15_1458	chr4:105259667_CTT>-	1	TET2	417	145	NA	562	74%	26%	NA	exonic	nonframes	TET2:NM_	NA	.	.	ID=COSM2	.	.	.	.	.	.	.	.	.	.	.	.	.	.	.	.	.	.	.	.	.	.	.	.	.	.	MALT_TH	TM_29	MALT_TH_	chr4:1052	1	TET3	-127	-399	NA	-374.075	-65495%	-93582%	NA	exonic	nonframes	TET2:NM_	NA	.	.
MALT_TH	TM_30	MALT_TH_15_1575	chr1:2557750_GCCCCCT>-	1	TNFRSF14	629	259	NA	888	71%	29%	NA	exonic	frameshift	TNFRSF14:	NA	.	.	.	.	.	.	.	.	.	.	.	.	.	.	.	.	.	.	.	.	.	.	.	.	.	.	.	.	.	MALT_TH	TM_30	MALT_TH_	chr1:2557	1	TNFRSF15	-111	-481	NA	-591.375	-103523%	-147908%	NA	exonic	frameshift	TNFRSF14:	NA	.	.
MALT_TH	TM_30	MALT_TH_15_1575	chr4:105237289_->A	1	TET2	212	58	NA	270	79%	21%	NA	exonic	frameshift	TET2:NM_	NA	.	rs7484193	.	.	.	.	.	.	.	.	.	.	.	.	.	.	.	.	.	.	.	.	.	.	.	.	.	.	.	MALT_TH	TM_30	MALT_TH_	chr4:1052	1	TET3	-96	-250	NA	-179.452	-31434%	-44924%	NA	exonic	frameshift	TET2:NM_	NA	.	rs748419341
MALT_TH	TM_30	MALT_TH_15_1575	chr6:137877183_G>T	1	TNFAIP3	1342	135	NA	1479	91%	9%	NA	exonic	stopgain	TNFAIP3:N	NA	.	.	.	.	.	.	.	.	0.33	T	.	.	.	.	0	D	1	A	.	.	.	.	.	.	.	.	.	7.065	38	MALT_TH	TM_30	MALT_TH_	chr6:1378	1	TNFAIP4	-1072	-2279	NA	-985.576	-172503%	-246448%	NA	exonic	stopgain	TNFAIP3:N	NA	.	.
MALT_TH	TM_31	MALT_TH_15_1581	chr4:105236166_C>T	1	TET2	95	20	NA	116	82%	17%	NA	exonic	stopgain	TET2:NM_	NA	.	.	ID=COSM4	.	.	.	.	.	0.07	T	.	.	.	.	0.03	U	1	D	.	.	.	.	.	.	.	.	.	8.181	40	MALT_TH	TM_31	MALT_TH_	chr4:1052	1	TET3	-55	-130	NA	-76.8305	-13474%	-19266%	NA	exonic	stopgain	TET2:NM_	NA	.	.
MALT_TH	TM_31	MALT_TH_15_1581	chr3:39332333_T>C	1	CCR8	192	185	NA	379	51%	49%	NA	exonic	nonsynony	CCR8:NM_	NA	.	.	.	.	.	.	.	.	0	D	0.995	D	0.795	P	0.019	N	1	D	.	.	2.06	T	-1.049	T	0.092	T	0.851	1.456	10.81	MALT_TH	TM_31	MALT_TH_	chr3:3933	1	CCR9	178	171	NA	-251.847	-44110%	-63036%	NA	exonic	nonsynony	CCR8:NM_	NA	.	.
MALT_TH	TM_31	MALT_TH_15_1581	chr4:105275364_C>G	2	TET2	867	244	NA	1113	78%	22%	NA	exonic	stopgain	TET2:NM_	NA	.	.	ID=COSM9	.	.	.	.	.	1	T	.	.	.	.	.	.	1	D	.	.	.	.	.	.	.	.	.	15.303	49	MALT_TH	TM_31	MALT_TH_	chr4:1052	2	TET3	-379	-1002	NA	-741.448	-129784%	-185423%	NA	exonic	stopgain	TET2:NM_	NA	.	.
MALT_TH	TM_31	MALT_TH_15_1581	chr6:137875994_A>T	1	TNFAIP3	1324	395	NA	1723	77%	23%	NA	splicing	.	.	NA	.	.	.	.	.	.	.	.	.	.	.	.	.	.	.	.	1	D	.	.	.	.	.	.	.	.	.	4.245	22.1	MALT_TH	TM_31	MALT_TH_	chr6:1378	1	TNFAIP4	-534	-1463	NA	-1148.1	-200949%	-287088%	NA	splicing	.	.	NA	.	.
MALT_TH	TM_31	MALT_TH_15_1581	chr9:5457111_G>-	1	CD274	551	176	NA	727	76%	24%	NA	exonic	frameshift	CD274:NM	NA	.	.	.	.	.	.	.	.	.	.	.	.	.	.	.	.	.	.	.	.	.	.	.	.	.	.	.	.	.	MALT_TH	TM_31	MALT_TH_	chr9:5457	1	CD275	-199	-574	NA	-484.091	-84747%	-121085%	NA	exonic	frameshift	CD274:NM	NA	.	.
MALT_TH	TM_32	MALT_TH_15_1713	chr1:2557819_C>T	1	TNFRSF14	453	296	NA	751	60%	39%	NA	exonic	nonsynony	TNFRSF14:	NA	.	.	.	.	.	.	.	.	0.29	T	1	D	0.999	D	.	.	0.818	D	1.645	L	-0.38	T	-0.386	T	0.352	T	0.384	2.027	12.73	MALT_TH	TM_32	MALT_TH_	chr1:2557	1	TNFRSF15	139	-18	NA	-499.94	-87524%	-125055%	NA	exonic	nonsynony	TNFRSF14:	NA	.	.
MALT_TH	TM_32	MALT_TH_15_1713	chr19:16326917_GCTCACGCGCC>	1	KLF2	439	103	NA	542	81%	19%	NA	exonic	frameshift	KLF2:NM_	NA	.	.	.	.	.	.	.	.	.	.	.	.	.	.	.	.	.	.	.	.	.	.	.	.	.	.	.	.	.	MALT_TH	TM_32	MALT_TH_	chr19:1632	1	KLF3	-233	-569	NA	-360.81	-63171%	-90262%	NA	exonic	frameshift	KLF2:NM_	NA	.	.
MALT_TH	TM_32	MALT_TH_15_1713	chr4:105236638_A>-	1	TET2	1118	289	NA	1407	79%	21%	NA	exonic	frameshift	TET2:NM_	NA	.	.	.	.	.	.	.	.	.	.	.	.	.	.	.	.	.	.	.	.	.	.	.	.	.	.	.	.	.	MALT_TH	TM_32	MALT_TH_	chr4:1052	1	TET2	-540	-1369	NA	-937.461	-164086%	-234426%	NA	exonic	frameshift	TET2:NM_	NA	.	.
MALT_TH	TM_32	MALT_TH_15_1713	chr4:105259628_C>G	1	TET2	431	93	NA	526	82%	18%	NA	exonic	nonsynony	TET2:NM_	NA	.	.	ID=COSM1	.	.	.	.	.	0	D	1	D	1	D	.	.	1	D	2.865	M	1.99	T	-0.926	T	0.145	T	0.962	1.904	12.33	MALT_TH	TM_32	MALT_TH_	chr4:1052	1	TET2	-245	-583	NA	-350.158	-61307%	-87598%	NA	exonic	nonsynony	TET2:NM_	NA	.	.
MALT_TH	TM_33	MALT_TH_15_1892	chr1:2557781_G>A	1	TNFRSF14	392	281	NA	679	58%	41%	NA	exonic	nonsynony	TNFRSF14:	NA	.	.	ID=COSM1	.	.	.	.	.	0	D	0.999	D	0.995	D	.	.	0.967	D	2.645	M	-1.26	T	0.258	D	0.595	D	0.56	1.815	12.03	MALT_TH	TM_33	MALT_TH_	chr1:2557	1	TNFRSF15	170	59	NA	-451.922	-79122%	-113051%	NA	exonic	nonsynony	TNFRSF14:	NA	.	.
MALT_TH	TM_33	MALT_TH_15_1892	chr4:105272735_C>T	3	TET2	651	277	NA	931	70%	30%	NA	exonic	stopgain	TET2:NM_	NA	.	.	ID=COSM4	.	.	.	.	.	1	T	.	.	.	.	.	.	1	D	.	.	.	.	.	.	.	.	.	14.765	48	MALT_TH	TM_33	MALT_TH_	chr4:1052	3	TET3	-97	-471	NA	-620.037	-108539%	-155074%	NA	exonic	stopgain	TET2:NM_	NA	.	.
MALT_TH	TM_33	MALT_TH_15_1892	chr6:137877191_AATGG>-	1	TNFAIP3	1266	212	NA	1478	86%	14%	NA	exonic	frameshift	TNFAIP3:N	NA	.	.	.	.	.	.	.	.	.	.	.	.	.	.	.	.	.	.	.	.	.	.	.	.	.	.	.	.	.	MALT_TH	TM_33	MALT_TH_	chr6:1378	1	TNFAIP3	-842	-1896	NA	-984.857	-172378%	-246271%	NA	exonic	frameshift	TNFAIP3:N	NA	.	.
MALT_TH	TM_33	MALT_TH_15_1892	chr6:137877257_G>A	1	TNFAIP3	1371	605	NA	1978	69%	31%	NA	splicing	.	.	NA	.	.	.	.	.	.	.	.	.	.	.	.	.	.	.	.	1	D	.	.	.	.	.	.	.	.	.	4.612	25.2	MALT_TH	TM_33	MALT_TH_	chr6:1378	1	TNFAIP3	-161	-927	NA	-1318.03	-230687%	-329572%	NA	splicing	.	.	NA	.	.
MALT_TH	TM_33	MALT_TH_15_1892	chr9:5465559_T>A	1	CD274	356	74	NA	430	83%	17%	NA	exonic	stopgain	CD274:NM	NA	.	.	.	.	.	.	.	.	1	T	.	.	.	.	0.823	N	1	A	.	.	.	.	.	.	.	.	.	2.831	15.43	MALT_TH	TM_33	MALT_TH_	chr9:5465	1	CD275	-208	-490	NA	-286.161	-50108%	-71599%	NA	exonic	stopgain	CD274:NM	NA	.	.
MALT_TH	TM_33	MALT_TH_15_1892	chrX:71616402_C>T	1	CXCR3	848	133	NA	984	86%	14%	NA	exonic	stopgain	CXCR3:NM	NA	.	.	.	.	.	.	.	.	0.03	D	.	.	.	.	.	.	1	D	.	.	.	.	-0.291	T	0.372	T	.	5.638	36	MALT_TH	TM_33	MALT_TH_	chrX:7161	1	CXCR4	-582	-1297	NA	-655.533	-114746%	-163940%	NA	exonic	stopgain	CXCR3:NM	NA	.	.
MALT_TH	TM_34	MALT_TH_15_388_1	chr16:3731449_C>T	1	CREBBP	984	738	NA	1731	57%	43%	NA	exonic	nonsynony	CREBBP:NM	NA	.	rs7701549	.	.	.	.	.	.	0.15	T	0.999	D	0.949	D	0	D	1	D	1.69	L	-3.22	D	0.778	D	0.861	D	0.682	3.326	17.19	MALT_TH	TM_34	MALT_TH_	chr16:3731	1	CREBBP	492	246	NA	-1153.24	-201853%	-288382%	NA	exonic	nonsynony	CREBBP:NM	NA	.	rs770154927
MALT_TH	TM_34	MALT_TH_15_388_1	chr2:230910540_G>C	1	GPR55	694	598	NA	1294	54%	46%	NA	exonic	nonsynony	GPR55:NM	NA	.	.	.	.	.	.	.	.	0.02	D	0.973	D	0.9	P	0.067	N	0.866	D	2.405	M	1.16	T	-0.721	T	0.167	T	0.154	3.025	16.09	MALT_TH	TM_34	MALT_TH_	chr2:2309	1	GPR56	502	406	NA	-861.872	-150864%	-215541%	NA	exonic	nonsynony	GPR55:NM	NA	.	.
MALT_TH	TM_35	MALT_TH_15_593	chr1:2560657_->C	1	TNFRSF14	1036	98	NA	1134	91%	9%	NA	exonic	frameshift	TNFRSF14:	NA	.	rs7720884	.	.	.	.	.	.	.	.	.	.	.	.	.	.	.	.	.	.	.	.	.	.	.	.	.	.	.	MALT_TH	TM_35	MALT_TH_	chr1:2560	1	TNFRSF15	-840	-1778	NA	-755.58	-132254%	-188949%	NA	exonic	frameshift	TNFRSF14:	NA	.	rs772088411
MALT_TH	TM_35	MALT_TH_15_593	chr12:49041953_C>T	1	KMT2D	746	78	NA	828	90%	9%	NA	exonic	stopgain	KMT2D:NM	NA	.	.	.	.	.	.	.	.	0	D	.	.	.	.	0.003	N	1	A	.	.	.	.	.	.	.	.	.	12.758	46	MALT_TH	TM_35	MALT_TH_	chr12:4904	1	KMT2D	-590	-1258	NA	-551.574	-96553%	-137948%	NA	exonic	stopgain	KMT2D:NM	NA	.	.
MALT_TH	TM_35	MALT_TH_15_593	chr6:111373179_C>A	1	REV3L	630	80	NA	711	89%	11%	NA	exonic	stopgain	REV3L:NM	NA	.	.	.	.	.	.	.	.	0.09	T	.	.	.	.	0	D	1	A	.	.	.	.	.	.	.	.	.	15.98	49	MALT_TH	TM_35	MALT_TH_	chr6:1113	1	REV3L	-470	-1020	NA	-473.555	-82900%	-118444%	NA	exonic	stopgain	REV3L:NM	NA	.	.
MALT_TH	TM_35	MALT_TH_15_593	chr4:105275922_->AA	1	TET2	737	81	NA	818	90%	10%	NA	exonic	frameshift	TET2:NM_	NA	.	.	.	.	.	.	.	.	.	.	.	.	.	.	.	.	.	.	.	.	.	.	.	.	.	.	.	.	.	MALT_TH	TM_35	MALT_TH_	chr4:1052	1	TET2	-575	-1231	NA	-544.901	-95385%	-136280%	NA	exonic	frameshift	TET2:NM_	NA	.	.
MALT_TH	TM_35	MALT_TH_15_593	chr4:105276217_CAGCAT>-	1	TET2	1042	104	NA	1146	91%	9%	NA	exonic	nonframes	TET2:NM_	NA	.	.	.	.	.	.	.	.	.	.	.	.	.	.	.	.	.	.	.	.	.	.	.	.	.	.	.	.	.	MALT_TH	TM_35	MALT_TH_	chr4:1052	1	TET2	-834	-1772	NA	-763.576	-133653%	-190949%	NA	exonic	nonframes	TET2:NM_	NA	.	.
MALT_TH	TM_35	MALT_TH_15_593	chr6:137874985_TC>-	2	TNFAIP3	2143	72	NA	2215	97%	3%	NA	exonic	frameshift	TNFAIP3:N	NA	.	.	ID=COSM3	.	.	.	.	.	.	.	.	.	.	.	.	.	.	.	.	.	.	.	.	.	.	.	.	.	.	MALT_TH	TM_35	MALT_TH_	chr6:1378	2	TNFAIP4	-1999	-4070	NA	-1476.3	-258378%	-369127%	NA	exonic	frameshift	TNFAIP3:N	NA	.	.
MALT_TH	TM_36	MALT_TH_15_603	chr3:177050481_G>A	1	TBL1XR1	605	57	NA	666	91%	9%	NA	exonic	nonsynony	TBL1XR1:N	NA	.	.	.	.	.	.	.	.	0	D	0.999	D	0.99	D	0	D	1	D	3.625	H	-0.67	T	0.565	D	0.667	D	0.944	5.478	35	MALT_TH	TM_36	MALT_TH_	chr3:1770	1	TBL1XR2	-491	-1039	NA	-443.583	-77654%	-110950%	NA	exonic	nonsynony	TBL1XR1:N	NA	.	.
MALT_TH	TM_36	MALT_TH_15_603	chr4:105236609_GT>-	1	TET2	89	27	NA	116	77%	23%	NA	exonic	frameshift	TET2:NM_	NA	.	.	.	.	.	.	.	.	.	.	.	.	.	.	.	.	.	.	.	.	.	.	.	.	.	.	.	.	.	MALT_TH	TM_36	MALT_TH_	chr4:1052	1	TET3	-35	-97	NA	-76.7672	-13465%	-19253%	NA	exonic	frameshift	TET2:NM_	NA	.	.
MALT_TH	TM_36	MALT_TH_15_603	chr3:151294497_T>A	1	GPR87	428	443	NA	871	49%	51%	NA	exonic	nonsynony	GPR87:NM	NA	.	.	.	.	.	.	.	.	0.09	T	0.985	D	0.76	P	0	D	1	D	0.405	N	1.43	T	-1.054	T	0.085	T	0.796	1.692	11.62	MALT_TH	TM_36	MALT_TH_	chr3:1512	1	GPR88	458	473	NA	-579.825	-101507%	-145032%	NA	exonic	nonsynony	GPR87:NM	NA	.	.
MALT_TH	TM_36	MALT_TH_15_603	chr4:105272621_C>T	1	TET2	2422	335	NA	2761	88%	12%	NA	exonic	stopgain	TET2:NM_	NA	.	.	.	.	.	.	.	.	0	D	.	.	.	.	.	.	1	D	.	.	.	.	.	.	.	.	.	16.24	50	MALT_TH	TM_36	MALT_TH_	chr4:1052	1	TET3	-1752	-3839	NA	-1840.21	-322065%	-460109%	NA	exonic	stopgain	TET2:NM_	NA	.	.
MALT_TH	TM_36	MALT_TH_15_603	chr6:137876153_G>-	1	TNFAIP3	2145	86	NA	2231	96%	4%	NA	exonic	frameshift	TNFAIP3:N	NA	.	.	.	.	.	.	.	.	.	.	.	.	.	.	.	.	.	.	.	.	.	.	.	.	.	.	.	.	.	MALT_TH	TM_36	MALT_TH_	chr6:1378	1	TNFAIP4	-1973	-4032	NA	-1486.96	-260244%	-371792%	NA	exonic	frameshift	TNFAIP3:N	NA	.	.
MALT_TH	TM_36	MALT_TH_15_603	chr9:5456116_G>T	3	CD274	61	12	NA	73	84%	16%	NA	exonic	nonsynony	CD274:NM	NA	.	.	.	.	.	.	.	.	0	D	0.993	D	0.971	D	0	D	0.937	N	.	.	5.21	T	-0.828	T	0.214	T	0.932	2.369	13.88	MALT_TH	TM_36	MALT_TH_	chr9:5456	3	CD275	-37	-86	NA	-48.1689	-8459%	-12100%	NA	exonic	nonsynony	CD274:NM	NA	.	.
MALT_TH	TM_37	MALT_TH_15_678	chr1:2557792_G>A	1	TNFRSF14	182	269	NA	454	40%	59%	NA	exonic	nonsynony	TNFRSF14:	NA	.	.	.	.	.	.	.	.	0.04	D	1	D	0.992	D	.	.	0.511	N	2.415	M	-0.56	T	-0.004	T	0.485	T	0.457	2.773	15.24	MALT_TH	TM_37	MALT_TH_	chr1:2557	1	TNFRSF15	356	443	NA	-301.743	-52845%	-75515%	NA	exonic	nonsynony	TNFRSF14:	NA	.	.
MALT_TH	TM_37	MALT_TH_15_678	chr1:202280812_G>A	1	LGR6	450	183	NA	634	71%	29%	NA	exonic	nonsynony	LGR6:NM_	NA	.	.	.	.	.	.	.	.	0.08	T	1	D	0.999	D	0	D	1	D	1.045	L	3.55	T	-1.106	T	0.033	T	0.762	4.793	27	MALT_TH	TM_37	MALT_TH_	chr1:2022	1	LGR6	-84	-351	NA	-422.045	-73890%	-105576%	NA	exonic	nonsynony	LGR6:NM_	NA	.	.
MALT_TH	TM_37	MALT_TH_15_678	chr1:202280813_G>A	1	LGR6	450	182	NA	633	71%	29%	NA	exonic	nonsynony	LGR6:NM_	NA	.	.	.	.	.	.	.	.	0.03	D	1	D	0.994	D	0	D	1	D	-0.215	N	3.58	T	-0.836	T	0.021	T	0.738	4.287	22.4	MALT_TH	TM_37	MALT_TH_	chr1:2022	1	LGR6	-86	-354	NA	-421.38	-73774%	-105409%	NA	exonic	nonsynony	LGR6:NM_	NA	.	.
MALT_TH	TM_37	MALT_TH_15_678	chr4:105235887_C>T	1	TET2	648	329	NA	978	66%	34%	NA	exonic	stopgain	TET2:NM_	NA	.	.	ID=COSM1	.	.	.	.	.	0.04	D	.	.	.	.	0.088	N	1	D	.	.	.	.	.	.	.	.	.	8.251	40	MALT_TH	TM_37	MALT_TH_	chr4:1052	1	TET3	10	-309	NA	-651.331	-114016%	-162899%	NA	exonic	stopgain	TET2:NM_	NA	.	.
MALT_TH	TM_37	MALT_TH_15_678	chr6:137871524_T>A	1	TNFAIP3	1139	484	NA	1626	70%	30%	NA	splicing	.	.	NA	.	.	.	.	.	.	.	.	.	.	.	.	.	.	.	.	1	D	.	.	.	.	.	.	.	.	.	3.152	16.54	MALT_TH	TM_37	MALT_TH_	chr6:1378	1	TNFAIP4	-171	-826	NA	-1083.37	-189622%	-270907%	NA	splicing	.	.	NA	.	.
MALT_TH	TM_37	MALT_TH_15_678	chrX:1466078_T>G	1	P2RY8	1039	408	NA	1448	72%	28%	NA	exonic	nonsynony	P2RY8:NM	NA	.	.	.	.	.	.	.	.	0.2	T	0.994	D	0.925	D	0.001	U	1	N	2.22	M	-0.65	T	-0.629	T	0.412	T	0.657	1.378	10.54	MALT_TH	TM_37	MALT_TH_	chrX:1466	1	P2RY9	-223	-854	NA	-964.718	-168858%	-241244%	NA	exonic	nonsynony	P2RY8:NM	NA	.	.
MALT_TH	TM_37	MALT_TH_15_678	chr6:137878982_->C	1	TNFAIP3	544	103	NA	647	84%	16%	NA	exonic	frameshift	TNFAIP3:N	NA	.	.	.	.	.	.	.	.	.	.	.	.	.	.	.	.	.	.	.	.	.	.	.	.	.	.	.	.	.	MALT_TH	TM_37	MALT_TH_	chr6:1378	1	TNFAIP4	-338	-779	NA	-430.841	-75426%	-107768%	NA	exonic	frameshift	TNFAIP3:N	NA	.	.
MALT_TH	TM_37	MALT_TH_15_678	chr9:5456167_T>G	1	CD274	33	30	NA	63	52%	48%	NA	splicing	.	.	NA	.	.	.	.	.	.	.	.	.	.	.	.	.	.	.	.	1	D	.	.	.	.	.	.	.	.	.	2.871	15.56	MALT_TH	TM_37	MALT_TH_	chr9:5456	1	CD275	27	24	NA	-41.1905	-7245%	-10371%	NA	splicing	.	.	NA	.	.
MALT_TH	TM_38	MALT_TH_15_832	chr1:2556666_T>C	3	TNFRSF14	361	458	NA	820	44%	56%	NA	exonic	nonsynony	TNFRSF14:	NA	.	rs7532686	.	.	.	.	.	.	0	D	0.967	D	0.879	P	.	.	1	D	.	.	-2.29	D	0.268	D	0.716	D	0.766	2.019	12.71	MALT_TH	TM_38	MALT_TH_	chr1:2556	3	TNFRSF15	555	652	NA	-545.775	-95550%	-136522%	NA	exonic	nonsynony	TNFRSF14:	NA	.	rs753268655
MALT_TH	TM_38	MALT_TH_15_832	chr3:183492091_C>T	2	KLHL6	2174	717	NA	2895	75%	25%	NA	exonic	nonsynony	KLHL6:NM	NA	.	.	.	.	.	.	.	.	0.08	T	0.976	D	0.821	P	0	D	0.999	D	1.53	L	-1.21	T	-0.011	T	0.513	D	0.522	4.091	21.1	MALT_TH	TM_38	MALT_TH_	chr3:1834	2	KLHL7	-740	-2197	NA	-1929.42	-337680%	-482417%	NA	exonic	nonsynony	KLHL6:NM	NA	.	.
MALT_TH	TM_38	MALT_TH_15_832	chr4:105236891_G>-	2	TET2	736	1075	NA	1810	41%	59%	NA	exonic	frameshift	TET2:NM_	NA	.	.	.	.	.	.	.	.	.	.	.	.	.	.	.	.	.	.	.	.	.	.	.	.	.	.	.	.	.	MALT_TH	TM_38	MALT_TH_	chr4:1052	2	TET2	1414	1753	NA	-1205.74	-211044%	-301515%	NA	exonic	frameshift	TET2:NM_	NA	.	.
MALT_TH	TM_38	MALT_TH_15_832	chr4:105236892_G>C	2	TET2	737	1070	NA	1810	41%	59%	NA	exonic	nonsynony	TET2:NM_	NA	.	.	.	.	.	.	.	.	0.08	T	1	D	1	D	0.033	U	1	D	1.04	L	2.26	T	-1.034	T	0.127	T	0.643	3.796	19.27	MALT_TH	TM_38	MALT_TH_	chr4:1052	2	TET2	1403	1736	NA	-1205.74	-211045%	-301515%	NA	exonic	nonsynony	TET2:NM_	NA	.	.
MALT_TH	TM_38	MALT_TH_15_832	chr6:137875765_C>G	2	TNFAIP3	1504	1945	NA	3454	44%	56%	NA	exonic	stopgain	TNFAIP3:N	NA	.	.	.	.	.	.	.	.	0.08	T	.	.	.	.	.	.	1	A	.	.	.	.	-1.012	T	0.022	T	.	5.044	29.9	MALT_TH	TM_38	MALT_TH_	chr6:1378	2	TNFAIP3	2386	2827	NA	-2301.77	-402849%	-575521%	NA	exonic	stopgain	TNFAIP3:N	NA	.	.
MALT_TH	TM_38	MALT_TH_15_832	chr6:137875782_T>C	2	TNFAIP3	1502	1946	NA	3452	44%	56%	NA	exonic	nonsynony	TNFAIP3:N	NA	.	.	ID=COSM3	.	.	.	.	.	0.01	D	0.987	D	0.988	D	0	D	1	D	1.91	M	1.33	T	-0.464	T	0.281	T	0.941	4.7	26	MALT_TH	TM_38	MALT_TH_	chr6:1378	2	TNFAIP3	2390	2834	NA	-2300.44	-402615%	-575187%	NA	exonic	nonsynony	TNFAIP3:N	NA	.	.
MALT_TH	TM_38	MALT_TH_15_832	chr9:5457141_G>T	3	CD274	36	14	NA	50	72%	28%	NA	exonic	stopgain	CD274:NM	NA	.	.	.	.	.	.	.	.	0.27	T	.	.	.	.	0	D	1	D	.	.	.	.	.	.	.	.	.	6.55	37	MALT_TH	TM_38	MALT_TH_	chr9:5457	3	CD275	-8	-30	NA	-32.72	-5758%	-8244%	NA	exonic	stopgain	CD274:NM	NA	.	.
MALT_TH	TM_39	MALT_TH_15_920	chr12:49032070_C>T	1	KMT2D	1323	611	NA	1942	68%	31%	NA	exonic	nonsynony	KMT2D:NM	NA	.	rs7726655	.	.	.	.	.	.	0	D	0.999	D	0.805	P	0.005	N	0.826	D	0	N	-1.49	T	-0.037	T	0.378	T	0.316	1.458	10.82	MALT_TH	TM_39	MALT_TH_	chr12:4903	1	KMT2D	-101	-813	NA	-1294.02	-226486%	-323571%	NA	exonic	nonsynony	KMT2D:NM	NA	.	rs772665566
MALT_TH	TM_39	MALT_TH_15_920	chr16:3782916_C>-	1	CREBBP	124	83	NA	207	60%	40%	NA	exonic	frameshift	CREBBP:NM	NA	.	.	.	.	.	.	.	.	.	.	.	.	.	.	.	.	.	.	.	.	.	.	.	.	.	.	.	.	.	MALT_TH	TM_39	MALT_TH_	chr16:3782	1	CREBBP	42	1	NA	-137.266	-24057%	-34386%	NA	exonic	frameshift	CREBBP:NM	NA	.	.
MALT_TH	TM_39	MALT_TH_15_920	chr5:151035584_G>A	1	TNIP1	1431	184	NA	1618	88%	11%	NA	exonic	stopgain	TNIP1:NM_	NA	.	.	.	.	.	.	.	.	0.04	D	.	.	.	.	0	D	1	A	.	.	.	.	.	.	.	.	.	6.84	38	MALT_TH	TM_39	MALT_TH_	chr5:1510	1	TNIP2	-1063	-2310	NA	-1078.22	-188716%	-269611%	NA	exonic	stopgain	TNIP1:NM_	NA	.	.
MALT_TH	TM_39	MALT_TH_15_920	chr14:100262332_AG>-	1	YY1	265	85	NA	350	76%	24%	NA	exonic	frameshift	YY1:NM_0	NA	.	.	.	.	.	.	.	.	.	.	.	.	.	.	.	.	.	.	.	.	.	.	.	.	.	.	.	.	.	MALT_TH	TM_39	MALT_TH_	chr14:1002	1	YY1	-95	-275	NA	-232.757	-40764%	-58251%	NA	exonic	frameshift	YY1:NM_0	NA	.	.
MALT_TH	TM_39	MALT_TH_15_920	chr14:100239461_CACCACCAT>-	1	YY1	1467	66	NA	1533	96%	4%	NA	exonic	nonframes	YY1:NM_0	NA	.	rs7673728	.	.	.	.	.	.	.	.	.	.	.	.	.	.	.	.	.	.	.	.	.	.	.	.	.	.	.	MALT_TH	TM_39	MALT_TH_	chr14:1002	1	YY1	-1335	-2736	NA	-1021.62	-178810%	-255458%	NA	exonic	nonframes	YY1:NM_0	NA	.	rs767372866
MALT_TH	TM_40	MALT_TH_16_1307	chr1:2559897_T>C	1	TNFRSF14	344	129	NA	474	73%	27%	NA	exonic	nonsynony	TNFRSF14:	NA	.	.	.	.	.	.	.	.	0	D	1	D	0.997	D	.	.	1	D	2.975	M	-0.03	T	-0.088	T	0.443	T	0.733	2.325	13.73	MALT_TH	TM_40	MALT_TH_	chr1:2559	1	TNFRSF15	-86	-301	NA	-315.395	-55226%	-78912%	NA	exonic	nonsynony	TNFRSF14:	NA	.	.
MALT_TH	TM_40	MALT_TH_16_1307	chr4:105242929_T>G	1	TET2	594	116	NA	711	84%	16%	NA	splicing	.	.	NA	.	.	.	.	.	.	.	.	.	.	.	.	.	.	.	.	1	D	.	.	.	.	.	.	.	.	.	4.137	21.4	MALT_TH	TM_40	MALT_TH_	chr4:1052	1	TET2	-362	-840	NA	-473.504	-82892%	-118434%	NA	splicing	.	.	NA	.	.
MALT_TH	TM_41	MALT_TH_16_1621	chr4:105269640_C>A	2	TET2	444	142	NA	586	76%	24%	NA	exonic	nonsynony	TET2:NM_	TRUE	.	rs7596580	.	.	.	.	.	.	0.07	T	1	D	0.99	D	.	.	1	D	2.725	M	2.63	T	-0.917	T	0.119	T	0.929	5.045	29.9	MALT_TH	TM_41	MALT_TH_	chr4:1052	2	TET2	-160	-462	NA	-390.091	-68297%	-97585%	NA	exonic	nonsynony	TET2:NM_	TRUE	.	rs759658003
MALT_TH	TM_41	MALT_TH_16_1621	chr4:105269703_C>T	3	TET2	616	178	NA	795	77%	22%	NA	exonic	nonsynony	TET2:NM_	TRUE	0.1%	rs5778108	ID=COSM8	.	.	.	.	.	0	D	1	D	1	D	.	.	1	D	2.82	M	2.55	T	-0.864	T	0.14	T	0.954	5.087	31	MALT_TH	TM_41	MALT_TH_	chr4:1052	3	TET2	-260	-698	NA	-529.443	-92683%	-132422%	NA	exonic	nonsynony	TET2:NM_	TRUE	100.1%	rs577810844
MALT_TH	TM_42	MALT_TH_16_1648	chr11:102328129_A>G	1	BIRC3	319	368	NA	687	46%	54%	NA	exonic	nonsynony	BIRC3:NM_	NA	.	.	.	.	.	.	.	.	0.12	T	0.708	P	0.411	B	0	D	1	D	2.245	M	1.96	T	-0.395	T	0.325	T	0.567	3.348	17.27	MALT_TH	TM_42	MALT_TH_	chr11:102	1	BIRC4	417	466	NA	-457.131	-80036%	-114360%	NA	exonic	nonsynony	BIRC3:NM_	NA	.	.
MALT_TH	TM_42	MALT_TH_16_1648	chr4:105269703_C>T	3	TET2	1171	246	NA	1418	83%	17%	NA	exonic	nonsynony	TET2:NM_	NA	0.1%	rs5778108	ID=COSM8	.	.	.	.	.	0	D	1	D	1	D	.	.	1	D	2.82	M	2.55	T	-0.864	T	0.14	T	0.954	5.087	31	MALT_TH	TM_42	MALT_TH_	chr4:1052	3	TET3	-679	-1604	NA	-944.827	-165374%	-236265%	NA	exonic	nonsynony	TET2:NM_	NA	100.1%	rs577810845
MALT_TH	TM_42	MALT_TH_16_1648	chr6:137871521_->GG	1	TNFAIP3	1339	152	NA	1491	90%	10%	NA	exonic	frameshift	TNFAIP3:N	NA	.	.	.	.	.	.	.	.	.	.	.	.	.	.	.	.	.	.	.	.	.	.	.	.	.	.	.	.	.	MALT_TH	TM_42	MALT_TH_	chr6:1378	1	TNFAIP4	-1035	-2222	NA	-993.565	-173901%	-248446%	NA	exonic	frameshift	TNFAIP3:N	NA	.	.
MALT_TH	TM_43	MALT_TH_16_1799	chr1:2556667_G>A	1	TNFRSF14	342	286	NA	629	54%	45%	NA	exonic	nonsynony	TNFRSF14:	NA	.	.	.	.	.	.	.	.	0	D	0.924	P	0.827	P	.	.	1	D	.	.	-2.2	D	0.303	D	0.713	D	0.828	2.591	14.62	MALT_TH	TM_43	MALT_TH_	chr1:2556	1	TNFRSF15	230	174	NA	-418.546	-73282%	-104709%	NA	exonic	nonsynony	TNFRSF14:	NA	.	.
MALT_TH	TM_43	MALT_TH_16_1799	chr7:2939861_A>G	1	CARD11	807	77	NA	889	91%	9%	NA	exonic	nonsynony	CARD11:NM	NA	.	.	ID=COSM4	.	.	.	.	.	0.29	T	0.23	B	0.05	B	0	D	1	D	1.845	L	1.09	T	-1.034	T	0.102	T	0.808	3.079	16.28	MALT_TH	TM_43	MALT_TH_	chr7:2939	1	CARD12	-653	-1383	NA	-592.249	-103671%	-148116%	NA	exonic	nonsynony	CARD11:NM	NA	.	.
MALT_TH	TM_43	MALT_TH_16_1799	chr4:105241345_T>-	1	TET2	259	96	NA	355	73%	27%	NA	exonic	frameshift	TET2:NM_	NA	.	.	.	.	.	.	.	.	.	.	.	.	.	.	.	.	.	.	.	.	.	.	.	.	.	.	.	.	.	MALT_TH	TM_43	MALT_TH_	chr4:1052	1	TET3	-67	-230	NA	-236.063	-41343%	-59079%	NA	exonic	frameshift	TET2:NM_	NA	.	.
MALT_TH	TM_43	MALT_TH_16_1799	chr9:5457362_C>G	1	CD274	1081	329	NA	1413	77%	23%	NA	exonic	stopgain	CD274:NM	NA	.	.	.	.	.	.	.	.	1	T	.	.	.	.	0	D	1	D	.	.	.	.	.	.	.	.	.	6.044	37	MALT_TH	TM_43	MALT_TH_	chr9:5457	1	CD275	-423	-1175	NA	-941.435	-164782%	-235420%	NA	exonic	stopgain	CD274:NM	NA	.	.
MALT_TH	TM_44	MALT_TH_16_1877	chr1:2557781_G>A	1	TNFRSF14	218	74	NA	292	75%	25%	NA	exonic	nonsynony	TNFRSF14:	NA	.	.	ID=COSM1	.	.	.	.	.	0	D	0.999	D	0.995	D	.	.	0.967	D	2.645	M	-1.26	T	0.258	D	0.595	D	0.56	1.815	12.03	MALT_TH	TM_44	MALT_TH_	chr1:2557	1	TNFRSF15	-70	-214	NA	-194.08	-33995%	-48583%	NA	exonic	nonsynony	TNFRSF14:	NA	.	.
MALT_TH	TM_44	MALT_TH_16_1877	chr14:100239402_ACGGCGGCGG1	2	YY1	805	320	NA	1125	72%	28%	NA	exonic	nonframes	YY1:NM_0	NA	.	rs7463234	.	.	.	.	.	.	.	.	.	.	.	.	.	.	.	.	.	.	.	.	.	.	.	.	.	.	.	MALT_TH	TM_44	MALT_TH_	chr14:1002	2	YY2	-165	-650	NA	-749.382	-131174%	-187410%	NA	exonic	nonframes	YY1:NM_0	NA	.	rs746323473
MALT_TH	TM_44	MALT_TH_16_1877	chr4:105243620_GC>-	1	TET2	366	175	NA	541	68%	32%	NA	exonic	frameshift	TET2:NM_	NA	.	.	.	.	.	.	.	.	.	.	.	.	.	.	.	.	.	.	.	.	.	.	.	.	.	.	.	.	.	MALT_TH	TM_44	MALT_TH_	chr4:1052	1	TET2	-16	-207	NA	-360.01	-63035%	-90069%	NA	exonic	frameshift	TET2:NM_	NA	.	.
MALT_TH	TM_44	MALT_TH_16_1877	chr4:105243757_G>A	1	TET2	612	94	NA	710	86%	13%	NA	exonic	nonsynony	TET2:NM_	NA	.	rs7717617	ID=COSM2	.	.	.	.	.	0	D	1	D	0.999	D	.	.	1	D	2.85	M	0.99	T	-0.292	T	0.332	T	0.968	4.643	25.5	MALT_TH	TM_44	MALT_TH_	chr4:1052	1	TET2	-424	-942	NA	-472.869	-82780%	-118274%	NA	exonic	nonsynony	TET2:NM_	NA	.	rs771761786
MALT_TH	TM_44	MALT_TH_16_1877	chr9:5457390_G>A	1	CD274	782	113	NA	897	87%	13%	NA	exonic	nonsynony	CD274:NM	NA	.	.	.	.	.	.	.	.	0.18	T	1	D	0.987	D	0	D	1	D	2.3	M	4.15	T	-1.193	T	0.037	T	0.062	3.916	19.92	MALT_TH	TM_44	MALT_TH_	chr9:5457	1	CD275	-556	-1225	NA	-597.541	-104598%	-149442%	NA	exonic	nonsynony	CD274:NM	NA	.	.
MALT_TH	TM_45	MALT_TH_16_391	chr4:105276219_GCATAAGAGCAT	1	TET2	1981	79	NA	2060	96%	4%	NA	exonic	frameshift	TET2:NM_	NA	.	.	.	.	.	.	.	.	.	.	.	.	.	.	.	.	.	.	.	.	.	.	.	.	.	.	.	.	.	MALT_TH	TM_45	MALT_TH_	chr4:1052	1	TET3	-1823	-3725	NA	-1372.96	-240294%	-343292%	NA	exonic	frameshift	TET2:NM_	NA	.	.
MALT_TH	TM_46	MALT_TH_16_501	chr1:2556666_T>C	3	TNFRSF14	290	26	NA	317	91%	8%	NA	exonic	nonsynony	TNFRSF14:	NA	.	rs7532686	.	.	.	.	.	.	0	D	0.967	D	0.879	P	.	.	1	D	.	.	-2.29	D	0.268	D	0.716	D	0.766	2.019	12.71	MALT_TH	TM_46	MALT_TH_	chr1:2556	3	TNFRSF14	-238	-502	NA	-210.919	-36938%	-52784%	NA	exonic	nonsynony	TNFRSF14:	NA	.	rs753268655
MALT_TH	TM_46	MALT_TH_16_501	chr1:2558411_T>G	1	TNFRSF14	1746	230	NA	1978	88%	12%	NA	exonic	nonsynony	TNFRSF14:	NA	.	.	.	.	.	.	.	.	0	D	1	D	1	D	.	.	0.792	N	2.935	M	-4.29	D	0.815	D	0.943	D	0.796	1.791	11.95	MALT_TH	TM_46	MALT_TH_	chr1:2558	1	TNFRSF14	-1286	-2802	NA	-1318.22	-230716%	-329610%	NA	exonic	nonsynony	TNFRSF14:	NA	.	.
MALT_TH	TM_46	MALT_TH_16_501	chr4:105269716_A>T	1	TET2	908	236	NA	1147	79%	21%	NA	exonic	nonsynony	TET2:NM_	NA	.	.	.	.	.	.	.	.	0	D	1	D	1	D	.	.	1	D	2.82	M	0.84	T	-0.203	T	0.356	T	0.965	4.707	26.1	MALT_TH	TM_46	MALT_TH_	chr4:1052	1	TET3	-436	-1108	NA	-764.128	-133753%	-191092%	NA	exonic	nonsynony	TET2:NM_	NA	.	.
MALT_TH	TM_46	MALT_TH_16_501	chr9:5456165_G>A	1	CD274	127	20	NA	147	86%	14%	NA	exonic	nonsynony	CD274:NM	NA	.	.	.	.	.	.	.	.	0	D	1	D	0.998	D	0	D	1	D	2.135	M	5.13	T	-0.274	T	0.377	T	0.66	4.423	23.5	MALT_TH	TM_46	MALT_TH_	chr9:5456	1	CD275	-87	-194	NA	-97.5306	-17096%	-24439%	NA	exonic	nonsynony	CD274:NM	NA	.	.
MALT_TH	TM_47	MALT_TH_16_541	chr19:16325964_G>C	2	KLF2	146	24	NA	170	86%	14%	NA	exonic	nonsynony	KLF2:NM_	NA	.	.	ID=COSM3	.	.	.	.	.	0.47	T	0.04	B	0.056	B	.	.	1	D	0.735	N	3.17	T	-0.973	T	0.015	T	0.125	-1.156	0.098	MALT_TH	TM_47	MALT_TH_	chr19:1632	2	KLF3	-98	-220	NA	-112.859	-19779%	-28272%	NA	exonic	nonsynony	KLF2:NM_	NA	.	.
MALT_TH	TM_19	MALT_TH_14_1315	chr17:45290744_A>G	1	MAP3K14	769	145	NA	915	84%	16%	NA	exonic	nonsynony	MAP3K14:	NA	.	.	.	.	.	.	.	.	0	D	0.967	D	0.916	D	0	D	0.993	N	.	.	.	.	-0.016	T	0.408	T	0.834	3.727	18.93	MALT_TH	TM_19	MALT_TH_	chr17:4529	1	MAP3K15	-479	-1103	NA	-609.509	-106693%	-152435%	NA	exonic	nonsynony	MAP3K14:	NA	.	.
MALT_TH	TM_47	MALT_TH_16_541	chr4:105234781_->T	1	TET2	905	147	NA	1052	86%	14%	NA	exonic	frameshift	TET2:NM_	NA	.	rs7624212	ID=COSM3	.	.	.	.	.	.	.	.	.	.	.	.	.	.	.	.	.	.	.	.	.	.	.	.	.	.	MALT_TH	TM_47	MALT_TH_	chr4:1052	1	TET2	-611	-1369	NA	-700.86	-122679%	-175272%	NA	exonic	frameshift	TET2:NM_	NA	.	rs762421228
MALT_TH	TM_47	MALT_TH_16_541	chr4:105234930_G>T	1	TET2	1317	431	NA	1755	75%	25%	NA	exonic	stopgain	TET2:NM_	NA	.	.	.	.	.	.	.	.	0.06	T	.	.	.	.	.	.	1	D	.	.	.	.	.	.	.	.	.	6.249	37	MALT_TH	TM_47	MALT_TH_	chr4:1052	1	TET2	-455	-1341	NA	-1169.42	-204680%	-292418%	NA	exonic	stopgain	TET2:NM_	NA	.	.
MALT_TH	TM_47	MALT_TH_16_541	chr6:137871301_C>-	1	TNFAIP3	377	80	NA	457	82%	18%	NA	exonic	frameshift	TNFAIP3:N	NA	.	.	.	.	.	.	.	.	.	.	.	.	.	.	.	.	.	.	.	.	.	.	.	.	.	.	.	.	.	MALT_TH	TM_47	MALT_TH_	chr6:1378	1	TNFAIP4	-217	-514	NA	-304.158	-53257%	-76098%	NA	exonic	frameshift	TNFAIP3:N	NA	.	.
MALT_TH	TM_47	MALT_TH_16_541	chr9:5465606_G>T	1	CD274	870	320	NA	1192	73%	27%	NA	exonic	nonsynony	CD274:NM	NA	.	.	.	.	.	.	.	.	0.02	D	0.985	D	0.602	P	.	.	1	N	1.39	L	5.17	T	-0.991	T	0.066	T	0.302	2.543	14.46	MALT_TH	TM_47	MALT_TH_	chr9:5465	1	CD275	-230	-780	NA	-794.065	-138993%	-198580%	NA	exonic	nonsynony	CD274:NM	NA	.	.
MALT_TH	TM_48	MALT_TH_16_679	chr1:2561671_A>C	2	TNFRSF14	169	169	NA	340	50%	50%	NA	splicing	.	.	TRUE	.	.	.	.	.	.	.	.	.	.	.	.	.	.	.	.	1	D	.	.	.	.	.	.	.	.	.	0.386	6.094	MALT_TH	TM_48	MALT_TH_	chr1:2561	2	TNFRSF15	169	169	NA	-225.838	-39559%	-56534%	NA	splicing	.	.	TRUE	.	.
MALT_TH	TM_48	MALT_TH_16_679	chr4:105235098_G>T	2	TET2	447	432	NA	882	51%	49%	NA	exonic	nonsynony	TET2:NM_	TRUE	.	.	.	.	.	.	.	.	0.07	T	0.932	P	0.415	B	0	U	1	N	0.805	L	4.4	T	-0.987	T	0.017	T	0.325	3.281	17.01	MALT_TH	TM_48	MALT_TH_	chr4:1052	2	TET3	417	402	NA	-587.178	-102793%	-146869%	NA	exonic	nonsynony	TET2:NM_	TRUE	.	.
MALT_TH	TM_48	MALT_TH_16_679	chr17:7673781_C>G	2	TP53	851	203	NA	1056	81%	19%	NA	exonic	nonsynony	TP53:NM_	TRUE	.	rs1219126	ID=COSM2	Uncertain	Malignant_	RCV00014	MedGen:C	C0376358:	0	D	1	D	1	D	0	D	1	D	3.33	M	-7.3	D	0.93	D	0.995	D	0.944	5.092	31	MALT_TH	TM_48	MALT_TH_	chr17:7673	2	TP54	-445	-1093	NA	-703.475	-123138%	-175928%	NA	exonic	nonsynony	TP53:NM_	TRUE	.	rs121912661
MALT_TH	TM_48	MALT_TH_16_679	chr9:5457197_G>A	1	CD274	40	10	NA	50	80%	20%	NA	exonic	stopgain	CD274:NM	FALSE	.	.	ID=COSM1	.	.	.	.	.	0.29	T	.	.	.	.	0	D	1	D	.	.	.	.	.	.	.	.	.	6.5	37	MALT_TH	TM_48	MALT_TH_	chr9:5457	1	CD275	-20	-50	NA	-32.8	-5770%	-8260%	NA	exonic	stopgain	CD274:NM	FALSE	.	.
MALT_TH	TM_49	MALT_TH_16_763	chr1:2557795_T>G	1	TNFRSF14	582	149	NA	735	79%	20%	NA	exonic	nonsynony	TNFRSF14:	NA	.	.	.	.	.	.	.	.	0	D	0.999	D	0.992	D	.	.	0.856	N	2.47	M	-0.71	T	-0.408	T	0.486	T	0.573	1.738	11.77	MALT_TH	TM_49	MALT_TH_	chr1:2557	1	TNFRSF15	-284	-717	NA	-489.466	-85686%	-122426%	NA	exonic	nonsynony	TNFRSF14:	NA	.	.
MALT_TH	TM_55	MALT_TH_17_1451	chr17:45286897_A>C	1	MAP3K14	2534	377	NA	2916	87%	13%	NA	exonic	nonsynony	MAP3K14:	NA	.	.	.	.	.	.	.	.	0.07	T	0.998	D	0.997	D	0	D	0.943	D	0.805	L	.	.	-0.533	T	0.22	T	.	3.58	18.24	MALT_TH	TM_55	MALT_TH_	chr17:4528	1	MAP3K15	-1780	-3937	NA	-1943.54	-340147%	-485941%	NA	exonic	nonsynony	MAP3K14:	NA	.	.
MALT_TH	TM_49	MALT_TH_16_763	chr16:3767869_T>C	1	CREBBP	657	153	NA	812	81%	19%	NA	exonic	nonsynony	CREBBP:NM	NA	.	.	.	.	.	.	.	.	0.19	T	0.998	D	0.979	D	0	N	1	D	2.015	M	-1.75	D	0.421	D	0.718	D	0.767	3.052	16.19	MALT_TH	TM_49	MALT_TH_	chr16:3767	1	CREBBP	-351	-855	NA	-540.812	-94672%	-135262%	NA	exonic	nonsynony	CREBBP:NM	NA	.	.
MALT_TH	TM_49	MALT_TH_16_763	chr16:3767911_T>C	1	CREBBP	2186	440	NA	2633	83%	17%	NA	splicing	.	.	NA	.	.	.	.	.	.	.	.	.	.	.	.	.	.	.	.	1	D	.	.	.	.	.	.	.	.	.	3.308	17.12	MALT_TH	TM_49	MALT_TH_	chr16:3767	1	CREBBP	-1306	-3052	NA	-1754.83	-307125%	-438767%	NA	splicing	.	.	NA	.	.
MALT_TH	TM_49	MALT_TH_16_763	chr4:105236436_->TT	1	TET2	420	125	NA	545	77%	23%	NA	exonic	frameshift	TET2:NM_	NA	.	.	.	.	.	.	.	.	.	.	.	.	.	.	.	.	.	.	.	.	.	.	.	.	.	.	.	.	.	MALT_TH	TM_49	MALT_TH_	chr4:1052	1	TET2	-170	-465	NA	-362.771	-63516%	-90754%	NA	exonic	frameshift	TET2:NM_	NA	.	.
MALT_TH	TM_49	MALT_TH_16_763	chr4:105275634_T>G	1	TET2	220	84	NA	305	72%	28%	NA	exonic	nonsynony	TET2:NM_	NA	.	.	.	.	.	.	.	.	0.34	T	0.92	P	0.631	P	.	.	1	D	1.245	L	4.37	T	-1.122	T	0.011	T	0.343	1.968	12.54	MALT_TH	TM_49	MALT_TH_	chr4:1052	1	TET2	-52	-188	NA	-202.726	-35509%	-50745%	NA	exonic	nonsynony	TET2:NM_	NA	.	.
MALT_TH	TM_49	MALT_TH_16_763	chr6:137876133_GTACCCTTGGTG	1	TNFAIP3	1847	172	NA	2019	91%	9%	NA	exonic	frameshift	TNFAIP3:N	NA	.	.	.	.	.	.	.	.	.	.	.	.	.	.	.	.	.	.	.	.	.	.	.	.	.	.	.	.	.	MALT_TH	TM_49	MALT_TH_	chr6:1378	1	TNFAIP4	-1503	-3178	NA	-1345.58	-235504%	-336450%	NA	exonic	frameshift	TNFAIP3:N	NA	.	.
MALT_TH	TM_50	MALT_TH_16_764	chr11:118894591_C>A	1	CXCR5	621	63	NA	684	91%	9%	NA	exonic	stopgain	CXCR5:NM	NA	.	.	.	.	.	.	.	.	1	T	.	.	.	.	0.157	U	1	D	.	.	.	.	.	.	.	.	.	4.864	27.8	MALT_TH	TM_50	MALT_TH_	chr11:1188	1	CXCR6	-495	-1053	NA	-455.575	-79753%	-113948%	NA	exonic	stopgain	CXCR5:NM	NA	.	.
MALT_TH	TM_50	MALT_TH_16_764	chr20:35631986_G>A	1	CPNE1	1304	1031	NA	2341	56%	44%	NA	exonic	nonsynony	CPNE1:NM	NA	.	rs3701021	.	.	.	.	.	.	0	D	1	D	0.997	D	0	U	0.999	D	2.905	M	1	T	0.071	D	0.5	T	0.525	2.89	15.63	MALT_TH	TM_50	MALT_TH_	chr20:3563	1	CPNE2	758	485	NA	-1559.89	-273017%	-390045%	NA	exonic	nonsynony	CPNE1:NM	NA	.	rs370102141
MALT_TH	TM_50	MALT_TH_16_764	chr18:62368987_C>T	1	TNFRSF11/	1170	153	NA	1324	88%	12%	NA	exonic	nonsynony	TNFRSF11/	NA	.	.	.	.	.	.	.	.	.	.	0.327	B	0.045	B	0.223	U	0.562	N	0	N	.	.	-1.019	T	0.053	T	0.071	1.902	12.32	MALT_TH	TM_50	MALT_TH_	chr18:6236	1	TNFRSF11/	-864	-1881	NA	-882.218	-154416%	-220610%	NA	exonic	nonsynony	TNFRSF11/	NA	.	.
MALT_TH	TM_50	MALT_TH_16_764	chr4:105272860_GTCAGCCCCATC	1	TET2	470	68	NA	538	87%	13%	NA	exonic	frameshift	TET2:NM_	NA	.	.	.	.	.	.	.	.	.	.	.	.	.	.	.	.	.	.	.	.	.	.	.	.	.	.	.	.	.	MALT_TH	TM_50	MALT_TH_	chr4:1052	1	TET3	-334	-736	NA	-358.207	-62714%	-89608%	NA	exonic	frameshift	TET2:NM_	NA	.	.
MALT_TH	TM_51	MALT_TH_17_1134	chr1:2559906_C>T	1	TNFRSF14	761	267	NA	1037	73%	26%	NA	exonic	stopgain	TNFRSF14:	NA	.	.	.	.	.	.	.	.	0.56	T	.	.	.	.	.	.	1	A	.	.	.	.	.	.	.	.	.	4.545	24.6	MALT_TH	TM_51	MALT_TH_	chr1:2559	1	TNFRSF15	-227	-721	NA	-690.745	-120912%	-172749%	NA	exonic	stopgain	TNFRSF14:	NA	.	.
MALT_TH	TM_51	MALT_TH_17_1134	chr4:105275943_T>A	1	TET2	1064	121	NA	1191	89%	10%	NA	exonic	stopgain	TET2:NM_	NA	.	.	.	.	.	.	.	.	0.23	T	.	.	.	.	.	.	1	N	.	.	.	.	.	.	.	.	.	13.387	47	MALT_TH	TM_51	MALT_TH_	chr4:1052	1	TET2	-822	-1765	NA	-793.567	-138902%	-198447%	NA	exonic	stopgain	TET2:NM_	NA	.	.
MALT_TH	TM_51	MALT_TH_17_1134	chr4:105276245_A>G	1	TET2	2153	421	NA	2587	83%	16%	NA	exonic	nonsynony	TET2:NM_	NA	.	.	.	.	.	.	.	.	0	D	1	D	0.997	D	.	.	1	D	2.465	M	3.37	T	-1.12	T	0.069	T	0.907	4.078	21	MALT_TH	TM_51	MALT_TH_	chr4:1052	1	TET2	-1311	-3043	NA	-1724.17	-301759%	-431101%	NA	exonic	nonsynony	TET2:NM_	NA	.	.
MALT_TH	TM_51	MALT_TH_17_1134	chr6:137877235_CTCAT>-	1	TNFAIP3	1409	397	NA	1806	78%	22%	NA	exonic	frameshift	TNFAIP3:N	NA	.	.	.	.	.	.	.	.	.	.	.	.	.	.	.	.	.	.	.	.	.	.	.	.	.	.	.	.	.	MALT_TH	TM_51	MALT_TH_	chr6:1378	1	TNFAIP4	-615	-1627	NA	-1203.45	-210634%	-300923%	NA	exonic	frameshift	TNFAIP3:N	NA	.	.
MALT_TH	TM_51	MALT_TH_17_1134	chr9:5456152_G>A	1	CD274	77	28	NA	105	73%	27%	NA	exonic	stopgain	CD274:NM	NA	.	.	.	.	.	.	.	.	1	T	.	.	.	.	0.295	N	1	A	.	.	.	.	.	.	.	.	.	5.238	33	MALT_TH	TM_51	MALT_TH_	chr9:5456	1	CD275	-21	-70	NA	-69.4	-12177%	-17413%	NA	exonic	stopgain	CD274:NM	NA	.	.
MALT_TH	TM_52	MALT_TH_17_1380	chr1:2558464_C>-	1	TNFRSF14	1753	756	NA	2509	70%	30%	NA	exonic	frameshift	TNFRSF14:	NA	.	.	.	.	.	.	.	.	.	.	.	.	.	.	.	.	.	.	.	.	.	.	.	.	.	.	.	.	.	MALT_TH	TM_52	MALT_TH_	chr1:2558	1	TNFRSF15	-241	-1238	NA	-1672.03	-292638%	-418073%	NA	exonic	frameshift	TNFRSF14:	NA	.	.
MALT_TH	TM_52	MALT_TH_17_1380	chr3:32954122_A>C	1	CCR4	1737	502	NA	2240	78%	22%	NA	exonic	nonsynony	CCR4:NM_	NA	.	.	.	.	.	.	.	.	0	D	0.879	P	0.797	P	0	D	1	N	1.99	M	1.18	T	-0.869	T	0.164	T	0.192	1.898	12.3	MALT_TH	TM_52	MALT_TH_	chr3:3295	1	CCR5	-733	-1968	NA	-1492.78	-261266%	-373255%	NA	exonic	nonsynony	CCR4:NM_	NA	.	.
MALT_TH	TM_52	MALT_TH_17_1380	chr4:105243568_A>G	1	TET2	582	803	NA	1391	42%	58%	NA	splicing	.	.	NA	.	.	.	.	.	.	.	.	.	.	.	.	.	.	.	.	1	D	.	.	.	.	.	.	.	.	.	3.494	17.87	MALT_TH	TM_52	MALT_TH_	chr4:1052	1	TET3	1024	1245	NA	-926.424	-162164%	-231685%	NA	splicing	.	.	NA	.	.
MALT_TH	TM_53	MALT_TH_17_1385	chr1:2556665_A>G	1	TNFRSF14	531	159	NA	691	77%	23%	NA	exonic	nonsynony	TNFRSF14:	NA	.	.	.	.	.	.	.	.	0	D	0.924	P	0.827	P	.	.	1	D	.	.	-2.2	D	0.316	D	0.675	D	0.831	2.731	15.09	MALT_TH	TM_53	MALT_TH_	chr1:2556	1	TNFRSF14	-213	-585	NA	-460.104	-80549%	-115087%	NA	exonic	nonsynony	TNFRSF14:	NA	.	.
MALT_TH	TM_53	MALT_TH_17_1385	chr1:2558388_G>A	1	TNFRSF14	1906	621	NA	2530	75%	25%	NA	exonic	nonsynony	TNFRSF14:	NA	.	.	.	.	.	.	.	.	0	D	1	D	0.999	D	.	.	1	N	2.645	M	-3.92	D	0.511	D	0.914	D	0.868	2.678	14.92	MALT_TH	TM_53	MALT_TH_	chr1:2558	1	TNFRSF14	-664	-1949	NA	-1686.09	-295097%	-421584%	NA	exonic	nonsynony	TNFRSF14:	NA	.	.
MALT_TH	TM_53	MALT_TH_17_1385	chr17:40554915_T>C	1	CCR7	1384	417	NA	1805	77%	23%	NA	exonic	nonsynony	CCR7:NM_	NA	.	.	.	.	.	.	.	.	0.01	D	0.994	D	0.99	D	0	D	1	D	3.34	M	-1.79	D	0.773	D	0.78	D	0.6	4.69	25.9	MALT_TH	TM_53	MALT_TH_	chr17:4055	1	CCR8	-550	-1517	NA	-1202.77	-210515%	-300754%	NA	exonic	nonsynony	CCR7:NM_	NA	.	.
MALT_TH	TM_53	MALT_TH_17_1385	chr4:105236002_C>-	1	TET2	691	222	NA	913	76%	24%	NA	exonic	frameshift	TET2:NM_	NA	.	.	.	.	.	.	.	.	.	.	.	.	.	.	.	.	.	.	.	.	.	.	.	.	.	.	.	.	.	MALT_TH	TM_53	MALT_TH_	chr4:1052	1	TET3	-247	-716	NA	-608.09	-106447%	-152085%	NA	exonic	frameshift	TET2:NM_	NA	.	.
MALT_TH	TM_53	MALT_TH_17_1385	chr14:100239863_C>T	1	YY1	989	258	NA	1249	79%	21%	NA	exonic	stopgain	YY1:NM_0	NA	.	.	.	.	.	.	.	.	0.47	T	.	.	.	.	0	U	1	A	.	.	.	.	.	.	.	.	.	4.288	22.4	MALT_TH	TM_53	MALT_TH_	chr14:1002	1	YY2	-473	-1204	NA	-832.127	-145652%	-208092%	NA	exonic	stopgain	YY1:NM_0	NA	.	.
MALT_TH	TM_53	MALT_TH_17_1385	chr4:105269664_C>T	1	TET2	497	200	NA	703	71%	28%	NA	exonic	nonsynony	TET2:NM_	NA	.	.	.	.	.	.	.	.	0	D	1	D	1	D	.	.	1	D	2.82	M	2.45	T	-0.792	T	0.165	T	0.964	5.22	33	MALT_TH	TM_53	MALT_TH_	chr4:1052	1	TET3	-97	-394	NA	-468.052	-81941%	-117077%	NA	exonic	nonsynony	TET2:NM_	NA	.	.
MALT_TH	TM_53	MALT_TH_17_1385	chr6:167137261_C>A	1	CCR6	1439	352	NA	1793	80%	20%	NA	exonic	stopgain	CCR6:NM_	NA	.	.	.	.	.	.	.	.	0.41	T	.	.	.	.	0.06	U	1	D	.	.	.	.	.	.	.	.	.	5.276	33	MALT_TH	TM_53	MALT_TH_	chr6:1671	1	CCR7	-735	-1822	NA	-1194.8	-209121%	-298761%	NA	exonic	stopgain	CCR6:NM_	NA	.	.
MALT_TH	TM_53	MALT_TH_17_1385	chr9:5457286_T>C	1	CD274	1447	259	NA	1712	85%	15%	NA	exonic	nonsynony	CD274:NM	NA	.	.	.	.	.	.	.	.	0	D	1	D	1	D	0.015	N	0.951	N	2.995	M	-0.43	T	-0.323	T	0.515	D	0.934	1.477	10.88	MALT_TH	TM_53	MALT_TH_	chr9:5457	1	CD274	-929	-2117	NA	-1140.85	-199677%	-285270%	NA	exonic	nonsynony	CD274:NM	NA	.	.
MALT_TH	TM_53	MALT_TH_17_1385	chr9:5463019_A>C	1	CD274	3282	994	NA	4282	77%	23%	NA	exonic	nonsynony	CD274:NM	NA	.	.	.	.	.	.	.	.	0	D	1	D	1	D	0	D	0.999	D	2.815	M	-1.16	T	0.472	D	0.666	D	0.915	3.924	19.96	MALT_TH	TM_53	MALT_TH_	chr9:5463	1	CD274	-1294	-3582	NA	-2854.1	-499499%	-713587%	NA	exonic	nonsynony	CD274:NM	NA	.	.
MALT_TH	TM_54	MALT_TH_17_1440	chr1:2556667_G>A	2	TNFRSF14	249	56	NA	306	81%	18%	NA	exonic	nonsynony	TNFRSF14:	TRUE	.	.	.	.	.	.	.	.	0	D	0.924	P	0.827	P	.	.	1	D	.	.	-2.2	D	0.303	D	0.713	D	0.828	2.591	14.62	MALT_TH	TM_54	MALT_TH_	chr1:2556	2	TNFRSF15	-137	-330	NA	-203.485	-35639%	-50930%	NA	exonic	nonsynony	TNFRSF14:	TRUE	.	.
MALT_TH	TM_54	MALT_TH_17_1440	chr19:16326917_G>C	2	KLF2	548	72	NA	623	88%	12%	NA	exonic	nonsynony	KLF2:NM_	TRUE	.	.	.	.	.	.	.	.	0	D	1	D	0.999	D	.	.	1	N	-0.26	N	0.75	T	-0.921	T	0.121	T	0.407	2.616	14.71	MALT_TH	TM_54	MALT_TH_	chr19:1632	2	KLF3	-404	-880	NA	-414.886	-72633%	-103777%	NA	exonic	nonsynony	KLF2:NM_	TRUE	.	.
MALT_TH	TM_54	MALT_TH_17_1440	chr4:105236024_->A	2	TET2	221	31	NA	252	88%	12%	NA	exonic	frameshift	TET2:NM_	TRUE	.	.	.	.	.	.	.	.	.	.	.	.	.	.	.	.	.	.	.	.	.	.	.	.	.	.	.	.	.	MALT_TH	TM_54	MALT_TH_	chr4:1052	2	TET2	-159	-349	NA	-167.544	-29348%	-41942%	NA	exonic	frameshift	TET2:NM_	TRUE	.	.
MALT_TH	TM_54	MALT_TH_17_1440	chr4:105236454_AC>-	2	TET2	278	63	NA	341	82%	18%	NA	exonic	frameshift	TET2:NM_	TRUE	.	.	.	.	.	.	.	.	.	.	.	.	.	.	.	.	.	.	.	.	.	.	.	.	.	.	.	.	.	MALT_TH	TM_54	MALT_TH_	chr4:1052	2	TET2	-152	-367	NA	-226.815	-39722%	-56763%	NA	exonic	frameshift	TET2:NM_	TRUE	.	.
MALT_TH	TM_54	MALT_TH_17_1440	chr9:5457276_->G	2	CD274	212	90	NA	302	70%	30%	NA	exonic	frameshift	CD274:NM	TRUE	.	.	.	.	.	.	.	.	.	.	.	.	.	.	.	.	.	.	.	.	.	.	.	.	.	.	.	.	.	MALT_TH	TM_54	MALT_TH_	chr9:5457	2	CD275	-32	-154	NA	-200.702	-35155%	-50240%	NA	exonic	frameshift	CD274:NM	TRUE	.	.
MALT_TH	TM_55	MALT_TH_17_1451	chr1:2558432_->TAAT	1	TNFRSF14	2229	548	NA	2777	80%	20%	NA	exonic	frameshift	TNFRSF14:	NA	.	.	.	.	.	.	.	.	.	.	.	.	.	.	.	.	.	.	.	.	.	.	.	.	.	.	.	.	.	MALT_TH	TM_55	MALT_TH_	chr1:2558	1	TNFRSF15	-1133	-2814	NA	-1850.8	-323920%	-462761%	NA	exonic	frameshift	TNFRSF14:	NA	.	.
MALT_TH	TM_55	MALT_TH_17_1451	chr11:27369069_C>T	1	LGR4	251	248	NA	500	50%	50%	NA	exonic	nonsynony	LGR4:NM_	NA	.	.	.	.	.	.	.	.	0.03	D	0.91	P	0.495	P	0	D	1	D	2.515	M	-2.44	D	0.573	D	0.74	D	0.587	4.011	20.6	MALT_TH	TM_55	MALT_TH_	chr11:2736	1	LGR5	245	242	NA	-332.505	-58226%	-83201%	NA	exonic	nonsynony	LGR4:NM_	NA	.	.
MALT_TH	TM_55	MALT_TH_17_1451	chr3:177033044_A>T	1	TBL1XR1	961	155	NA	1119	86%	14%	NA	exonic	nonsynony	TBL1XR1:N	NA	.	.	.	.	.	.	.	.	0	D	0.981	D	0.94	D	0	D	1	D	3.975	H	-0.23	T	0.543	D	0.619	D	0.983	4.851	27.7	MALT_TH	TM_55	MALT_TH_	chr3:1770	1	TBL1XR1	-651	-1457	NA	-745.529	-130496%	-186439%	NA	exonic	nonsynony	TBL1XR1:N	NA	.	.
MALT_TH	TM_55	MALT_TH_17_1451	chr3:177033051_A>G	1	TBL1XR1	2657	350	NA	3008	88%	12%	NA	exonic	nonsynony	TBL1XR1:N	NA	.	.	.	.	.	.	.	.	0.11	T	1	D	0.997	D	0	D	1	D	0.875	L	0.18	T	-0.584	T	0.271	T	0.854	4.704	26.1	MALT_TH	TM_55	MALT_TH_	chr3:1770	1	TBL1XR1	-1957	-4264	NA	-2004.88	-350883%	-501277%	NA	exonic	nonsynony	TBL1XR1:N	NA	.	.
MALT_TH	TM_55	MALT_TH_17_1451	chr2:174446729_C>T	1	GPR155	2842	212	NA	3059	93%	7%	NA	exonic	nonsynony	GPR155:NM	NA	.	rs5755553	ID=COSM1	.	.	.	.	.	0.21	T	0.011	B	0.007	B	0.011	N	0.92	D	1.245	L	0.88	T	-1.029	T	0.08	T	0.152	2.089	12.94	MALT_TH	TM_55	MALT_TH_	chr2:1744	1	GPR156	-2418	-5048	NA	-2038.93	-356840%	-509786%	NA	exonic	nonsynony	GPR155:NM	NA	.	rs575555341
MALT_TH	TM_55	MALT_TH_17_1451	chr4:105235323_C>T	1	TET2	3453	630	NA	4091	84%	15%	NA	exonic	stopgain	TET2:NM_	NA	.	.	.	.	.	.	.	.	0.18	T	.	.	.	.	0	U	1	D	.	.	.	.	.	.	.	.	.	6.886	38	MALT_TH	TM_55	MALT_TH_	chr4:1052	1	TET3	-2193	-5016	NA	-2726.85	-477227%	-681769%	NA	exonic	stopgain	TET2:NM_	NA	.	.
MALT_TH	TM_18	MALT_TH_14_1279	chr3:46021569_G>A	1	XCR1	473	509	NA	982	48%	52%	NA	exonic	nonsynony	XCR1:NM_	NA	.	rs1513375	.	.	.	.	.	.	0	D	1	D	1	D	0	D	1	D	4.125	H	-4.28	D	1.085	D	0.969	D	0.964	4.192	21.7	MALT_TH	TM_18	MALT_TH_	chr3:4602	1	XCR2	545	581	NA	-653.815	-114456%	-163530%	NA	exonic	nonsynony	XCR1:NM_	NA	.	rs151337543
MALT_TH	TM_55	MALT_TH_17_1451	chr9:5456116_G>T	3	CD274	147	26	NA	173	85%	15%	NA	exonic	nonsynony	CD274:NM	NA	.	.	.	.	.	.	.	.	0	D	0.993	D	0.971	D	0	D	0.937	N	.	.	5.21	T	-0.828	T	0.214	T	0.932	2.369	13.88	MALT_TH	TM_55	MALT_TH_	chr9:5456	3	CD275	-95	-216	NA	-114.85	-20127%	-28770%	NA	exonic	nonsynony	CD274:NM	NA	.	.
MALT_TH	TM_56	MALT_TH_17_1615	chr4:105275896_G>-	1	TET2	745	187	NA	932	80%	20%	NA	exonic	frameshift	TET2:NM_	NA	.	.	.	.	.	.	.	.	.	.	.	.	.	.	.	.	.	.	.	.	.	.	.	.	.	.	.	.	.	MALT_TH	TM_56	MALT_TH_	chr4:1052	1	TET3	-371	-929	NA	-620.799	-108670%	-155260%	NA	exonic	frameshift	TET2:NM_	NA	.	.
MALT_TH	TM_56	MALT_TH_17_1615	chr6:137875700_G>T	1	TNFAIP3	1500	377	NA	1881	80%	20%	NA	exonic	stopgain	TNFAIP3:N	NA	.	.	.	.	.	.	.	.	0	D	.	.	.	.	0	D	1	A	.	.	.	.	.	.	.	.	.	7.531	39	MALT_TH	TM_56	MALT_TH_	chr6:1378	1	TNFAIP3	-746	-1869	NA	-1253.47	-219387%	-313427%	NA	exonic	stopgain	TNFAIP3:N	NA	.	.
MALT_TH	TM_56	MALT_TH_17_1615	chr6:137875748_C>T	1	TNFAIP3	1896	224	NA	2122	89%	11%	NA	exonic	stopgain	TNFAIP3:N	NA	.	.	ID=COSM3	.	.	.	.	.	1	T	.	.	.	.	0.195	N	1	A	.	.	.	.	.	.	.	.	.	5.18	32	MALT_TH	TM_56	MALT_TH_	chr6:1378	1	TNFAIP3	-1448	-3120	NA	-1414.23	-247518%	-353612%	NA	exonic	stopgain	TNFAIP3:N	NA	.	.
MALT_TH	TM_56	MALT_TH_17_1615	chr9:5462840_A>G	1	CD274	1460	920	NA	2391	61%	38%	NA	exonic	nonsynony	CD274:NM	NA	.	.	.	.	.	.	.	.	0	D	1	D	1	D	0	D	0.999	D	2.885	M	5.09	T	-0.247	T	0.35	T	0.868	2.364	13.86	MALT_TH	TM_56	MALT_TH_	chr9:5462	1	CD275	380	-160	NA	-1593.28	-278859%	-398390%	NA	exonic	nonsynony	CD274:NM	NA	.	.
MALT_TH	TM_57	MALT_TH_17_59	chr4:105243760_G>-	2	TET2	162	37	NA	199	81%	19%	NA	exonic	frameshift	TET2:NM_	TRUE	.	.	.	.	.	.	.	.	.	.	.	.	.	.	.	.	.	.	.	.	.	.	.	.	.	.	.	.	.	MALT_TH	TM_57	MALT_TH_	chr4:1052	2	TET3	-88	-213	NA	-132.147	-23155%	-33096%	NA	exonic	frameshift	TET2:NM_	TRUE	.	.
MALT_TH	TM_57	MALT_TH_17_59	chr22:41131558_C>T	2	EP300	752	107	NA	859	88%	12%	NA	exonic	stopgain	EP300:NM	TRUE	.	.	.	.	.	.	.	.	0.35	T	.	.	.	.	0.017	N	1	A	.	.	.	.	.	.	.	.	.	14.938	49	MALT_TH	TM_57	MALT_TH_	chr22:4113	2	EP301	-538	-1183	NA	-572.209	-100165%	-143108%	NA	exonic	stopgain	EP300:NM	TRUE	.	.
MALT_TH	TM_57	MALT_TH_17_59	chr4:105272858_A>T	2	TET2	160	24	NA	184	87%	13%	NA	exonic	stopgain	TET2:NM_	TRUE	.	.	.	.	.	.	.	.	1	T	.	.	.	.	.	.	1	D	.	.	.	.	.	.	.	.	.	16.701	50	MALT_TH	TM_57	MALT_TH_	chr4:1052	2	TET3	-112	-248	NA	-122.203	-21414%	-30607%	NA	exonic	stopgain	TET2:NM_	TRUE	.	.
MALT_TH	TM_57	MALT_TH_17_59	chr9:5457368_C>A	2	CD274	453	346	NA	799	57%	43%	NA	exonic	stopgain	CD274:NM	TRUE	.	.	.	.	.	.	.	.	1	T	.	.	.	.	0	D	1	D	.	.	.	.	.	.	.	.	.	5.888	36	MALT_TH	TM_57	MALT_TH_	chr9:5457	2	CD275	239	132	NA	-531.9	-93118%	-133047%	NA	exonic	stopgain	CD274:NM	TRUE	.	.
MALT_TH	TM_58	MALT_TH_17_666	chr1:2556667_G>C	1	TNFRSF14	316	67	NA	385	82%	17%	NA	exonic	nonsynony	TNFRSF14:	FALSE	.	.	.	.	.	.	.	.	0	D	0.924	P	0.827	P	.	.	1	D	.	.	-2.2	D	0.303	D	0.713	D	0.828	2.47	14.22	MALT_TH	TM_58	MALT_TH_	chr1:2556	1	TNFRSF15	-182	-431	NA	-256.161	-44857%	-64099%	NA	exonic	nonsynony	TNFRSF14:	FALSE	.	.
MALT_TH	TM_58	MALT_TH_17_666	chr12:49037221_G>A	2	KMT2D	2372	425	NA	2797	85%	15%	NA	exonic	stopgain	KMT2D:NM	TRUE	.	.	.	.	.	.	.	.	0	D	.	.	.	.	0.003	N	1	A	.	.	.	.	.	.	.	.	.	18.184	51	MALT_TH	TM_58	MALT_TH_	chr12:4903	2	KMT2D	-1522	-3469	NA	-1864.18	-326261%	-466103%	NA	exonic	stopgain	KMT2D:NM	TRUE	.	.
MALT_TH	TM_58	MALT_TH_17_666	chr19:16325264_A>C	2	KLF2	464	68	NA	534	87%	13%	NA	exonic	nonsynony	KLF2:NM_	TRUE	.	.	.	.	.	.	.	.	0.07	T	0.989	D	0.648	P	.	.	0.926	N	1.735	L	2.94	T	-1.056	T	0.038	T	0.04	1.122	9.574	MALT_TH	TM_58	MALT_TH_	chr19:1632	2	KLF2	-328	-724	NA	-355.541	-62248%	-88941%	NA	exonic	nonsynony	KLF2:NM_	TRUE	.	.
MALT_TH	TM_58	MALT_TH_17_666	chr19:16326917_G>C	2	KLF2	723	111	NA	836	86%	13%	NA	exonic	nonsynony	KLF2:NM_	TRUE	.	.	.	.	.	.	.	.	0	D	1	D	0.999	D	.	.	1	N	-0.26	N	0.75	T	-0.921	T	0.121	T	0.407	2.616	14.71	MALT_TH	TM_58	MALT_TH_	chr19:1632	2	KLF2	-501	-1113	NA	-556.868	-97480%	-139274%	NA	exonic	nonsynony	KLF2:NM_	TRUE	.	.
MALT_TH	TM_58	MALT_TH_17_666	chr4:105272645_A>-	2	TET2	2165	1144	NA	3311	65%	35%	NA	exonic	frameshift	TET2:NM_	TRUE	.	.	.	.	.	.	.	.	.	.	.	.	.	.	.	.	.	.	.	.	.	.	.	.	.	.	.	.	.	MALT_TH	TM_58	MALT_TH_	chr4:1052	2	TET3	123	-898	NA	-2206.65	-386198%	-551731%	NA	exonic	frameshift	TET2:NM_	TRUE	.	.
MALT_TH	TM_58	MALT_TH_17_666	chr6:137877171_A>-	2	TNFAIP3	1099	101	NA	1200	92%	8%	NA	exonic	frameshift	TNFAIP3:N	TRUE	.	.	.	.	.	.	.	.	.	.	.	.	.	.	.	.	.	.	.	.	.	.	.	.	.	.	.	.	.	MALT_TH	TM_58	MALT_TH_	chr6:1378	2	TNFAIP4	-897	-1895	NA	-799.583	-139954%	-199950%	NA	exonic	frameshift	TNFAIP3:N	TRUE	.	.
MALT_TH	TM_59	MALT_TH_17_785	chr7:2937187_T>A	1	CARD11	2388	365	NA	2757	87%	13%	NA	exonic	nonsynony	CARD11:NM	NA	.	.	.	.	.	.	.	.	0.11	T	1	D	0.999	D	0	D	0.546	N	1.365	L	1.25	T	-0.838	T	0.108	T	0.66	3.369	17.35	MALT_TH	TM_59	MALT_TH_	chr7:2937	1	CARD12	-1658	-3681	NA	-1837.53	-321597%	-459440%	NA	exonic	nonsynony	CARD11:NM	NA	.	.

MALT_TH	TM_60	MALT_TH_17_831	chr1:2556666_T>C	1	TNFRSF14	616	126	NA	742	83%	17%	NA	exonic	nonsynony	TNFRSF14:	NA	.	rs7532686	.	.	.	.	.	.	0	D	0.967	D	0.879	P	.	.	1	D	.	.	-2.29	D	0.268	D	0.716	D	0.766	2.019	12.71	MALT_TH	TM_60	MALT_TH_	chr1:2556	1	TNFRSF15	-364	-854	NA	-494.164	-86508%	-123599%	NA	exonic	nonsynony	TNFRSF14:	NA	.	rs753268655
MALT_TH	TM_60	MALT_TH_17_831	chr4:105235059_C>T	1	TET2	1644	293	NA	1939	85%	15%	NA	exonic	stopgain	TET2:NM_	NA	.	.	.	.	.	.	.	.	0.02	D	.	.	.	.	0.002	N	1	D	.	.	.	.	.	.	.	.	.	6.981	38	MALT_TH	TM_60	MALT_TH_	chr4:1052	1	TET3	-1058	-2409	NA	-1292.18	-226161%	-323103%	NA	exonic	stopgain	TET2:NM_	NA	.	.
MALT_TH	TM_60	MALT_TH_17_831	chr6:111411501_T>C	1	REV3L	142	98	NA	240	59%	41%	NA	exonic	nonsynony	REV3L:NM	NA	.	.	.	.	.	.	.	.	0.01	D	1	D	1	D	0	D	1	D	2.905	M	2.99	T	-0.343	T	0.293	T	0.866	4.369	23.1	MALT_TH	TM_60	MALT_TH_	chr6:1114	1	REV3L	54	10	NA	-159.258	-27905%	-39885%	NA	exonic	nonsynony	REV3L:NM	NA	.	.
MALT_TH	TM_60	MALT_TH_17_831	chr4:105276017_TGGCT>-	1	TET2	3391	291	NA	3682	92%	8%	NA	exonic	frameshift	TET2:NM_	NA	.	.	.	.	.	.	.	.	.	.	.	.	.	.	.	.	.	.	.	.	.	.	.	.	.	.	.	.	.	MALT_TH	TM_60	MALT_TH_	chr4:1052	1	TET3	-2809	-5909	NA	-2454.25	-429521%	-613618%	NA	exonic	frameshift	TET2:NM_	NA	.	.
MALT_TH	TM_60	MALT_TH_17_831	chr9:5456167_T>A	1	CD274	126	16	NA	142	89%	11%	NA	splicing	.	.	NA	.	.	.	.	.	.	.	.	.	.	.	.	.	.	.	.	1	D	.	.	.	.	.	.	.	.	.	2.98	15.94	MALT_TH	TM_60	MALT_TH_	chr9:5456	1	CD274	-94	-204	NA	-94.2207	-16516%	-23611%	NA	splicing	.	.	NA	.	.
MALT_TH	TM_60	MALT_TH_17_831	chr9:5463122_G>C	1	CD274	276	27	NA	304	91%	9%	NA	exonic;spli	nonsynony	CD274:NM	NA	.	.	.	.	.	.	.	.	.	.	.	.	.	.	.	.	1	D	.	.	.	.	.	.	.	.	.	3.552	18.12	MALT_TH	TM_60	MALT_TH_	chr9:5463	1	CD274	-222	-471	NA	-202.246	-35420%	-50616%	NA	exonic;spli	nonsynony	CD274:NM	NA	.	.
MALT_TH	TM_61	MALT_TH_17_902	chr1:2558381_A>G	1	TNFRSF14	1377	635	NA	2016	68%	31%	NA	exonic	nonsynony	TNFRSF14:	NA	.	.	.	.	.	.	.	.	0.01	D	0.993	D	0.978	D	.	.	1	N	2.505	M	-0.54	T	-0.567	T	0.477	T	0.263	2.778	15.25	MALT_TH	TM_61	MALT_TH_	chr1:2558	1	TNFRSF15	-107	-849	NA	-1343.35	-235119%	-335904%	NA	exonic	nonsynony	TNFRSF14:	NA	.	.
MALT_TH	TM_61	MALT_TH_17_902	chr4:105243756_C>T	1	TET2	706	429	NA	1144	62%	38%	NA	exonic	nonsynony	TET2:NM_	NA	.	.	ID=COSM8	.	.	.	.	.	0	D	1	D	1	D	.	.	1	D	2.85	M	0.97	T	-0.292	T	0.332	T	0.959	3.494	17.87	MALT_TH	TM_61	MALT_TH_	chr4:1052	1	TET3	152	-125	NA	-761.961	-133377%	-190559%	NA	exonic	nonsynony	TET2:NM_	NA	.	.
MALT_TH	TM_61	MALT_TH_17_902	chr7:2944249_TTC>-	1	CARD11	819	97	NA	916	89%	11%	NA	exonic	nonframes	CARD11:N	NA	.	.	ID=COSM4	.	.	.	.	.	.	.	.	.	.	.	.	.	.	.	.	.	.	.	.	.	.	.	.	.	.	MALT_TH	TM_61	MALT_TH_	chr7:2944	1	CARD12	-625	-1347	NA	-610.227	-106817%	-152612%	NA	exonic	nonframes	CARD11:N	NA	.	.
MALT_TH	TM_61	MALT_TH_17_902	chr9:5456116_G>A	1	CD274	108	16	NA	124	87%	13%	NA	exonic	nonsynony	CD274:NM	NA	.	rs1114012	.	.	.	.	.	.	0	D	0.993	D	0.971	D	0	D	0.926	N	.	.	5.21	T	-0.828	T	0.214	T	0.932	2.429	14.08	MALT_TH	TM_61	MALT_TH_	chr9:5456	1	CD275	-76	-168	NA	-82.2043	-14414%	-20608%	NA	exonic	nonsynony	CD274:NM	NA	.	rs111401208
MALT_TH	TM_62	MALT_TH_17_981	chr1:2557817_G>T	1	TNFRSF14	773	107	NA	881	88%	12%	NA	exonic	nonsynony	TNFRSF14:	NA	.	.	.	.	.	.	.	.	0.01	D	1	D	0.999	D	.	.	0.996	D	2.28	M	-0.98	T	0.029	D	0.52	D	0.491	1.579	11.24	MALT_TH	TM_62	MALT_TH_	chr1:2557	1	TNFRSF14	-559	-1225	NA	-586.879	-102732%	-146776%	NA	exonic	nonsynony	TNFRSF14:	NA	.	.
MALT_TH	TM_63	MALT_TH_1711_2210B	chr1:2556666_T>A	1	TNFRSF14	189	47	NA	237	80%	20%	NA	exonic	nonsynony	TNFRSF14:	FALSE	.	.	.	.	.	.	.	.	0	D	0.967	D	0.879	P	.	.	1	D	.	.	-2.29	D	0.268	D	0.716	D	0.734	2.573	14.56	MALT_TH	TM_63	MALT_TH_	chr1:2556	1	TNFRSF14	-95	-237	NA	-157.47	-27587%	-39427%	NA	exonic	nonsynony	TNFRSF14:	FALSE	.	.
MALT_TH	TM_63	MALT_TH_1711_2210B	chr9:5463064_T>C	2	CD274	1009	180	NA	1189	85%	15%	NA	exonic	nonsynony	CD274:NM	TRUE	.	.	.	.	.	.	.	.	0	D	1	D	1	D	0	D	1	D	2.9	M	0.34	T	-0.141	T	0.392	T	0.997	3.625	18.44	MALT_TH	TM_63	MALT_TH_	chr9:5463	2	CD275	-649	-1478	NA	-792.182	-138661%	-198103%	NA	exonic	nonsynony	CD274:NM	TRUE	.	.
MALT_TH	TM_64	MALT_TH_18_1063	chr3:177038387_AAG>-	1	TBL1XR1	798	82	NA	880	91%	9%	NA	exonic	nonframes	TBL1XR1:N	NA	.	.	.	.	.	.	.	.	.	.	.	.	.	.	.	.	.	.	.	.	.	.	.	.	.	.	.	.	.	MALT_TH	TM_64	MALT_TH_	chr3:1770	1	TBL1XR2	-634	-1350	NA	-586.24	-102619%	-146615%	NA	exonic	nonframes	TBL1XR1:N	NA	.	.
MALT_TH	TM_64	MALT_TH_18_1063	chr4:105235452_A>-	1	TET2	3664	474	NA	4138	89%	11%	NA	exonic	frameshift	TET2:NM_	NA	.	.	.	.	.	.	.	.	.	.	.	.	.	.	.	.	.	.	.	.	.	.	.	.	.	.	.	.	.	MALT_TH	TM_64	MALT_TH_	chr4:1052	1	TET3	-2716	-5906	NA	-2758.22	-482716%	-689610%	NA	exonic	frameshift	TET2:NM_	NA	.	.
MALT_TH	TM_64	MALT_TH_18_1063	chr3:183492154_C>T	1	KLHL6	1836	182	NA	2028	91%	9%	NA	exonic	nonsynony	KLHL6:NM	NA	.	.	.	.	.	.	.	.	1	T	0.604	P	0.252	B	0	D	1	D	0.64	N	-0.17	T	-0.904	T	0.149	T	0.795	1.888	12.27	MALT_TH	TM_64	MALT_TH_	chr3:1834	1	KLHL7	-1472	-3126	NA	-1351.58	-236553%	-337949%	NA	exonic	nonsynony	KLHL6:NM	NA	.	.
MALT_TH	TM_64	MALT_TH_18_1063	chr6:137877109_G>-	1	TNFAIP3	1475	182	NA	1657	89%	11%	NA	exonic	frameshift	TNFAIP3:N	NA	.	.	.	.	.	.	.	.	.	.	.	.	.	.	.	.	.	.	.	.	.	.	.	.	.	.	.	.	.	MALT_TH	TM_64	MALT_TH_	chr6:1378	1	TNFAIP3	-1111	-2404	NA	-1104.22	-193267%	-276111%	NA	exonic	frameshift	TNFAIP3:N	NA	.	.
MALT_TH	TM_64	MALT_TH_18_1063	chr6:137878620_->C	1	TNFAIP3	3722	426	NA	4148	90%	10%	NA	exonic	frameshift	TNFAIP3:N	NA	.	.	.	.	.	.	.	.	.	.	.	.	.	.	.	.	.	.	.	.	.	.	.	.	.	.	.	.	.	MALT_TH	TM_64	MALT_TH_	chr6:1378	1	TNFAIP3	-2870	-6166	NA	-2764.9	-483885%	-691279%	NA	exonic	frameshift	TNFAIP3:N	NA	.	.
MALT_TH	TM_65	MALT_TH_18_132	chr4:105235634_G>A	1	TET2	799	95	NA	902	89%	11%	NA	exonic	stopgain	TET2:NM_	NA	.	.	.	.	.	.	.	.	1	T	.	.	.	.	0.109	N	1	D	.	.	.	.	.	.	.	.	.	7.27	38	MALT_TH	TM_65	MALT_TH_	chr4:1052	1	TET3	-609	-1313	NA	-600.898	-105184%	-150279%	NA	exonic	stopgain	TET2:NM_	NA	.	.
MALT_TH	TM_65	MALT_TH_18_132	chr9:5456165_G>A	1	CD274	135	25	NA	160	84%	16%	NA	exonic	nonsynony	CD274:NM	NA	.	.	.	.	.	.	.	.	0	D	1	D	0.998	D	0	D	1	D	2.135	M	5.13	T	-0.274	T	0.377	T	0.66	4.423	23.5	MALT_TH	TM_65	MALT_TH_	chr9:5456	1	CD275	-85	-195	NA	-106.177	-18610%	-26602%	NA	exonic	nonsynony	CD274:NM	NA	.	.
MALT_TH	TM_66	MALT_TH_18_281	chr4:105234966_G>T	1	TET2	1023	661	NA	1691	60%	39%	NA	exonic	stopgain	TET2:NM_	NA	.	.	.	.	.	.	.	.	0	D	.	.	.	.	0	N	1	D	.	.	.	.	.	.	.	.	.	6.386	37	MALT_TH	TM_66	MALT_TH_	chr4:1052	1	TET3	299	-63	NA	-1126.61	-197192%	-281722%	NA	exonic	stopgain	TET2:NM_	NA	.	.
MALT_TH	TM_66	MALT_TH_18_281	chr3:46021511_C>T	1	XCR1	272	209	NA	482	56%	43%	NA	exonic	nonsynony	XCR1:NM_	NA	.	rs1443710	.	.	.	.	.	.	0	D	0.997	D	0.814	P	0.009	N	1	N	2.415	M	1.21	T	-0.788	T	0.215	T	0.311	2.86	15.53	MALT_TH	TM_66	MALT_TH_	chr3:4602	1	XCR2	146	83	NA	-320.567	-56135%	-80213%	NA	exonic	nonsynony	XCR1:NM_	NA	.	rs144371090
MALT_TH	TM_66	MALT_TH_18_281	chr17:63929784_G>-	1	CD79B	1400	41	NA	1441	97%	3%	NA	exonic	frameshift	CD79B:NM	NA	.	.	.	.	.	.	.	.	.	.	.	.	.	.	.	.	.	.	.	.	.	.	.	.	.	.	.	.	.	MALT_TH	TM_66	MALT_TH_	chr17:6392	1	CD79B	-1318	-2677	NA	-960.305	-168079%	-240128%	NA	exonic	frameshift	CD79B:NM	NA	.	.
MALT_TH	TM_67	MALT_TH_18_427	chr4:105275372_T>-	1	TET2	897	50	NA	947	95%	5%	NA	exonic	frameshift	TET2:NM_	NA	.	.	.	.	.	.	.	.	.	.	.	.	.	.	.	.	.	.	.	.	.	.	.	.	.	.	.	.	.	MALT_TH	TM_67	MALT_TH_	chr4:1052	1	TET3	-797	-1644	NA	-630.947	-110442%	-157789%	NA	exonic	frameshift	TET2:NM_	NA	.	.
MALT_TH	TM_67	MALT_TH_18_427	chr6:137874985_TC>-	1	TNFAIP3	1724	56	NA	1780	97%	3%	NA	exonic	frameshift	TNFAIP3:N	NA	.	.	ID=COSM3	.	.	.	.	.	.	.	.	.	.	.	.	.	.	.	.	.	.	.	.	.	.	.	.	.	.	MALT_TH	TM_67	MALT_TH_	chr6:1378	1	TNFAIP4	-1612	-3280	NA	-1186.3	-207629%	-296627%	NA	exonic	frameshift	TNFAIP3:N	NA	.	.
MALT_TH	TM_68	MALT_TH_18_469	chr1:2558453_C>T	1	TNFRSF14	1155	648	NA	1812	64%	36%	NA	exonic	stopgain	TNFRSF14:	NA	.	.	.	.	.	.	.	.	1	T	.	.	.	.	.	.	1	A	.	.	.	.	.	.	.	.	.	3.144	16.51	MALT_TH	TM_68	MALT_TH_	chr1:2558	1	TNFRSF15	141	-366	NA	-1207.31	-211313%	-301895%	NA	exonic	stopgain	TNFRSF14:	NA	.	.
MALT_TH	TM_68	MALT_TH_18_469	chr12:49042326_G>A	1	KMT2D	127	38	NA	167	76%	23%	NA	exonic	nonsynony	KMT2D:NM	NA	.	rs7555737	.	.	.	.	.	.	0	D	1	D	0.997	D	0.002	N	1	D	1.67	L	-1.53	D	0.3	D	0.621	D	0.691	2.253	13.49	MALT_TH	TM_68	MALT_TH_	chr12:4904	1	KMT2D	-51	-140	NA	-110.776	-19416%	-27755%	NA	exonic	nonsynony	KMT2D:NM	NA	.	rs755573725
MALT_TH	TM_68	MALT_TH_18_469	chr4:105275056_C>T	1	TET2	734	217	NA	953	77%	23%	NA	exonic	stopgain	TET2:NM_	NA	.	rs3707356	ID=COSM4	.	.	.	.	.	1	T	.	.	.	.	.	.	1	D	.	.	.	.	.	.	.	.	.	15.416	49	MALT_TH	TM_68	MALT_TH_	chr4:1052	1	TET3	-300	-817	NA	-634.773	-111116%	-158755%	NA	exonic	stopgain	TET2:NM_	NA	.	rs370735654
MALT_TH	TM_68	MALT_TH_18_469	chrX:40057243_C>T	1	BCOR	598	184	NA	787	76%	23%	NA	exonic	nonsynony	BCOR:NM_	NA	.	.	.	.	.	.	.	.	0.01	D	1	D	0.999	D	.	.	1	D	0.385	N	-0.05	T	-0.909	T	0.165	T	0.903	5.455	35	MALT_TH	TM_68	MALT_TH_	chrX:4005	1	BCOR	-230	-644	NA	-524.102	-91748%	-131087%	NA	exonic	nonsynony	BCOR:NM_	NA	.	.
MALT_TH	TM_68	MALT_TH_18_469	chr4:105276116_G>A	1	TET2	2460	658	NA	3123	79%	21%	NA	exonic	nonsynony	TET2:NM_	NA	.	.	.	.	.	.	.	.	0	D	1	D	1	D	.	.	1	D	2.56	M	2.19	T	-0.794	T	0.17	T	0.978	4.791	27	MALT_TH	TM_68	MALT_TH_	chr4:1052	1	TET3	-1144	-2946	NA	-2081.46	-364285%	-520425%	NA	exonic	nonsynony	TET2:NM_	NA	.	.
MALT_TH	TM_68	MALT_TH_18_469	chr6:137875710_ATCT>-	1	TNFAIP3	1048	215	NA	1263	83%	17%	NA	exonic	frameshift	TNFAIP3:N	NA	.	.	.	.	.	.	.	.	.	.	.	.	.	.	.	.	.	.	.	.	.	.	.	.	.	.	.	.	.	MALT_TH	TM_68	MALT_TH_	chr6:1378	1	TNFAIP3	-618	-1451	NA	-841.496	-147291%	-210433%	NA	exonic	frameshift	TNFAIP3:N	NA	.	.
MALT_TH	TM_68	MALT_TH_18_469	chr6:137876092_->CAGATTCCTGG	1	TNFAIP3	966	134	NA	1100	88%	12%	NA	exonic	nonframes	TNFAIP3:N	NA	.	.	.	.	.	.	.	.	.	.	.	.	.	.	.	.	.	.	.	.	.	.	.	.	.	.	.	.	.	MALT_TH	TM_68	MALT_TH_	chr6:1378	1	TNFAIP3	-698	-1530	NA	-732.878	-128282%	-183276%	NA	exonic	nonframes	TNFAIP3:N	NA	.	.
MALT_TH	TM_68	MALT_TH_18_469	chr9:5463052_G>T	1	CD274	2043	523	NA	2574	79%	20%	NA	exonic	stopgain	CD274:NM	NA	.	.	.	.	.	.	.	.	0.11	T	.	.	.	.	0.144	N	1	A	.	.	.	.	.	.	.	.	.	5.209	33	MALT_TH	TM_68	MALT_TH_	chr9:5463	1	CD275	-997	-2517	NA	-1715.46	-300236%	-428926%	NA	exonic	stopgain	CD274:NM	NA	.	.
MALT_TH	TM_69	MALT_TH_18_511	chr1:2558354_A>T	1	TNFRSF14	789	232	NA	1027	77%	23%	NA	exonic	stopgain	TNFRSF14:	NA	.	.	.	.	.	.	.	.	0.9	T	.	.	.	.	.	.	1	A	.	.	.	.	.	.	.	.	.	4.64	25.5	MALT_TH	TM_69	MALT_TH_	chr1:2558	1	TNFRSF15	-325	-882	NA	-684.109	-119750%	-171088%	NA	exonic	stopgain	TNFRSF14:	NA	.	.
MALT_TH	TM_69	MALT_TH_18_511	chr19:3179088_A>G	1	S1PR4	244	246	NA	494	49%	50%	NA	exonic	nonsynony	S1PR4:NM	NA	.	.	.	.	.	.	.	.	0	D	1	D	1	D	0	D	1	D	2.025	M	-0.57	T	-0.063	T	0.467	T	0.664	3.991	20.4	MALT_TH	TM_69	MALT_TH_	chr19:3179	1	S1PR5	248	250	NA	-328.505	-57526%	-82201%	NA	exonic	nonsynony	S1PR4:NM	NA	.	.
MALT_TH	TM_69	MALT_TH_18_511	chr3:177051530_->C	1	TBL1XR1	1342	181	NA	1523	88%	12%	NA	exonic	frameshift	TBL1XR1:N	NA	.	.	.	.	.	.	.	.	.	.	.	.	.	.	.	.	.	.	.	.	.	.	.	.	.	.	.	.	.	MALT_TH	TM_69	MALT_TH_	chr3:1770	1	TBL1XR2	-980	-2141	NA	-1014.88	-177632%	-253776%	NA	exonic	frameshift	TBL1XR1:N	NA	.	.
MALT_TH	TM_69	MALT_TH_18_511	chr4:105241380_->T	1	TET2	235	24	NA	259	91%	9%	NA	exonic	frameshift	TET2:NM_	NA	.	.	.	.	.	.	.	.	.	.	.	.	.	.	.	.	.	.	.	.	.	.	.	.	.	.	.	.	.	MALT_TH	TM_69	MALT_TH_	chr4:1052	1	TET2	-187	-398	NA	-172.241	-30169%	-43115%	NA	exonic	frameshift	TET2:NM_	NA	.	.
MALT_TH	TM_69	MALT_TH_18_511	chr4:105272693_A>-	1	TET2	627	84	NA	711	88%	12%	NA	exonic	frameshift	TET2:NM_	NA	.	.	.	.	.	.	.	.	.	.	.	.	.	.	.	.	.	.	.	.	.	.	.	.	.	.	.	.	.	MALT_TH	TM_69	MALT_TH_	chr4:1052	1	TET2	-459	-1002	NA	-473.549	-82899%	-118443%	NA	exonic	frameshift	TET2:NM_	NA	.	.
MALT_TH	TM_69	MALT_TH_18_511	chrX:78960519_C>A	1	P2RY10	163	27	NA	191	85%	14%	NA	exonic	nonsynony	P2RY10:NM	NA	.	.	.	.	.	.	.	.	.	.	.	.	.	.	.	.	.	.	.	.	.	.	.	.	.	.	.	.	.	MALT_TH	TM_69	MALT_TH_	chrX:7896	1	P2RY11	-109	-245	NA	-126.86	-22229%	-31772%	NA	exonic	nonsynony	P2RY10:NM	NA	.	.
MALT_TH	TM_69	MALT_TH_18_511	chr9:5465556_T>A	1	CD274	470	58	NA	528	89%	11%	NA	exonic	nonsynony	CD274:NM	NA	.	.	.	.	.	.	.	.	0	D	0.9	P	0.332	B	0.59	N	1	N	1.355	L	4.99	T	-1.001	T	0.082	T	0.397	1.902	12.32	MALT_TH	TM_69	MALT_TH_	chr9:5465	1	CD275	-354	-766	NA	-351.557	-61550%	-87945%	NA	exonic	nonsynony	CD274:NM	NA	.	.
MALT_TH	TM_70	MALT_TH_18_534	chr4:105272622_A>G	1	TET2	3284	352	NA	3639	90%	10%	NA	exonic	nonsynony	TET2:NM_	NA	.	.	.	.	.	.	.	.	0	D	1	D	0.999	D	.	.	1	D	2.82	M	2.47	T	-0.796	T	0.149	T	0.952	4.831	27.4	MALT_TH	TM_70	MALT_TH_	chr4:1052	1	TET2	-2580	-5512	NA	-2425.57	-424502%	-606447%	NA	exonic	nonsynony	TET2:NM_	NA	.	.
MALT_TH	TM_70	MALT_TH_18_534	chr4:105275869_AA>-	1	TET2	3668	428	NA	4096	90%	10%	NA	exonic	frameshift	TET2:NM_	NA	.	.	.	.	.	.	.	.	.	.	.	.	.	.	.	.	.	.	.	.	.	.	.	.	.	.	.	.	.	MALT_TH	TM_70	MALT_TH_	chr4:1052	1	TET2	-2812	-6052	NA	-2730.23	-477818%	-682612%	NA	exonic	frameshift	TET2:NM_	NA	.	.
MALT_TH	TM_70	MALT_TH_18_534	chr17:7673546_A>G	1	TP53	1060	1071	NA	2135	50%	50%	NA	exonic	nonsynony	TP53:NM_	NA	.	.	.	.	.	.	.	.	0.24	T	0.971	D	0.85	P	0	D	1	D	2.235	M	-4.23	D	1.056	D	0.983	D	0.789	4.57	24.8	MALT_TH	TM_70	MALT_TH_	chr17:7673	1	TP54	1082	1093	NA	-1422.5	-248975%	-355700%	NA	exonic	nonsynony	TP53:NM_	NA	.	.
MALT_TH	TM_70	MALT_TH_18_534	chr9:5463055_A>-	1	CD274	4537	546	NA	5083	89%	11%	NA	exonic	frameshift	CD274:NM	NA	.	.	.	.	.	.	.	.	.	.	.	.	.	.	.	.	.	.	.	.	.	.	.	.	.	.	.	.	.	MALT_TH	TM_70	MALT_TH_	chr9:5463	1	CD275	-3445	-7436	NA	-3388.23	-592967%	-847112%	NA	exonic	frameshift	CD274:NM	NA	.	.
MALT_TH	TM_71	MALT_TH_18_823	chr1:2559922_G>A	1	TNFRSF14	99	93	NA	193	51%	48%	NA	exonic	nonsynony	TNFRSF14:	NA	.	.	.	.	.	.	.	.	0	D	1	D	0.997	D	.	.	1	N	2.19	M	-0.1	T	-0.649	T	0.396	T	0.861	2.763	15.2	MALT_TH	TM_71	MALT_TH_	chr1:2559	1	TNFRSF15	87	81	NA	-127.853	-22411%	-32037%	NA	exonic	nonsynony	TNFRSF14:	NA	.	.
MALT_TH	TM_71	MALT_TH_18_823	chr4:105235778_->G	1	TET2	266	101	NA	367	72%	28%	NA	exonic	frameshift	TET2:NM_	NA	.	.	.	.	.	.	.	.	.	.	.	.	.	.	.	.	.	.	.	.	.	.	.	.	.	.	.	.	.	MALT_TH	TM_71	MALT_TH_	chr4:1052	1	TET2	-64	-229	NA	-244.058	-42742%	-61078%	NA	exonic	frameshift	TET2:NM_	NA	.	.
MALT_TH	TM_71	MALT_TH_18_823	chr4:105275077_C>T	1	TET2	327	183	NA	513	64%	36%	NA	exonic	stopgain	TET2:NM_	NA	.	.	ID=COSM9	.	.	.	.	.	0.4	T	.	.	.	.	.	.	1	D	.	.	.	.	.	.	.	.	.	14.63	48	MALT_TH	TM_71	MALT_TH_	chr4:1052	1	TET2	39	-105	NA	-341.312	-59763%	-85396%	NA	exonic	stopgain	TET2:NM_	NA	.	.
MALT_TH	TM_71	MALT_TH_18_823	chr9:136496583_G>A	1	NOTCH1	143	324	NA	472	30%	69%	NA	exonic	stopgain	NOTCH1:N	NA	.	.	.	.	.	.	.	.	0.89	T	.	.	.	.	0.034	N	1	D	.	.	.	.	.	.	.	.	.	11.234	44	MALT_TH	TM_71	MALT_TH_	chr9:1364	1	NOTCH2	505	686	NA	-313.65	-54931%	-78496%	NA	exonic	stopgain	NOTCH1:N	NA	.	.
MALT_TH	TM_71	MALT_TH_18_823	chr6:167137253_C>A	1	CCR6	711	81	NA	796	89%	10%	NA	exonic	stopgain	CCR6:NM_	NA	.	.	.	.	.	.	.	.	1	T	.	.	.	.	0.016	U	1	D	.	.	.	.	.	.	.	.	.	5.359	34	MALT_TH	TM_71	MALT_TH_	chr6:1671	1	CCR7	-549	-1179	NA	-530.233	-92818%	-132613%	NA	exonic	stopgain	CCR6:NM_	NA	.	.
MALT_TH	TM_71	MALT_TH_18_823	chrX:71616408_G>T	1	CXCR3	264	108	NA	372	71%	29%	NA	exonic	stopgain	CXCR3:NM	NA	.	.	.	.	.	.	.	.	0.02	D	.	.	.	.	.	.	1	D	.	.	.	.	-0.264	T	0.379	T	.	6.232	37	MALT_TH	TM_71	MALT_TH_	chrX:7161	1	CXCR4	-48	-204	NA	-247.376	-43323%	-61909%	NA	exonic	stopgain	CXCR3:NM	NA	.	.
MALT_TH	TM_72	MALT_TH_18_89	chr1:2556666_T>C	1	TNFRSF14	217	76	NA	294	74%	26%	NA	exonic	nonsynony	TNFRSF14:	NA	.	rs7532686	.	.	.	.	.	.	0	D	0.967	D	0.879	P	.	.	1	D	.	.	-2.29	D	0.268	D	0.716	D	0.766	2.019	12.71	MALT_TH	TM_72	MALT_TH_	chr1:2556	1	TNFRSF15	-65	-206	NA	-195.409	-34228%	-48915%	NA	exonic	nonsynony	TNFRSF14:	NA	.	rs753268655
MALT_TH	TM_72	MALT_TH_18_89	chr12:49039863_G>C	1	KMT2D	1038	136	NA	1176	88%	12%	NA	exonic	stopgain	KMT2D:NM	NA	.	.	.	.	.	.	.	.	0	D	.	.	.	.	0.204	N	1	A	.	.	.	.	.	.	.	.	.	13.994	48	MALT_TH	TM_72	MALT_TH_	chr12:4903	1	KMT2D	-766	-1668	NA	-783.552	-137149%	-195944%	NA	exonic	stopgain	KMT2D:NM	NA	.	.
MALT_TH	TM_72	MALT_TH_18_89	chr4:105236838_C>-	1	TET2	1111	337	NA	1448	77%	23%	NA	exonic	frameshift	TET2:NM_	NA	.	.	.	.	.	.	.	.	.	.	.	.	.	.	.	.	.	.	.	.	.	.	.	.	.	.	.	.	.	MALT_TH	TM_72	MALT_TH_	chr4:1052	1	TET2	-437	-1211	NA	-964.767	-168865%	-241253%	NA	exonic	frameshift	TET2:NM_	NA	.	.
MALT_TH	TM_72	MALT_TH_18_89	chr4:105259681_G>T	1	TET2	1706	455	NA	2162	79%	21%	NA	exonic	nonsynony	TET2:NM_	NA	.	.	ID=COSM4	.	.	.	.	.	0	D	0.999	D	0.982	D	.	.	1	D	2.81	M	3.09	T	-0.962	T	0.094	T	0.969	4.617	25.2	MALT_TH	TM_72	MALT_TH_	chr4:1052	1	TET2	-796	-2047	NA	-1440.79	-252168%	-360258%	NA	exonic	nonsynony	TET2:NM_	NA	.	.
MALT_TH	TM_72	MALT_TH_18_89	chr7:2944528_C>G	1	CARD11	375	100	NA	478	78%	21%	NA	exonic	nonsynony	CARD11:N	NA	.	.	.	.	.	.	.	.	0.03	D	0.998	D	0.965	D	0	D	1	D	2.215	M	1.01	T	-0.632	T	0.229	T	0.697	3.896	19.81	MALT_TH	TM_72	MALT_TH_	chr7:2944	1	CARD12	-175	-450	NA	-318.126	-55702%	-79592%	NA	exonic	nonsynony	CARD11:N	NA	.	.
MALT_TH	TM_72	MALT_TH_18_89	chr6:137875701_A>G	1	TNFAIP3	1170	317	NA	1488	79%	21%	NA	exonic	nonsynony	TNFAIP3:N	NA	.	.	.	.	.	.	.	.	0	D	1	D	0.999	D	0	D	1	D	2.675	M	1.19	T	-0.637	T	0.203	T	0.988	5.224	33	MALT_TH	TM_72	MALT_TH_	chr6:1378	1	TNFAIP3	-536	-1389	NA	-991.454	-173535%	-247924%	NA	exonic	nonsynony	TNFAIP3:N	NA	.	.
MALT_TH	TM_72	MALT_TH_18_89	chr6:137875718_A>T	1	TNFAIP3	1169	319	NA	1489	79%	21%	NA	exonic	stopgain	TNFAIP3:N	NA	.	.	.	.	.	.	.	.	1	T	.	.	.	.	0.005	N	1	A	.	.	.	.	.	.	.	.	.	6.289	37	MALT_TH	TM_72	MALT_TH_	chr6:1378	1	TNFAIP3	-531	-1381	NA	-992.119	-173651%	-248091%	NA	exonic	stopgain	TNFAIP3:N	NA	.	.
MALT_TH	TM_72	MALT_TH_18_89	chr9:5465535_ACTT>-	1	CD274	357	52	NA	409	87%	13%	NA	exonic	frameshift	CD274:NM	NA	.	.	.	.	.	.	.	.	.	.	.	.	.	.	.	.	.	.	.	.	.	.	.	.	.	.	.	.	.	MALT_TH	TM_72	MALT_TH_	chr9:5465	1	CD275	-253	-558	NA	-272.206	-47664%	-68108%	NA	exonic	frameshift	CD274:NM	NA	.	.
MALT_TH	TM_73	MALT_TH_G0411	chr12:49034584_G>A	1	KMT2D	141	32	NA	174	81%	18%	NA	exonic	stopgain	KMT2D:NM	NA	.	.	.	.	.	.	.	.	0	D	.	.	.	.	0.003	N	1	A	.	.	.	.	.	.	.	.	.	18.46	51	MALT_TH	TM_73	MALT_TH_	chr12:4903	1	KMT2D	-77	-186	NA	-115.485	-20239%	-28930%	NA	exonic	stopgain	KMT2D:NM	NA	.	.
MALT_TH	TM_73	MALT_TH_G0411	chr1:9717608_C>G	1	PIK3CD	222	44	NA	267	83%	16%	NA	exonic	nonsynony	PIK3CD:NM	NA	.	.	.	.	.	.	.	.	0.05	D	1	D	0.992	D	0	D	1	D	2.955	M	-0.59	T	0.122	D	0.498	T	0.22	4.401	23.3	MALT_TH	TM_73	MALT_TH_	chr1:9717	1	PIK3CD	-134	-312	NA	-177.503	-31092%	-44434%	NA	exonic	nonsynony	PIK3CD:NM	NA	.	.
MALT_TH	TM_73	MALT_TH_G0411	chr9:5462862_TTTGGTTG>-	1	CD274	331	130	NA	465	71%	28%	NA	exonic	frameshift	CD274:NM	NA	.	.	.	.	.	.	.	.	.	.	.	.	.	.	.	.	.	.	.	.	.	.	.	.	.	.	.	.	.	MALT_TH	TM_73	MALT_TH_	chr9:5462	1	CD275	-71	-272	NA	-309.39	-54175%	-77411%	NA	exonic	frameshift	CD274:NM	NA	.	.
MALT_TH	TM_74	MALT_TH_G3545	chr1:2560713_A>T	2	TNFRSF14	104	46	NA	155	67%	30%	NA	exonic	stopgain	TNFRSF14:	TRUE	.	.	.	.	.	.	.	.	0.03	D	.	.	.	.	.	.	1	A	.	.	-2.56	D	-0.185	T	0.565	D	.	2.851	15.49	MALT_TH	TM_74	MALT_TH_	chr1:2560	2	TNFRSF15	-12	-70	NA	-102.714	-18007%	-25742%	NA	exonic	stopgain	TNFRSF14:	TRUE	.	.
MALT_TH	TM_74	MALT_TH_G3545	chr12:49053558_C>T	2	KMT2D	1060	205	NA	1268	84%	16%	NA	exonic	nonsynony	KMT2D:NM	TRUE	.	rs7751852	.	.	.	.	.	.	0	D	1	D	0.993	D	0.003	N	0.933	D	2.39	M	-2.26	D	0.832	D	0.788	D	0.744	2.401	13.99	MALT_TH	TM_74	MALT_TH_	chr12:4905	2	KMT2D	-650	-1505	NA	-844.839	-147876%	-211268%	NA	exonic	nonsynony	KMT2D:NM	TRUE	.	rs775185225
MALT_TH	TM_74	MALT_TH_G3545	chr4:105275569_C>T	2	TET2	1056	204	NA	1264	84%	16%	NA	exonic	stopgain	TET2:NM_	TRUE	.	.	ID=COSM4	.	.	.	.	.	0.12	T	.	.	.	.	.	.	1	D	.	.	.	.	.	.	.	.	.	16.4	50	MALT_TH	TM_74	MALT_TH_	chr4:1052	2	TET2	-648	-1500	NA	-842.173	-147409%	-210601%	NA	exonic	stopgain	TET2:NM_	TRUE	.	.
MALT_TH	TM_74	MALT_TH_G3545	chr4:105276128_T>C	2	TET2	931	221	NA	1159	80%	19%	NA	exonic	nonsynony	TET2:NM_	TRUE	.	rs1165193	ID=COSM4	.	.	.	.	.	0	D	1	D	0.999	D	.	.	1	D	2.56	M	2.3	T	-0.886	T	0.135	T	0.933	4.207	21.8	MALT_TH	TM_74	MALT_TH_	chr4:1052	2	TET2	-489	-1199	NA	-772.145	-135155%	-193095%	NA	exonic	nonsynony	TET2:NM_	TRUE	.	rs116519314
MALT_TH	TM_74	MALT_TH_G3545	chr9:5463081_A>-	2	CD274	703	93	NA	796	88%	12%	NA	exonic	frameshift	CD274:NM	TRUE	.	.	.	.	.	.	.	.	.	.	.	.	.	.	.	.	.	.	.	.	.	.	.	.	.	.	.	.	.	MALT_TH	TM_74	MALT_TH_	chr9:5463	2	CD275	-517	-1127	NA	-530.216	-92816%	-132610%	NA	exonic	frameshift	CD274:NM	TRUE	.	.
MEITL	MEITL_1	MEITL_1180_16	chr22:41157235_C>T	1	EP300	223	181	NA	404	55%	45%	NA	exonic	stopgain	EP300:NM_	NA	.	.	.	.	.	.	.	.	0.18	T	.	.	.	.	0	D	1	A	.	.	.	.	.	.	.	.	.	17.786	51	MEITL	MEITL_1	MEITL_11	chr22:4115	1	EP301	139	97	NA	-268.552	-47033%	-67210%	NA	exonic	stopgain	EP300:NM	NA	.	.
MEITL	MEITL_2	MEITL_16061_15	chr3:46373446_TTTCCATACAGTC	2	CCR5	202	56	NA	258	78%	22%	NA	exonic	frameshift	CCR5:NM_	NA	.	.	.	.	.	.	.	.	.	.	.	.	.	.	.	.	.	.	.	.	.	.	.	.	.	.	.	.	.	MEITL	MEITL_2	MEITL_16	chr3:4637	2	CCR6	-90	-236	NA	-171.45	-30034%	-42923%	NA	exonic	frameshift	CCR5:NM_	NA	.	.
MEITL	MEITL_2	MEITL_16061_15	chr17:7674894_G>A	1	TP53	96	1552	NA	1652	6%	94%	NA	exonic	stopgain	TP53:NM_	NA	.	rs3975164	ID=COSM9	Pathogeni	Li-Fraumer	RCV00003	MedGen:C	C0085390:	1	T	.	.	.	.	0	D	1	A	.	.	.	.	.	.	.	.	.	6.04	37	MEITL	MEITL_2	MEITL_16	chr17:7674	1	TP54	3008	4464	NA	-1100.06	-192559%	-275112%	NA	exonic	stopgain	TP53:NM_	NA	.	rs397516436
MEITL	MEITL_3	MEITL_16299_18	chr12:49052293_CCTCAGGTGGTG	3	KMT2D	679	806	NA	1485	46%	54%	NA	exonic	nonframes	KMT2D:NM	NA	.	rs7643645	.	.	.	.	.	.	.	.	.	.	.	.	.	.	.	.	.	.	.	.	.	.	.	.	.	.	.	MEITL	MEITL_3	MEITL_16	chr12:4905	3	KMT2D	933	1060	NA	-989.124	-173135%	-247358%	NA	exonic	nonframes	KMT2D:NM	NA	.	rs764364594
MEITL	MEITL_3	MEITL_16299_18	chr1:202194188_C>G	1	LGR6	282	323	NA	606	47%	53%	NA	exonic	nonsynony	LGR6:NM_	NA	.	rs7573753	.	.	.	.	.	.	0.18	T	0.985	D	0.831	P	0.167	U	1	D	1.32	L	3.61	T	-0.936	T	0.02	T	0.148	2.631	14.76	MEITL	MEITL_3	MEITL_16	chr1:2021	1	LGR7	364	405	NA	-403.134	-70587%	-100860%	NA	exonic	nonsynony	LGR6:NM_	NA	.	rs757375300
MEITL	MEITL_3	MEITL_16299_18	chr14:100239450_->CCACCA	1	YY1	1633	965	NA	2598	63%	37%	NA	exonic	nonframes	YY1:NM_0	NA	.	rs7652869	.	.	.	.	.	.	.	.	.	.	.	.	.	.	.	.	.	.	.	.	.	.	.	.	.	.	.	MEITL	MEITL_3	MEITL_16	chr14:1002	1	YY2	297	-371	NA	-1731.3	-303011%	-432892%	NA	exonic	nonframes	YY1:NM_0	NA	.	rs765286940
MEITL	MEITL_4	MEITL_20186_18	chrX:78273076_T>-	1	CYSLTR1	0	57	NA	57	0%	100%	NA	exonic	frameshift	CYSLTR1:N	NA	.	.	ID=COSM1	.	.	.	.	.	.	.	.	.	.	.	.	.	.	.	.	.	.	.	.	.	.	.	.	.	.	MEITL	MEITL_4	MEITL_20	chrX:7827	1	CYSLTR2	114	171	NA	-36.6667	-6467%	-9267%	NA	exonic	frameshift	CYSLTR1:N	NA	.	.
MEITL	MEITL_4	MEITL_20186_18	chr3:177050484_G>A	1	TBL1XR1	349	209	NA	559	62%	37%	NA	exonic	nonsynony	TBL1XR1:N	NA	.	.	.	.	.	.	.	.	0.1	T	0.998	D	0.979	D	0	D	1	D	0.585	N	-0.2	T	-0.497	T	0.298	T	0.897	5.253	33	MEITL	MEITL_4	MEITL_20	chr3:1770	1	TBL1XR2	69	-71	NA	-371.96	-65127%	-93059%	NA	exonic	nonsynony	TBL1XR1:N	NA	.	.
MEITL	MEITL_4	MEITL_20186_18	chr17:42680071_A>G	1	CCR10	169	170	NA	340	50%	50%	NA	exonic	nonsynony	CCR10:NM	NA	.	.	.	.	.	.	.	.	0	D	0.936	P	0.564	P	0.052	N	0.994	N	3.885	H	0.02	T	-0.358	T	0.4	T	0.554	2.73	15.09	MEITL	MEITL_4	MEITL_20	chr17:4268	1	CCR11	171	172	NA	-225.834	-39558%	-56533%	NA	exonic	nonsynony	CCR10:NM	NA	.	.
MEITL	MEITL_5	MEITL_25817_18	chr1:9718902_A>C	1	PIK3CD	712	579	NA	1293	55%	45%	NA	exonic	nonsynony	PIK3CD:NM	NA	.	.	.	.	.	.	.	.	0.21	T	0.999	D	0.98	D	0	D	1	D	1.48	L	-0.55	T	-0.254	T	0.436	T	0.592	4.351	22.9	MEITL	MEITL_5	MEITL_25	chr1:9718	1	PIK3CD	446	313	NA	-861.219	-150750%	-215377%	NA	exonic	nonsynony	PIK3CD:NM	NA	.	.
MEITL	MEITL_5	MEITL_25817_18	chr17:7675993_C>A	1	TP53	173	929	NA	1103	16%	84%	NA	splicing	.	.	NA	.	.	ID=COSM3	.	.	.	.	.	.	.	.	.	.	.	.	.	1	D	.	.	.	.	.	.	.	.	.	3.955	20.2	MEITL	MEITL_5	MEITL_25	chr17:7675	1	TP54	1685	2441	NA	-734.158	-128524%	-183632%	NA	splicing	.	.	NA	.	.
MEITL	MEITL_6	MEITL_9966_14	chr12:49048736_C>A	1	KMT2D	272	50	NA	323	84%	15%	NA	exonic	stopgain	KMT2D:NM	NA	.	.	.	.	.	.	.	.	0	D	.	.	.	.	0.003	N	1	A	.	.	.	.	.	.	.	.	.	10.119	43	MEITL	MEITL_6	MEITL_99	chr12:4904	1	KMT2D	-172	-394	NA	-214.846	-37627%	-53769%	NA	exonic	stopgain	KMT2D:NM	NA	.	.
SMZL	SMZL_1	SMZL_5235	chr12:49053503_G>C	1	KMT2D	1301	1149	NA	2456	53%	47%	NA	exonic	nonsynony	KMT2D:NM	NA	.	rs7551986	.	.	.	.	.	.	0	D	0.999	D	0.958	D	0.004	N	0.873	D	2.14	M	-2.43	D	0.838	D	0.779	D	0.647	1.612	11.35	SMZL	SMZL_104	SMZL_157	chr12:4905	1	KMT2D	997	845	NA	-1636.53	-286430%	-409207%	NA	exonic	nonsynony	KMT2D:NM	NA	.	rs755198692
SMZL	SMZL_1	SMZL_5235	chr3:177033047_C>A	1	TBL1XR1	1486	549	NA	2044	73%	27%	NA	exonic	nonsynony	TBL1XR1:N	NA	.	.	.	.	.	.	.	.	0.02	D	0.928	P	0.751	P	0	D	1	D	2.715	M	-0.18	T	-0.085	T	0.425	T	0.967	5.218	33	SMZL	SMZL_104	SMZL_157	chr3:1770	1	TBL1XR2	-388	-1325	NA	-1362.07	-238393%	-340580%	NA	exonic	nonsynony	TBL1XR1:N	NA	.	.
SMZL	SMZL_2	SMZL_6793	chr22:41178913_A>G	1	EP300	1357	919	NA	2276	60%	40%	NA	exonic	nonsynony	EP300:NM	NA	.	rs7580110	.	.	.	.	.	.	0.04	D	0.842	P	0.185	B	0	D	0.993	D	1.245	L	-2.03	D	-0.068	T	0.431	T	0.694	2.078	12.91	SMZL	SMZL_135	SMZL_203	chr22:4117	1	EP301	481	43	NA	-1516.6	-265439%	-379219%	NA	exonic	nonsynony	EP300:NM	NA	.	rs758011045
SMZL	SMZL_2	SMZL_6793	chr9:136496493_G>A	1	NOTCH1	1188	829	NA	2026	59%	41%	NA	exonic	stopgain	NOTCH1:N	NA	.	.	ID=COSM2	.	.	.	.	.	0.77	T	.	.	.	.	0.015	U	1	D	.	.	.	.	.	.	.	.	.	12.072	45	SMZL	SMZL_135	SMZL_203	chr9:1364	1	NOTCH2	470	111	NA	-1349.93	-236272%	-337552%	NA	exonic	stopgain	NOTCH1:N	NA	.	.
SMZL	SMZL_2	SMZL_6793	chr14:102870332_T>-	1	TRAF3	1049	54	NA	1103	95%	5%	NA	exonic	frameshift	TRAF3:NM	NA	.	.	.	.	.	.	.	.	.	.	.	.	.	.	.	.	.	.	.	.	.	.	.	.	.	.	.	.	.	SMZL	SMZL_135	SMZL_203	chr14:1028	1	TRAF4	-941	-1936	NA	-734.951	-128643%	-183790%	NA	exonic	frameshift	TRAF3:NM	NA	.	.
SMZL	SMZL_3	SMZL_6934	chr3:177033107_C>T	1	TBL1XR1	961	362	NA	1325	73%	27%	NA	exonic	stopgain	TBL1XR1:N	NA	.	.	.	.	.	.	.	.	1	T	.	.	.	.	0	D	1	A	.	.	.	.	.	.	.	.	.	9.304	42	SMZL	SMZL_138	SMZL_207	chr3:1770	1	TBL1XR1	-237	-836	NA	-882.727	-154509%	-220745%	NA	exonic	stopgain	TBL1XR1:N	NA	.	.
SMZL	SMZL_3	SMZL_6934	chr3:177038377_T>A	1	TBL1XR1	552	282	NA	838	66%	34%	NA	exonic	nonsynony	TBL1XR1:N	NA	.	.	.	.	.	.	.	.	0	D	0.981	D	0.863	P	0	D	1	D	3.245	M	-2.7	D	0.933	D	0.859	D	0.978	4.551	24.6	SMZL	SMZL_138	SMZL_207	chr3:1770	1	TBL1XR1	12	-258	NA	-557.998	-97683%	-139566%	NA	exonic	nonsynony	TBL1XR1:N	NA	.	.
SMZL	SMZL_3	SMZL_6934	chr7:2944528_C>T	1	CARD11	1162	163	NA	1327	88%	12%	NA	exonic	nonsynony	CARD11:N	NA	.	rs5715175	ID=COSM2	Pathogeni	B-cell_exp	RCV00016	MedGen:C	CN231446:	0.17	T	0.931	P	0.659	P	0	D	1	D	2.215	M	1.12	T	-0.845	T	0.168	T	0.739	4.2	21.8	SMZL	SMZL_138	SMZL_207	chr7:2944	1	CARD11	-836	-1835	NA	-884.211	-154765%	-221109%	NA	exonic	nonsynony	CARD11:N	NA	.	rs571517555
SMZL	SMZL_3	SMZL_6934	chr7:2937981_C>A	1	CARD11	1166	128	NA	1296	90%	10%	NA	exonic	nonsynony	CARD11:N	NA	.	.	.	.	.	.	.	.	0.01	D	1	D	0.999	D	0	D	1	D	1.995	M	1.27	T	-0.685	T	0.215	T	0.912	4.694	26	SMZL	SMZL_138	SMZL_207	chr7:2937	1	CARD11	-910	-1948	NA	-863.568	-151152%	-215947%	NA	exonic	nonsynony	CARD11:N	NA	.	.
SMZL	SMZL_4	SMZL_8415	chr19:16326026_C>T	1	KLF2	3014	246	NA	3268	92%	8%	NA	exonic	nonsynony	KLF2:NM_	NA	.	.	.	.	.	.	.	.	0	D	1	D	0.997	D	.	.	1	D	3.125	M	-0.27	T	0.272	D	0.535	D	0.696	3.529	18.01	SMZL	SMZL_168	SMZL_252	chr19:1632	1	KLF3	-2522	-5290	NA	-2178.26	-381222%	-544618%	NA	exonic	nonsynony	KLF2:NM_	NA	.	.
SMZL	SMZL_4	SMZL_8415	chr19:50770769_G>C	1	GPR32	922	800	NA	1725	53%	46%	NA	exonic	nonsynony	GPR32:NM	NA	.	.	.	.	.	.	.	.	0	D	1	D	1	D	.	.	1	D	0	N	0.6	T	-0.747	T	0.163	T	0.745	4.106	21.1	SMZL	SMZL_168	SMZL_252	chr19:5077	1	GPR33	678	556	NA	-1149.2	-201147%	-287374%	NA	exonic	nonsynony	GPR32:NM	NA	.	.
SMZL	SMZL_4	SMZL_8415	chr1:119915847_TTCCCTTCAGGT	1	NOTCH2	3634	332	NA	3966	92%	8%	NA	exonic	frameshift	NOTCH2:N	NA	.	.	.	.	.	.	.	.	.	.	.	.	.	.	.	.	.	.	.	.	.	.	.	.	.	.	.	.	.	SMZL	SMZL_168	SMZL_252	chr1:1199	1	NOTCH3	-2970	-6272	NA	-2643.58	-462654%	-660950%	NA	exonic	frameshift	NOTCH2:N	NA	.	.
SMZL	SMZL_4	SMZL_8415	chr14:102891409_->A	1	TRAF3	2919	538	NA	3457	84%	16%	NA	exonic	frameshift	TRAF3:NM	NA	.	.	.	.	.	.	.	.	.	.	.	.	.	.	.	.	.	.	.	.	.	.	.	.	.	.	.	.	.	SMZL	SMZL_168	SMZL_252	chr14:1028	1	TRAF4	-1843	-4224	NA	-2304.18	-403260%	-576102%	NA	exonic	frameshift	TRAF3:NM	NA	.	.
SMZL	SMZL_5	SMZL_8551	chr12:49033358_G>C	1	KMT2D	250	126	NA	376	66%	34%	NA	exonic	nonsynony	KMT2D:NM	NA	.	rs7524779	.	.	.	.	.	.	0	D	0.97	D	0.584	P	0.005	N	0.999	D	0.55	N	-1.55	D	-0.009	T	0.439	T	0.293	0.375	6.037	SMZL	SMZL_170	SMZL_256	chr12:4903	1	KMT2D	2	-122	NA	-249.998	-43783%	-62566%	NA	exonic	nonsynony	KMT2D:NM	NA	.	rs752477960
SMZL	SMZL_5	SMZL_8551	chr3:177046132_AAT>-	1	TBL1XR1	2385	96	NA	2481	96%	4%	NA	exonic	nonframes	TBL1XR1:N	NA	.	.	.	.	.	.	.	.	.	.	.	.	.	.	.	.	.	.	.	.	.	.	.	.	.	.	.	.	.	SMZL	SMZL_170	SMZL_256	chr3:1770	1	TBL1XR2	-2193	-4482	NA	-1653.63	-289411%	-413459%	NA	exonic	nonframes	TBL1XR1:N	NA	.	.
SMZL	SMZL_5	SMZL_8551	chr4:105235778_->G	1	TET2	1608	263	NA	1871	86%	14%	NA	exonic	frameshift	TET2:NM_	NA	.	.	.	.	.	.	.	.	.	.	.	.	.	.	.	.	.	.	.	.	.	.	.	.	.	.	.	.	.	SMZL	SMZL_170	SMZL_256	chr4:1052	1	TET3	-1082	-2427	NA	-1246.86	-218229%	-311772%	NA	exonic	frameshift	TET2:NM_	NA	.	.
SMZL	SMZL_5	SMZL_8551	chr13:32362645_C>T	1	BRCA2	954	729	NA	1691	56%	43%	NA	exonic	nonsynony	BRCA2:NM	NA	.	rs8035901	.	not provid	Familial_c	RCV00004	MedGen:C	C0346153:	0.03	D	1	D	0.999	D	0	D	1	D	2.65	M	-2.03	D	0.654	D	0.786	D	0.709	5.026	29.7	SMZL	SMZL_170	SMZL_256	chr13:3236	1	BRCA2	504	279	NA	-1126.57	-197185%	-281714%	NA	exonic	nonsynony	BRCA2:NM	NA	.	rs80359019
SMZL	SMZL_5	SMZL_8551	chr13:32362644_G>T	1	BRCA2	955	731	NA	1692	56%	43%	NA	exonic	nonsynony	BRCA2:NM	NA	.	.	.	.	.	.	.	.	0.1	T	1	D	0.998	D	0	D	1	D	2.1	M	-1.91	D	0.474	D	0.745	D	0.629	5.487	35	SMZL	SMZL_170	SMZL_256	chr13:3236	1	BRCA2	507	283	NA	-1127.24	-197302%	-281880%	NA	exonic	nonsynony	BRCA2:NM	NA	.	.
SMZL	SMZL_5	SMZL_8551	chr9:136496364_G>A	1	NOTCH1	1731	308	NA	2042	85%	15%	NA	exonic	stopgain	NOTCH1:N	NA	.	.	ID=COSM1	.	.	.	.	.	0.07	T	.	.	.	.	0	D	1	D	.	.	.	.	.	.	.	.	.	11.969	45	SMZL	SMZL_170	SMZL_256	chr9:1364	1	NOTCH2	-1115	-2538	NA	-1360.85	-238177%	-340270%	NA	exonic	stopgain	NOTCH1:N	NA	.	.
SMZL	SMZL_5	SMZL_8551	chr14:102905341_->T	1	TRAF3	1016	369	NA	1385	73%	27%	NA	exonic	frameshift	TRAF3:NM	NA	.	.	.	.	.	.	.	.	.	.	.	.	.	.	.	.	.	.	.	.	.	.	.	.	.	.	.	.	.	SMZL	SMZL_170	SMZL_256	chr14:1029	1	TRAF4	-278	-925	NA	-922.734	-161510%	-230747%	NA	exonic	frameshift	TRAF3:NM	NA	.	.
SMZL	SMZL_5	SMZL_8551	chr9:5462915_G>A	1	CD274	1638	266	NA	1911	86%	14%	NA	exonic	nonsynony	CD274:NM	NA	.	.	.	.	.	.	.	.	0	D	1	D	1	D	0	D	1	D	2.9	M	-1.56	D	0.645	D	0.755	D	0.951	4.496	24.1	SMZL	SMZL_170	SMZL_256	chr9:5462	1	CD275	-1106	-2478	NA	-1273.53	-222896%	-318439%	NA	exonic	nonsynony	CD274:NM	NA	.	.
SMZL	SMZL_6	SMZL_9237	chr12:49041696_T>A	1	KMT2D	1629	556	NA	2192	74%	25%	NA	exonic	stopgain	KMT2D:NM	NA	.	.	.	.	.	.	.	.	0	D	.	.	.	.	0.001	D	1	A	.	.	.	.	.	.	.	.	.	12.132	46	SMZL	SMZL_184	SMZL_276	chr12:4904	1	KMT2D	-517	-1590	NA	-1460.75	-255662%	-365249%	NA	exonic	stopgain	KMT2D:NM	NA	.	.
SMZL	SMZL_6	SMZL_9237	chr6:137874985_TC>-	1	TNFAIP3	2368	101	NA	2469	96%	4%	NA	exonic	frameshift	TNFAIP3:N	NA	.	.	ID=COSM3	.	.	.	.	.	.	.	.	.	.	.	.	.	.	.	.	.	.	.	.	.	.	.	.	.	.	SMZL	SMZL_184	SMZL_276	chr6:1378	1	TNFAIP3	-2166	-4433	NA	-1645.63	-288011%	-411458%	NA	exonic	frameshift	TNFAIP3:N	NA	.	.
SMZL	SMZL_6	SMZL_9237	chr6:137877138_T>-	1	TNFAIP3	1189	413	NA	1602	74%	26%	NA	exonic	frameshift	TNFAIP3:N	NA	.	.	.	.	.	.	.	.	.	.	.	.	.	.	.	.	.	.	.	.	.	.	.	.	.	.	.	.	.	SMZL	SMZL_184	SMZL_276	chr6:1378	1	TNFAIP3	-363	-1139	NA	-1067.41	-186828%	-266915%	NA	exonic	frameshift	TNFAIP3:N	NA	.	.
SMZL	SMZL_7	SMZL_C0012	chr1:202318200_G>A	1	LGR6	927	724	NA	1653	56%	44%	NA	exonic	nonsynony	LGR6:NM_	NA	.	.	ID=COSM1	.	.	.	.	.	0	D	1	D	1	D	0	D	1	D	1.895	L	-3.18	D	0.934	D	0.879	D	0.949	3.722	18.9	SMZL	SMZL_7	SMZL_C00	chr1:2023	1	LGR7	521	318	NA	-1101.23	-192751%	-275379%	NA	exonic	nonsynony	LGR6:NM_	NA	.	.
SMZL	SMZL_8	SMZL_C0013	chr3:38140768_T>C	1	MYD88	3748	892	NA	4651	81%	19%	NA	exonic	nonsynony	MYD88:NM	NA	.	.	ID=COSM8	.	.	.	.	.	0.11	T	0.998	D	0.994	D	0	D	1	D	.	.	2.18	T	-0.093	T	0.447	T	0.874	3.128	16.45	SMZL	SMZL_8	SMZL_C00	chr3:3814	1	MYD89	-1964	-4820	NA	-3100.14	-542555%	-775095%	NA	exonic	nonsynony	MYD88:NM	NA	.	.
SMZL	SMZL_9	SMZL_G0023	chr19:16326863_->C	1	KLF2	1366	349	NA	1715	80%	20%	NA	exonic	frameshift	KLF2:NM_	NA	.	.	.	.	.	.	.	.	.	.	.	.	.	.	.	.	.	.	.	.	.	.	.	.	.	.	.	.	.	SMZL	SMZL_9	SMZL_G00	chr19:1632	1	KLF3	-668	-1685	NA	-1142.8	-200019%	-285759%	NA	exonic	frameshift	KLF2:NM_	NA	.	.
SMZL	SMZL_9	SMZL_G0023	chr3:177047490_T>-	1	TBL1XR1	767	159	NA	926	83%	17%	NA	exonic	frameshift	TBL1XR1:N	NA	.	.	.	.	.	.	.	.	.	.	.	.	.	.	.	.	.	.	.	.	.	.	.	.	.	.	.	.	.	SMZL	SMZL_9	SMZL_G00	chr3:1770	1	TBL1XR2	-449	-1057	NA	-616.828	-107974%	-154266%	NA	exonic	frameshift	TBL1XR1:N	NA	.	.
SMZL	SMZL_9	SMZL_G0023	chr7:2944529_C>T	1	CARD11	1576	285	NA	1870	84%	15%	NA	exonic	nonsynony	CARD11:N	NA	.	rs3879073	ID=COSM4	Pathogeni	B-cell_exp	RCV00004	MedGen:C	CN231446:	0.03	D	0.999	D	0.978	D	0	D	1	D	2.215	M	1.01	T	-0.638	T	0.23	T	0.848	4.495	24.1	SMZL	SMZL_9	SMZL_G00	chr7:2944	1	CARD12	-1006	-2297	NA	-1246.18	-218111%	-311603%	NA	exonic	nonsynony	CARD11:N	NA	.	rs387907353
SMZL	SMZL_9	SMZL_G0023	chr3:46265497_T>G	1	CCR3	3836	884	NA	4725	81%	19%	NA	exonic	stopgain	CCR3:NM_	NA	.	.	.	.	.	.	.	.	1	T	.	.	.	.	0.001	D	1	D	.	.	.	.	.	.	.	.	.	1.653	11.49	SMZL	SMZL_9	SMZL_G00	chr3:4626	1	CCR4	-2068	-5020	NA	-3149.48	-551189%	-787429%	NA	exonic	stopgain	CCR3:NM_	NA	.	.
SMZL	SMZL_9	SMZL_G0023	chr18:62384843_A>G	1	TNFRSF11	831	329	NA	1163	71%	28%	NA	exonic	nonsynony	TNFRSF11	NA	.	rs7509847	.	.	.	.	.	.	.	.	0.278	B	0.057	B	0.578	U	0.981	N	0.895	L	.	.	-1.027	T	0.09	T	0.141	2.979	15.93	SMZL	SMZL_9	SMZL_G00	chr18:6238	1	TNFRSF11	-173	-675	NA	-774.718	-135608%	-193744%	NA	exonic	nonsynony	TNFRSF11	NA	.	rs750984705
SMZL	SMZL_10	SMZL_G0263	chr3:183494252_T>C	1	KLHL6	3193	2218	NA	5425	59%	41%	NA	exonic	nonsynony	KLHL6:NM	NA	.	.	.	.	.	.	.	.	0.45	T	0.94	P	0.601	P	0	D	1	D	2.08	M	-0.22	T	-0.541	T	0.33	T	0.896	4.554	24.6	SMZL	SMZL_10	SMZL_G02	chr3:1834	1	KLHL7	1243	268	NA	-3615.93	-632822%	-904052%	NA	exonic	nonsynony	KLHL6:NM	NA	.	.
SMZL	SMZL_10	SMZL_G0263	chrX:71616530_G>T	1	CXCR3	2409	1965	NA	4380	55%	45%	NA	exonic	nonsynony	CXCR3:NM	NA	.	.	.	.	.	.	.	.	0	D	1	D	0.999	D	0	D	0.998	D	3.765	H	-1.98	D	0.519	D	0.79	D	0.493	3.427	17.59	SMZL	SMZL_10	SMZL_G02	chrX:7161	1	CXCR4	1521	1077	NA	-2919.22	-510899%	-729877%	NA	exonic	nonsynony	CXCR3:NM	NA	.	.
SMZL	SMZL_11	SMZL_G5173	chr3:39266155_A>C	2	CX3CR1	911	1053	NA	1972	46%	53%	NA	exonic	nonsynony	CX3CR1:N	TRUE	.	.	.	.	.	.	.	.	0	D	0.995	D	0.927	D	0	D	0.999	D	3.285	M	1.16	T	-0.464	T	0.262	T	0.723	4.119	21.2	SMZL	SMZL_11	SMZL_G51	chr3:3926	2	CX3CR2	1195	1337	NA	-1313.8	-229953%	-328527%	NA	exonic	nonsynony	CX3CR1:N	TRUE	.	.
SMZL	SMZL_12	SMZL_LN11D	chr17:7673700_C>T	1	TP53	468	413	NA	881	53%	47%	NA	splicing	.	.	NA	.	.	ID=COSM2	.	.	.	.	.	.	.	.	.	.	.	.	.	1	D	.	.	.	.	.	.	.	.	.	1.157	9.71	SMZL	SMZL_12	SMZL_LN1	chr17:7673	1	TP54	358	303	NA	-586.531	-102680%	-146706%	NA	splicing	.	.	NA	.	.
SMZL	SMZL_13	SMZL_LY_449_03	chr16:3731323_AGG>-	1	CREBBP	1617	97	NA	1714	94%	6%	NA	exonic	nonframes	CREBBP:N	NA	.	rs5877835	ID=COSM2	Uncertain	Rubinstein	RCV00014	MedGen:C	C0035934:	.	.	.	.	.	.	.	.	.	.	.	.	.	.	.	.	.	.	.	.	.	SMZL	SMZL_13	SMZL_LY_	chr16:3731	1	CREBBP	-1423	-2943	NA	-1142.28	-199925%	-285622%	NA	exonic	nonframes	CREBBP:N	NA	.	rs587783503
SMZL	SMZL_14	SMZL_LY_543_09	chr1:26731445_->C	1	ARID1A	2701	78	NA	2779	97%	3%	NA	exonic	frameshift	ARID1A:NM	NA	.	.	ID=COSM5	.	.	.	.	.	.	.	.	.	.	.	.	.	.	.	.	.	.	.	.	.	.	.	.	.	.	SMZL	SMZL_14	SMZL_LY_	chr1:2673	1	ARID1A	-2545	-5168	NA	-1852.31	-324179%	-463128%	NA	exonic	frameshift	ARID1A:NM	NA	.	.
SMZL	SMZL_14	SMZL_LY_543_09	chr14:102876381_T>-	1	TRAF3	1098	40	NA	1138	96%	4%	NA	exonic	frameshift	TRAF3:NM	NA	.	.	.	.	.	.	.	.	.	.	.	.	.	.	.	.	.	.	.	.	.	.	.	.	.	.	.	.	.	SMZL	SMZL_14	SMZL_LY_	chr14:1028	1	TRAF4	-1018	-2076	NA	-758.298	-132728%	-189626%	NA	exonic	frameshift	TRAF3:NM	NA	.	.
SMZL	SMZL_15	SMZL_UH07_4293	chr19:16325961_G>A	1	KLF2	213	103	NA	318	67%	32%	NA	exonic	nonsynony	KLF2:NM_	FALSE	.	.	.	.	.	.	.	.	0	D	0.931	P	0.766	P	.	.	1	D	2.395	M	1.97	T	-0.89	T	0.113	T	0.785	3.265	16.96	SMZL	SMZL_15	SMZL_UH	chr19:1632	1	KLF2	-7	-117	NA	-211.345	-37018%	-52902%	NA	exonic	nonsynony	KLF2:NM_	FALSE	.	.
SMZL	SMZL_15	SMZL_UH07_4293	chr19:16327030_A>G	1	KLF2	669	213	NA	886	76%	24%	NA	exonic	stoploss	KLF2:NM_	FALSE	.	.	.	.	.	.	.	.	.	.	.	.	.	.	.	.	1	D	.	.	.	.	.	.	.	.	.	0.802	8.217	SMZL	SMZL_15	SMZL_UH	chr19:1632	1	KLF2	-243	-699	NA	-590.094	-103297%	-147585%	NA	exonic	stoploss	KLF2:NM_	FALSE	.	.
SMZL	SMZL_16	SMZL_UH07_5408	chr6:111333364_G>A	2	REV3L	493	121	NA	614	80%	20%	NA	exonic	nonsynony	REV3L:NM	TRUE	.	rs1437540	.	.	.	.	.	.	0	D	1	D	0.999	D	0	D	1	D	3.735	H	1.99	T	-0.434	T	0.211	T	0.988	4.827	27.4	SMZL	SMZL_16	SMZL_UH	chr6:1113	2	REV3L	-251	-623	NA	-408.803	-71570%	-102261%	NA	exonic	nonsynony	REV3L:NM	TRUE	.	rs143754029
SMZL	SMZL_16	SMZL_UH07_5408	chr17:7675224_G>C	2	TP53	188	312	NA	500	38%	62%	NA	exonic	nonsynony	TP53:NM_	TRUE	.	.	ID=COSM4	.	.	.	.	.	0	D	0.999	D	0.999	D	0	D	1	D	2.85	M	-7.31	D	0.946	D	0.994	D	0.941	3.641	18.51	SMZL	SMZL_16	SMZL_UH	chr17:7675	2	TP54	436	560	NA	-332.376	-58206%	-83175%	NA	exonic	nonsynony	TP53:NM_	TRUE	.	.
